# ZBIT Bioinformatics Toolbox: A Web-Platform for Systems Biology and Expression Data Analysis

**DOI:** 10.1371/journal.pone.0149263

**Published:** 2016-02-16

**Authors:** Michael Römer, Johannes Eichner, Andreas Dräger, Clemens Wrzodek, Finja Wrzodek, Andreas Zell

**Affiliations:** 1 Department of Computer Science, University of Tübingen, Tübingen, Germany; 2 Department of Bioengineering, University of California, San Diego, San Diego, California, United States of America; Harbin Institute of Technology Shenzhen Graduate School, CHINA

## Abstract

Bioinformatics analysis has become an integral part of research in biology. However, installation and use of scientific software can be difficult and often requires technical expert knowledge. Reasons are dependencies on certain operating systems or required third-party libraries, missing graphical user interfaces and documentation, or nonstandard input and output formats. In order to make bioinformatics software easily accessible to researchers, we here present a web-based platform. The Center for Bioinformatics Tuebingen (ZBIT) Bioinformatics Toolbox provides web-based access to a collection of bioinformatics tools developed for systems biology, protein sequence annotation, and expression data analysis. Currently, the collection encompasses software for conversion and processing of community standards SBML and BioPAX, transcription factor analysis, and analysis of microarray data from transcriptomics and proteomics studies. All tools are hosted on a customized Galaxy instance and run on a dedicated computation cluster. Users only need a web browser and an active internet connection in order to benefit from this service. The web platform is designed to facilitate the usage of the bioinformatics tools for researchers without advanced technical background. Users can combine tools for complex analyses or use predefined, customizable workflows. All results are stored persistently and reproducible. For each tool, we provide documentation, tutorials, and example data to maximize usability. The ZBIT Bioinformatics Toolbox is freely available at https://webservices.cs.uni-tuebingen.de/.

## Introduction

Systems biology, transcription factor annotation, and expression data analysis are major applications of bioinformatics. Research in those fields has yielded specialized software and methods that promote research in life sciences and have led to many biological discoveries [1–3]. However, many bioinformatics tools require advanced technical knowledge. For instance, the installation of the software itself can be difficult due to dependencies on specific requirements on the operating system or third-party libraries. Knowledge about internals of file formats may also be needed when converting files or in order to combine different tools. Many tools do not provide a graphical user interface but only a command-line interface or lack sufficient documentation for simple usage. In recent years, the advent of large-scale data has introduced new oppertunities, but also challenges in many fields of biology. A number of computational frameworks have been proposed for the handling of large-scale data, one of the most prominent being the MapReduce framework, which has been succesfully used in bioinformatics applications [4]. However, these frameworks require tailor-made software and infrastructure as well as advanced technical knowledge for installation and maintenance.

To facilitate access to tools for life science researchers, online platforms have been established that provide predefined interfaces through which tools can be remotely used. The advantage of these web-platforms is that they are installed remotely on a server. Thus, users do not need to install software on their local machine, but can access the tools through a convenient interface in the web browser. This allows the usage of tools that require lots of processing power or memory regardless of the user’s hardware configuration, because the computation is performed on the server. For this reason, the user’s device and operating system are almost irrelevant. Even mobile devices with very limited resources could be used to submit jobs through the online platform and view results in standard formats.

One example for these web-based services is Galaxy, an open, web-based platform for computational biology [5]. Galaxy was originally developed for sequence analysis, but has also been used in other fields, such as proteomics and systems biology [6, 7]. Galaxy provides an established, user-friendly interface to command-line tools. It includes user management, storage of results in accordance with scientific requirements, and allows the inclusion of custom command-line tools through XML files.

Here, we present the ZBIT Bioinformatics Toolbox, a customized Galaxy instance for systems biology, transcription factor annotation, and expression data analysis. Our online platform offers a user-friendly interface to a collection of command-line tools that have been developed at the chair of Cognitive Systems. These tools can be categorized into systems biology (BioPAX2SBML, SBMLsqueezer, SBML2LaTeX, ModelPolisher), transcription factor analysis (TFpredict, SABINE), and expression data analysis (RPPApipe, ToxDBScan).

### Available tools

#### Systems biology

The Systems Biology Markup Language (SBML) [8] and the Biological Pathway Exchange (BioPAX) format [9] belong to the most widely used community standards in systems biology [10]. For a long time, both formats were incompatible: while SBML has mainly been designed for quantitative analysis, BioPAX is optimized for exchange of qualitative pathways between databases [11]. To facilitate the exchange of models between researchers and databases, many tools have been developed to convert model formats and to add information from external databases.

BioPAX2SBML converts BioPAX models into SBML. This program was the first converter that properly translated qualitative relations [11]. BioPAX2SBML was used in the Path2Models project to create mathematical models in the SBML format from biochemical pathway maps retrieved from multiple data sources [12].

SBMLsqueezer generates kinetic equations needed for dynamic simulation from the stoichiometry, the participating species, and regulatory relations stored in a SBML model [13]. The program is also capable of retrieving experimentally determined rate laws from the SABIO-RK database [14]. SBMLsqueezer has been used in the Path2Models project to add kinetic equations to the translated SBML models [12]. Other uses of SBMLsqueezer include modeling of the MAPK machinery activation in plants [15] or simulation of drug effects using systems biology approaches [16].

**Fig 1:**
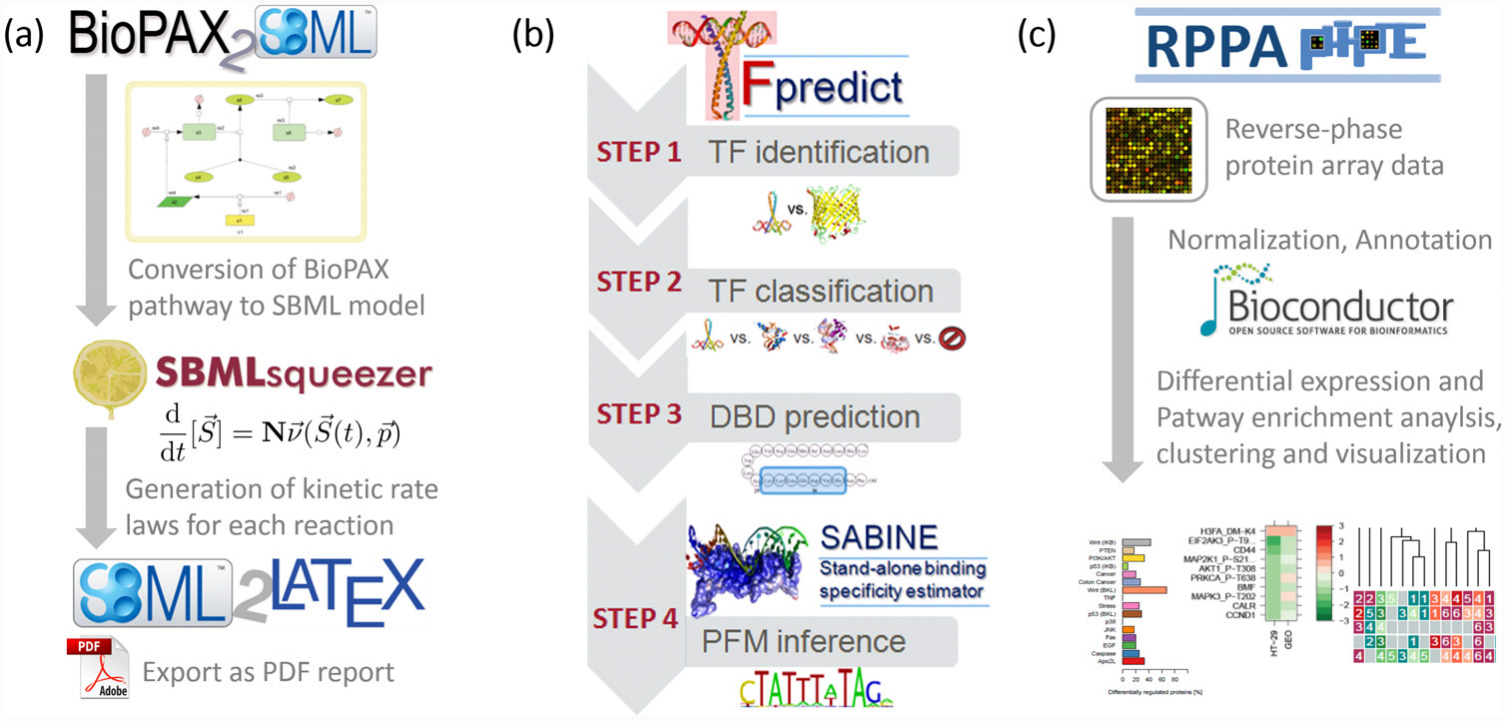
Analysis workflows available in the ZBIT Bioinformatics Toolbox. The workflows presented in this figure represent fundamental use-case scenarios, which combine tools from all three basic classes of tools within the ZBIT Toolbox. (a) SBML model processing with BioPAX2SBML, SBMLsqueezer, and SBML2LaTeX. BioPAX2SBML converts models from the BioPAX format to SBML and conserves qualitative models. SBMLsqueezer generates kinetic rate laws for each reaction contained in an SBML file. SBML2LaTeX creates human-readable reports from SBML files. (b) Transcription factor analysis using TFpredict and SABINE. TFpredict is used to identify transcription factors and predict their superclass and DNA binding domains. SABINE uses this information to infer the position frequency matrix, which represents their DNA binding profile. (c) Reverse phase protein array analysis with RPPApipe. RPPApipe implements a customizable pipeline for RPPA data analysis. This includes normalization and annotation of raw data, statistical methods for the detection of deregulated and differentially modified proteins, and their association with alterations on the pathway level, and visualization of the results.

SBML2LaTeX generates human-readable model reports from SBML files [17]. For example, SBML2LaTeX has been used in the BioModels Database to generate human-readable PDF reports for each model within this database [18, 19]. The three tools can be combined into pipelines that create SBML models and reports (see Fig 1a).

ModelPolisher takes as input SBML models that make use of conventions from the constraint-based modeling community and complements all of its components with annotations from the BiGG Models knowledgebase [20]. The application matches the identifiers of all model components against the specification of BiGG IDs (see [21]). Whenever a component has a corresponding entry in the BiGG database, ModelPolisher pulls all available metadata about that component. ModelPolisher uses BiGG IDs to recognize specific reaction and metabolite types and uses corresponding terms from the Systems Biology Ontology [22] to clearly annotate those components. It also performs basic checks in order to ensure the structural correctness of the model and displays warnings in cases such as mass balance deficiencies. The output of ModelPolisher is an updated SBML file that can be used as input for subsequent tools within the toolbox or external tools that support the SBML format.

#### Transcription factor annotation

The binding of transcription factors (TF) at defined DNA domains is essential for the regulation of genes. TFpredict identifies TFs, predicts their structural superclass given a protein sequence, and uses InterProScan to detect their DNA-binding domains (DBD) [23, 24]. TFpredict applies a sequence-based machine learning approach to predict functional characteristics trained on data from the TRANSFAC and MatBase databases [25, 26]. Eichner *et al.* showed that TFpredict performs better than previously published methods [23].

The Stand-alone binding specificity estimator (SABINE) infers the DNA motif of a TF as a position frequency matrix (PFM), based on the amino acid sequence, detected DBDs, superclass, and species [23]. SABINE uses Support Vector Regression to predict the PFM based on the similarity to other TFs with well-defined PFMs. The similarity to other TFs is established based on evolutionary, structural, and chemical similarities.

In combination, TFpredict and SABINE may be used for structural and functional annotation of TFs (see Fig 1b). For example, NR2C2 and PPARA were predicted as TFs for CYP3A4 in human hepatocytes and have been confirmed in wet-lab experiments [27].

#### Expression data analysis

High-throughput gene expression analysis has become an important part of biological research [2]. Reverse phase protein arrays (RPPAs) have been used, e.g., in individualized medicine and cancer biology [28, 29]. RPPApipe offers customizable workflows for RPPA experiments, including preprocessing, annotation, statistical analysis, clustering, pathway analysis, and visualization of results (see Fig 1c, [30]). RPPApipe supports several experimental designs: standard paired condition and control designs as well as more specialized designs with multiple conditions or replicated time-series. Particularly, RPPApipe supports a number of RPPA-specific analyses, such as evaluation of differential modification or pathway profiles that account for the lower number of analytes compared to transcriptomics studies. RPPApipe is fully compatible with InCroMAP, which allows users to integrate RPPA data with other *omics* layers, e.g., mRNA- and microRNA expression or epigenetic modifications [31].

Transcriptomics studies have recently been investigated for integration into the preclinical drug development process [32]. Two major databases for gene expression changes after short-term exposure of rodents to carcinogenic chemicals have been released for public access: Open TG-GATEs [33] and DrugMatrix [34]. ToxDBScan performs large scale similarity screening of these two databases to elucidate the carcinogenic potential and mode of action of new chemicals based on well-characterized chemicals that induce similar gene expression patterns [35]. ToxDBScan provides a similarity score to assess the relevance of carcinogenicity results for related chemicals. In addition, pathway enrichment analysis is performed to inform on possible modes of action. The similarity scoring approach has been successfully validated with external data not included in DrugMatrix and TG-GATEs [35].

### Workflow creation

The Galaxy framework allows users to combine multiple tools to complex workflows. These workflows enable users to build sophisticated pipelines by connecting analysis tools through their input and output. This is in accordance with the UNIX philosophy of writing programs that do one task and do it well to ensure modularity and reusability of tools and code. In consequence, complex tasks can be solved by the combination of simple tools.

We created workflows for common use cases that we anticipate will be useful to users of our web platform. All workflows can be saved and shared with other users. An overview of the example workflows along with descriptions is given in Table 1.

**Table 1:** Predefined workflows in the ZBIT Bioinformatics Toolbox.

Workflow name	Description	Steps
BioPAX2SBMLandSqueeze2LaTeX	Converts BioPAX files to full SBML models and human-readable reports.	3
TFpredict & SABINE	Uses TFpredict and SABINE to annotate transcription factors.	2
RPPApipe two-class	Processes data sets with paired samples obtained from RPPAs.	12
RPPApipe time-series	Processes replicated time-series data sets obtained from RPPAs.	12
RPPApipe multi-class	Processes data sets with multiple sample classes obtained from RPPAs.	12

## Methods

**Fig 2:**
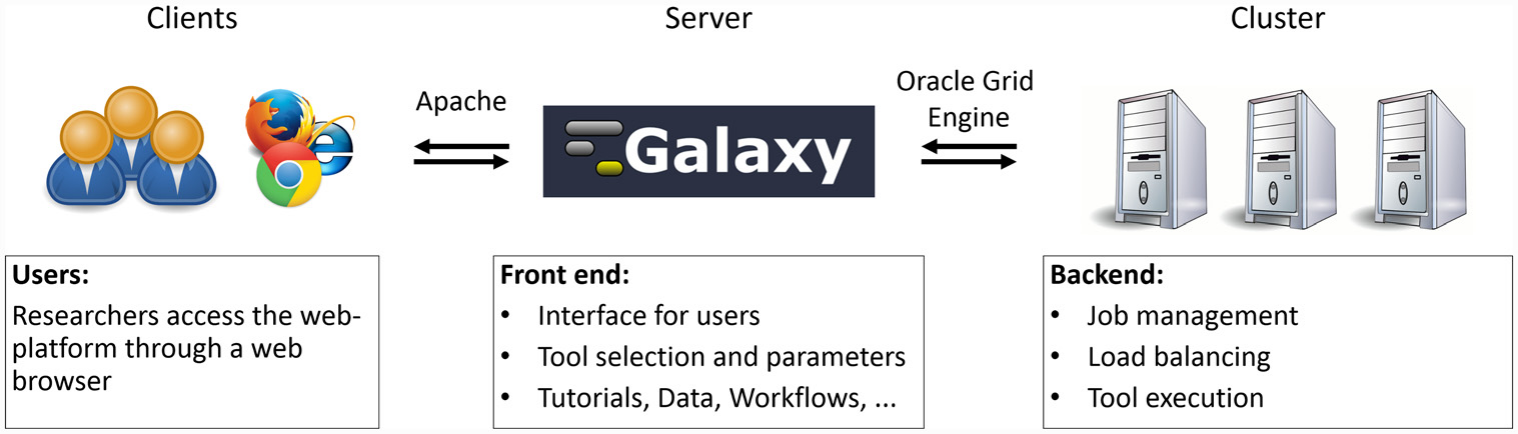
General architecture of the ZBIT Bioinformatics Toolbox. This schematic represents the ZBIT Bioinformatics Toolbox and its subsystems. First, researchers (also called clients) access the site through the internet. All in- and outgoing traffic is handled by Apache. On the server host, the Galaxy framework is used to provide the front end, i.e., the interface with which users interact to select tools, upload data, and set parameters for analysis. Galaxy also handles user management, workflows, and persistent data storage. Requested analysis are submitted to a computing cluster through the Oracle Grid Engine (OGE). OGE manages the distribution of jobs, i.e., individual analysis with a specific tool, to available nodes of the cluster and the queue of running, waiting, and finished jobs. On each cluster node, the Java™Runtime Environment or R is used to execute the actual analysis with the selected tool. After the execution finishes, results are passed back along this command chain to Galaxy. Galaxy then stores the result and displays it to the user in an appropriate format.

The ZBIT Bioinformatics Toolbox has been implemented as a web platform that is hosted on a GNU/Linux operating system. We use the open source platform Galaxy [5] to provide a common front end to the individual tools. All currently included tools are implemented in either Java™or the R language for statistical computing. A schematic overview of the system is shown in Fig 2.

### Web server setup

All requirements for the individual tools and the Galaxy platform have been installed on a machine running Ubuntu 14.04. Apache2 and Python 2.7.3 were installed from the main Ubuntu repository. Galaxy was downloaded and installed without root privileges to secure the system. We use the Oracle Grid Engine to distribute and manage the analysis on a computing cluster dedicated to the ZBIT Bioinformatics Toolbox. The computing cluster consists of three nodes running Ubuntu 14.04. On all nodes, the Java™Runtime Environment (JRE, version 1.7.0) was installed from the main Ubuntu repository. R (version 3.2.2) was installed by adding the appropriate repository provided by the R developers [36]. The command-line tools have been integrated with Galaxy through XML files and shell scripts.

## Results and Discussion

The ZBIT Bioinformatics Toolbox contains seven tools from the areas of systems biology, transcription factor annotation, and expression data analysis. Most of the tools have been developed as stand-alone, command-line tools for specific problems in the respective area. However, tools can be combined to create complex analysis, which previously required manual execution of each tool subsequently. The Galaxy front end provides a user-friendly interface to these command-line tools, without requiring the installation of the software or its dependencies. In addition, predefined and user-created workflows can be used to automate complex analysis that would have required multiple command-line tools. Where possible, the tool output is generated in standardized and established formats (e.g., SBML, PDF, CSV). For each tool, we provide extensive documentation, tutorials, example data, and predefined workflows to maximize usability for life science researchers. Furthermore, most tools are also available as stand-alone programs for download and offline use.

### Use cases

To demonstrate the usage of the ZBIT Bioinformatics Toolbox, we will shortly describe a use case for each of the three categories. We used the predefined workflows to analyze real data obtained from public repositories. All data files have also been deposited in the ZBIT Bioinformatics Toolbox for reproduction of the described use cases.

#### Creation of full kinetic models from pathway maps

**Fig 3:**
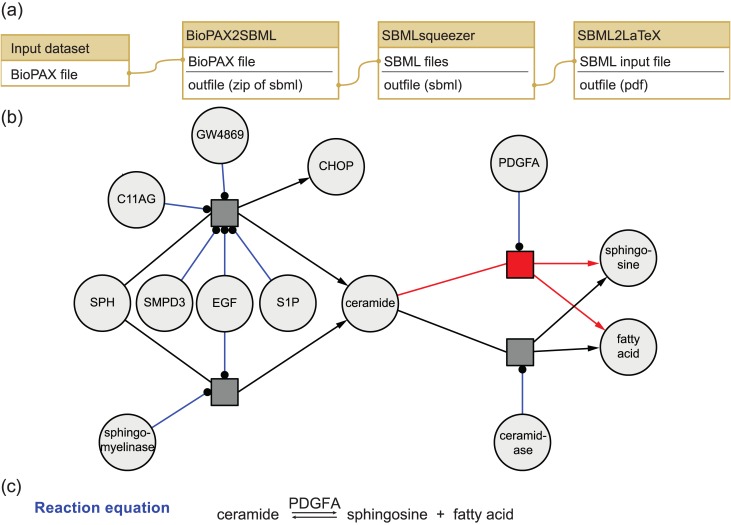
Creation of a full kinetic model for the ceramide signaling pathway. (a) Predefined Galaxy workflow for creation of kinetic models from BioPAX files. BioPAX files are used by many pathway databases to describe pathways and qualitative relations of molecules. BioPAX2SBML is used to convert the BioPAX encoded pathway to a draft SBML model. SBMLsqueezer infers reaction equations and kinetic rate laws for the relations defined in the resulting SBML model. SBML2LaTeX creates a human-readable report for model inspection to facilitate interpretation and curation. (b) Subnetwork of the SBML model of the ceramide signaling pathway. This network represents a small part of the full ceramide signaling pathway that is involved in creation and degradation of ceramide. This network contains four reversible reactions (dark gray squares) and 13 reactants. The black arrows indicate the participation of reactants in reactions. Blue lines indicate enzymatic behavior of reactants. The reaction highlighted in red degrades ceramide to sphingosine and fatty acid and is catalyzed by Platelet-derived growth factor subunit A (PDGFA). This network was created with CySBML [41] from the draft SBML model generated by SBMLsqueezer. For the full model see S2 Fig. (c) Reaction equation for ceramide degradation. SBML2LaTeX creates reaction equations for all reactions in the PDF report. This reaction degrades ceramide to sphingosine and fatty acid and is catalyzed by PDGFA. The reaction is also part of the subnetwork shown in (b) and is highlighted in red.

Ceramides are sphingolipids that are found in the cell membrane of cells. Ceramide signaling has been linked to apoptosis and programmed cell death [37, 38]. We downloaded the ceramide signaling pathway from the Pathway Interaction Database (PID) in BioPAX format (see S1 Text, [39]). A graphical representation of the pathway is shown in S1 Fig. We used the BioPAX2SBMLandSqueeze2LaTeX workflow to generate a full kinetic model stored in the community standard SBML format and a human readable PDF report (see also Table 1 and Fig 3(a)). The BioPAX2SBMLandSqueeze2LaTeX workflow consists of three steps. First, BioPAX2SBML was used to convert the BioPAX file to SBML without loss of information (see Fig 3(b)). This is achieved by using the SBML extension package for qualitative models [40]. Second, SBMLsqueezer was used to generate and add kinetic equations for all reactions in the model (see S2 Text and Fig 3(c)). Third, SBML2LaTeX was used to generate the human readable report as PDF (see S3 Text). We have used the workflow with the preset default options. In total, the created SBML model contains 50 reactions with 93 involved molecules and 263 kinetic parameters. To further customize the model or the report, SBMLsqueezer and SBML2LaTeX offer a number of user-settings to influence the program’s behavior and choices. The SBML model can be used with any modeling software that supports the SBML standard and the SBML Level 3 qual package.

#### Identification of a transcription factor and its DNA binding domain

**Fig 4:**
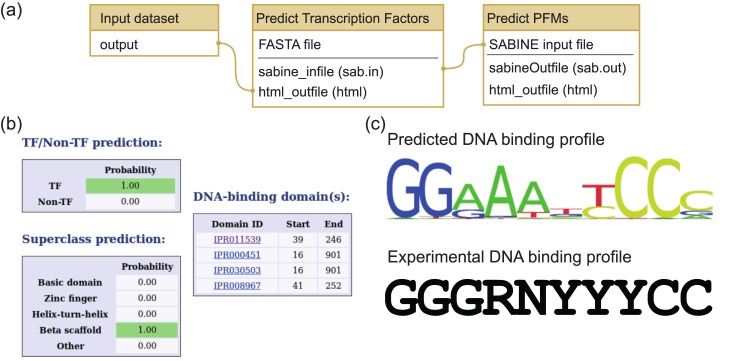
Transcription factor prediction for human NF-*κ*B with TFpredict and SABINE workflow. (a) Predefined Galaxy workflow for transcription factor annotation. The input FASTA sequence file contains the protein sequence. TFpredict uses the sequence to predict if the protein is a transcription factor, infer its superclass, and detect DNA binding domains. SABINE uses the output of TFpredict to identify the DNA sequence that is bound by the transcription factor. (b) TFpredict output for NF-*κ*B protein sequence. NF-*κ*B was correctly identified as a transcription factor. The superclass was predicted to be beta scaffold. TFpredict detected four DNA binding domains. (c) DNA binding profile predicted by SABINE. SABINE predicted a position frequency matrix with medium confidence. The predicted DNA binding profile shows good concordance with the consensus DNA binding profile established by Wan and Lenardo [44]. In the consensus sequence, R represents a purine, Y a pyrimidine, and N any nucleotide.

**Fig 5:**
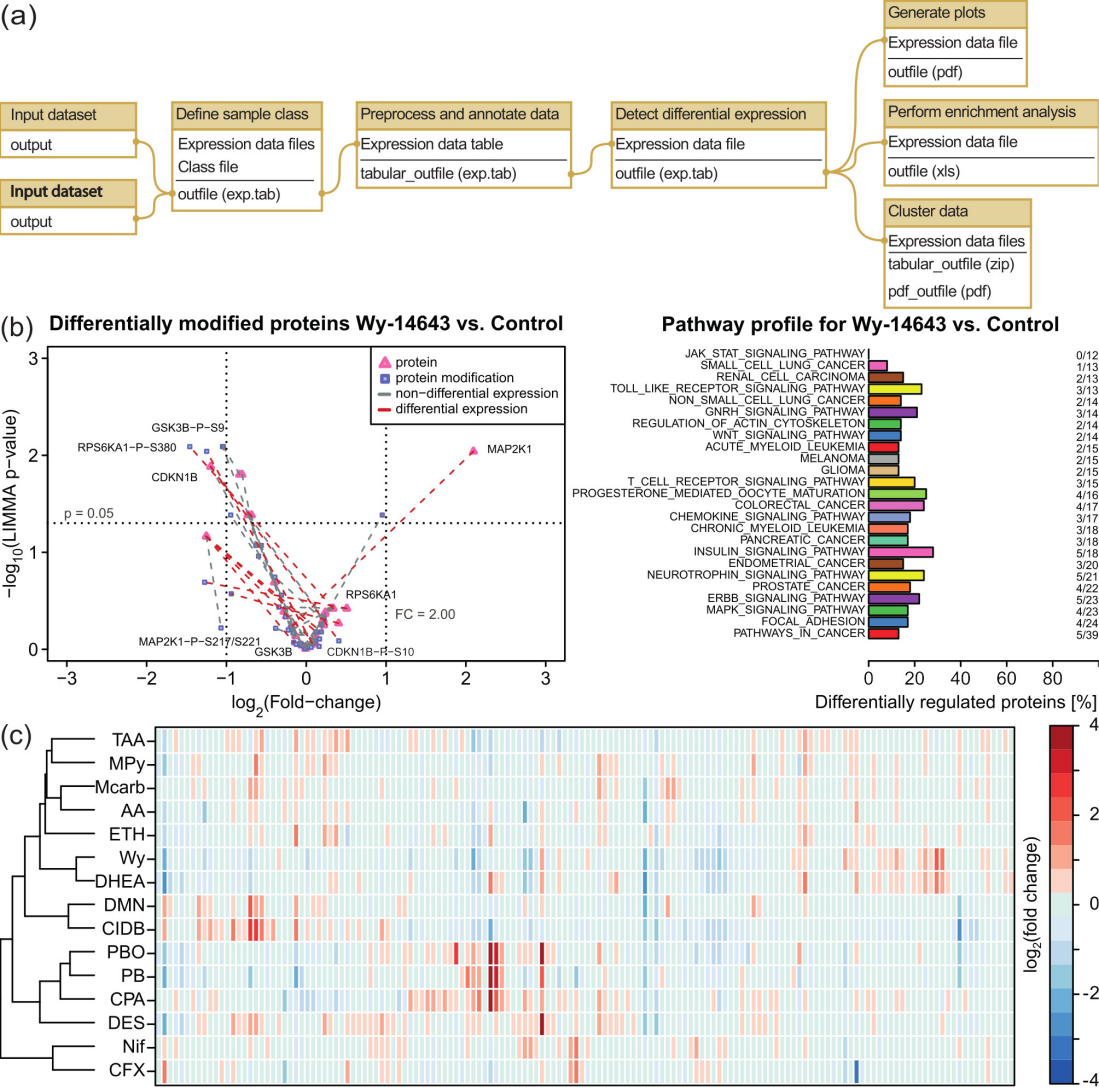
Analysis of the effects of drugs on protein expression with RPPApipe. (a) Predefined Galaxy workflow for RPPA data analysis. Two input files are required: a CSV file containing the RPPA expression values and a class file, which defines the relations between samples. First, the data is normalized and annotated. Second, differential expression of proteins is determined. Third, various plots are generated and pathway enrichment and clustering are performed. (b) Volcano plot and pathway profile for RPPA data. These example plots were generated using a data set for effects of drug exposure on the protein expression in rat liver. Several protein are differentially modified after treatment with Wy-14643, a non-genotoxic carcinogen (left). Differentially regulated proteins have been mapped to KEGG pathways to identify potential deregulation on pathway level (right). (c) Clustering of drugs by effects in rat liver. All 15 drugs in the data set were clustered by their protein expression profiles. The two non-hepatocarcinogens (Nif, CFX) formed a separate cluster from the carcinogens. The two genotoxic carcinogens (CIDB, DMN) formed a cluster within the carcinogens. The non-genotoxic carcinogens formed several clusters.

TFs are DNA-binding proteins that are involved in many biological processes in the cell nucleus, e.g., initiation of transcription at the promotor site of genes or regulation of nucleases and helicases [42]. NF-*κ*B is a human TF that is present in most cell types and is involved in many signaling events in the cell nucleus [43]. We downloaded the sequence of NF-*κ*B from UniProt as a FASTA file (UniProt ID P19838, see S4 Text) and used the TFpredict & SABINE workflow to assess if NF-*κ*B is correctly predicted as a TF (see Fig 4a). The TFpredict & SABINE workflow consists of two steps. First, TFpredict is used to predict if the input protein sequence is a TF, assign it to a superclass, and detect possible DBDs through InterProScan [24]. For NF-*κ*B, TFpredict correctly predicts that it is a TF of the beta scaffold class and identified four potential DNA binding domains (see Fig 5). Second, SABINE is used to predict the PFM of the DNA sequence that is recognized by the TF based on the identified superclass and DBDs. SABINE was able to identify the PFM with medium confidence (see Fig 4c). The predicted PFM 5’-GGRAANYCCC-3’ is in good concordance with the DNA sequence recognized by NF-*κ*B: 5’-GGGRNYYYCC-3’, where R represents a purine, Y a pyrimidine, and N any nucleotide [44].

#### Effects of drugs on protein expression

The administration of drugs may have undesired side effects. For this reason, the drug development process includes several phases for testing the side effects of candidate drugs. During the preclinical phase, *in vitro* and *in vivo* experiments are performed with animals before testing proceeds to human patients. This preclinical phase includes carcinogenicity tests, e.g., the Ames test to assess DNA damaging effects [45]. However, some chemicals are known to cause cancer through mechanisms not related to DNA damage, so called non-genotoxic carcinogens (NGCs) [46]. Currently, methods are being investigated to reliably detect NGCs early in the preclinical phase [32]. To assess the effects of NGCs, we analyzed a data set that measured the protein expression in the liver of Wistar rats *in vivo* after 14 days of chronic treatment with 11 NGCs, 2 genotoxic carcinogens (GCs), and 2 non-hepatocarcinogens (NCs) (available from GEO under GSE53084 [47, 48]). We used the RPPApipe two-class workflow (see Fig 5a and Table 1). The RPPApipe two-class workflow consists of 12 steps and requires two input files: the measured protein expression and a class definition file (see S5 Text and S6 Text, respectively). The workflow is divided into three major phases. First, the samples were assigned to treatment groups defined in the class file and preprocessed. In this case, preprocessing was done without scaling and log-transformation, as the data was already normalized. During preprocessing, additional information, e.g., gene descriptions or alternative identifiers, were fetched from public resources. Second, fold changes and *p*-values for differential expression were computed using default settings, i.e., limma [49] was used to identify differentially regulated proteins and *p*-values were corrected for multiple testing using the Benjamini-Hochberg approach [50]. Third, various plots have been generated, e.g., volcano plots for differential regulation and modification, and KEGG [51] pathway profiling was performed (see Fig 5b). The clustering shows a clear distinction between the two NCs, Nif and CFX, and carcinogens, and to a lesser degree between NGCs and the two GCs, CIDB and DMN, as can be seen in Fig 5c. This supports recent findings that suggest that integration of multiple *omics* levels can improve early assessment of carcinogenic effects [48].

### Related web platforms

Over the last years, bioinformatics tools have gained ever more importance for biological and biomedical research. The main reason is the development of new technologies, such as next-generation sequencing, *in silico* modeling of cellular processes, sequence-based protein characterization, and many more. As stated before, bioinformatics software often requires advanced technical knowledge. To this end, web platforms have been established as an easy, user-friendly interface to many bioinformatics tools. In the following, we will describe and compare some web platforms that are related to our ZBIT Bioinformatics Toolbox.

#### Systems Biology

For systems biology researchers, we provide the tools BioPAX2SBML, SBMLsqueezer, and SBML2LaTeX. To find related web platforms we used the SBML Software Guide, which is actively maintained and available from the official SBML website (sbml.org). At the time of writing, the Software Guide includes a number of web platforms for SBML editing (semanticSBML, [52]) visualization (PATIKAweb, [53]), and annotation (MetaNetX, [54]). However, none of the listed web platforms provides the functionality offered by the tools hosted on our web platform. For the conversion of BioPAX files to SBML format, the SBML Software Guide lists only BioPAX2SBML and SyBiL. In contrast to BioPAX2SBML, SyBiL does not provide a web interface and requires the installation of additional dependencies. The Software Guide lists no alternatives for the conversion to human-readable PDF reports that is offered by SBML2LaTeX, the inference of kinetic equations with SBMLsqueezer, or the model enrichment with information from the BiGG database with ModelPolisher.

#### Transcription factor annotation

Traditional, experimental methods for identifying and characterizing TFs were time-consuming and expensive. For this reason, a number of computational *in silico* methods have been proposed during the last years, among them TFpredict and SABINE, which are available from our web platform. A number of other web platforms for predicting DNA-binding proteins have been published, most notable iDNA-Prot|dis by Liu *et al.*[55] and nDNA-Prot by Song *et al.*[42] for DNA binding prediction. Both iDNA-Prot|dis and nDNA-Prot provide a simple web interface for pasting the protein sequence and predicting if the protein is DNA binding. In contrast to TFpredict, both do not attempt to identify the superclass if a protein is found to be a transcription factor. They also do not provide additional information that is provided by TFpredict, like DNA binding domains, but only report the prediction result. In a comparison with similar tools, iDNA-Prot|dis and nDNA-Prot have been shown to outperform their competitors. However, the tools have neither been compared to each other, nor to TFpredict. To our knowledge, there are no web platforms which allow the prediction of PFMs that is performed by SABINE.

#### Expression data analysis

For expression data analysis, we provide RPPApipe for analyzing protein data and ToxDBScan for gene expression data. In a recent review, Wachter *et al.*[56] compiled an overview of tools for RPPA data analysis. Among the referenced tools only two web platforms are present: RPPApipe and Miracle [57]. The other tools that are discussed by Wachter *et al.* are available as R packages or Excel macros. In addition, some of these are missing documentation or require registration prior to use. While Miracle does provide a web platform, no officially hosted web server running Miracle is available. Rather, the user is expected to set up a local instance of Miracle for his analyses, which requires the necessary infrastructure and advanced informatics skills. To our knowledge, RPPApipe is currently the only RPPA analysis pipeline that is available as a web platform which requires no set up or registration. If the user is only interested in finding patterns in the RPPA expression data, without RPPA specific analyses, there are alternative web platforms which address specific problems. For example, PaGeFinder [58] provides pattern analysis for user submitted expression data and PaGenBase provides a database of pattern genes in a number of model organsims [59]. These web platform can be used to identify genes that are specifically expressed under certain conditions, e.g., to identify spatiotemporal patters in sequential gene expression experiments. While RPPApipe does not offer specific tools for detecting spatiotemporal patterns, it provides visualizations and RPPA specific analyses that allow the identification of genes that respond to specific conditions or in specific tissues.

ToxDBScan performs a similarity search for gene expression patterns in TG-GATEs and DrugMatrix. These two databases are the largest resources on the effects of non-genotoxic, carcinogenic substances on gene expression in rats. Currently, there are two other web portals that provide similar functionality: Toxygates [60] and LTMap [61]. Toxygates is a data portal which provides exploration tools for the TG-GATEs data, allows compound ranking by gene expression and links expression data with pathology reports [60]. However, Toxygates does not provide similarity search based on differentially regulated genes provided by the user, LTMap performs similarity ranking based on user-submitted probe lists in TG-GATEs data, but does not offer any additional analyses [61]. In contrast, ToxDBScan not only offers the similarity scoring of user-submitted gene expression profiles, but also performs pathway enrichment analysis and creates visualizations that aid the interpretation of the similarity profiles. Another advantage of ToxDBScan over the other two tools is the integration of DrugMatrix data, which almost doubles the number of compounds available for similarity search.

## Conclusion

The ZBIT Bioinformatics Toolbox is an easily usable collection of online tools for systems biology, transcription factor annotation, and expression data analysis that does neither require installation of software nor advanced technical knowledge and allows the combination of tools to build custom analysis workflows. All tools and workflows are accessible from any device with a modern web browser and internet access. The tools have been applied by researchers to gain new knowledge in systems biology [15, 16, 27] and are adopted in established databases [18]. We used the Galaxy framework, which was designed with particular consideration of the requirements for scientific software, such as storage of analysis results, scalability, and reproducibility. Tutorials and example data are available for all tools. We have created predefined workflows that demonstrate the capabilities and use cases of each tool and are available through the toolbox. New tools can easily be integrated in the system with the flexible Galaxy framework and we are looking into extending the functionality by incorporating external tools that might benefit the users of the our platform, e.g., new tools for feature extraction for protein characterization, such as Pse-in-One [62]. We will continue to maintain and extend the ZBIT Bioinformatics Toolbox as new tools are developed.

## Supporting Information

S1 Fig The NCI curated ceramide signaling pathway from the Pathway Interaction Database. This is a graphical representation of the NCI curated ceramide signaling pathway obtained from the Pathway Interaction Database (PID).
(JPEG)

S2 Fig Full network of the SBML model of the ceramide signaling pathway. This network represents the full SBML model of the ceramide signaling pathway generated by BioPAX2SBML and SBMLsqueezer. Gray squares represent reactions, light gray circles reactants, black arrows participation in a reaction, blue lines indicate enzymatic behavior. The network was created with CySBML.
(PDF)

S1 Text NCI curated ceramide signaling pathway. This is the NCI curated ceramide signaling pathway from the Pathway Interaction Database in BioPAX format. The pathway is available for download in the custom PID XML format and the BioPAX community standard.
(OWL)

S2 Text Full SBML model of the ceramide signaling pathway. This full SBML model of the ceramide signaling pathway was generated by BioPAX2SBML and SBMLsqueezer. This represents a draft model, which should be checked and possibly curated before using it for simulation.
(XML)

S3 Text Human-readable report for full SBML model of the ceramide signaling pathway. This is a human-readable report that was generated with SBML2LaTeX from the full SBML model of the ceramide signaling pathway generated by BioPAX2SBML and SBMLsqueezer.
(PDF)

S4 Text FASTA sequence of human NF-*κ*B. This FASTA file contains the protein sequence of the human transcription factor NF-*κ*B obtained from UniProt (ID P19838).
(FASTA)

S5 Text Protein expression in Wistar rats after treatment with several chemicals. This CSV file contains RPPA expression data collected from the liver of Wistar rats that have been treated with one of 11 non-genotoxic carcinogens, 2 carcinogenic carcinogens, and 2 non-hepatocarcinogens. Samples have been performed in triplicates, with matched controls.
(CSV)

S6 Text Class definition file for RPPApipe. This is a plain text file that describes the relation between treated and control samples in the RPPA expression data (S5 Text).
(TXT)

## References

[1] Wasserman WW, Sandelin A. Applied bioinformatics for the identification of regulatory elements. Nature Reviews Genetics. 2004;5(4):276–287. 10.1038/nrg1315 15131651

[2] Brazma A, Vilo J. Gene expression data analysis. FEBS Letters. 2000;480(1):17–24. 10.1016/S0014-5793(00)01772-5 10967323

[3] Kitano H. Computational systems biology. Nature. 2002;420:206–10. 10.1038/nature01254 12432404

[4] Zou Q, Li XB, Jiang WR, Lin ZY, Li GL, Chen K. Survey of MapReduce frame operation in bioinformatics. Briefings in Bioinformatics. 2014;15(4):637–647. 10.1093/bib/bbs088 23396756

[5] Goecks J, Nekrutenko A, Taylor J. Galaxy: a comprehensive approach for supporting accessible, reproducible, and transparent computational research in the life sciences. Genome Biology. 2010;11(8):R86 10.1186/gb-2010-11-8-r86 20738864 PMC2945788

[6] Narang P, Khan S, Hemrom A, Lynn A. MetaNET—a web-accessible interactive platform for biological metabolic network analysis. BMC Systems Biology. 2014;8(130). 10.1186/s12918-014-0130-2 25779921 PMC4267457

[7] Hildebrandt AK, Stockel D, Fischer NM, de la Garza L, Kruger J, Nickels S, et al ballaxy: web services for structural bioinformatics. Bioinformatics. 2015;31(1):121–122. 10.1093/bioinformatics/btu574 25183489

[8] Hucka M, Finney A, Sauro HM, Bolouri H, Doyle JC, Kitano H, et al The systems biology markup language (SBML): a medium for representation and exchange of biochemical network models. Bioinformatics. 2003;19(4):524–531. 10.1093/bioinformatics/btg015 12611808

[9] Demir E, Cary MP, Paley S, Fukuda K, Lemer C, Vastrik I, et al The BioPAX community standard for pathway data sharing. Nature Biotechnology. 2010;28(9):935–942. 10.1038/nbt.1666 20829833 PMC3001121

[10] Dräger A, Palsson BØO. Improving Collaboration by Standardization Efforts in Systems Biology. Frontiers in Bioengineering and Biotechnology. 2014;2:61. 25538939 10.3389/fbioe.2014.00061 PMC4259112

[11] Büchel F, Wrzodek C, Mittag F, Dräger A, Eichner J, Rodriguez N, et al Qualitative translation of relations from BioPAX to SBML qual. Bioinformatics. 2012;28(20):2648–2653. 10.1093/bioinformatics/bts508 22923304 PMC3467751

[12] Büchel F, Rodriguez N, Swainston N, Wrzodek C, Czauderna T, Keller R, et al Path2Models: large-scale generation of computational models from biochemical pathway maps. BMC Systems Biology. 2013;7(116). 10.1186/1752-0509-7-116 PMC4228421 24180668

[13] Dräger A, Hassis N, Supper J, Schröder A, Zell A. SBMLsqueezer: a CellDesigner plug-in to generate kinetic rate equations for biochemical networks. BMC Systems Biology. 2008;2(39). 10.1186/1752-0509-2-39 18447902 PMC2412839

[14] Wittig U, Kania R, Golebiewski M, Rey M, Shi L, Jong L, et al SABIO-RK–database for biochemical reaction kinetics. Nucleic Acids Research. 2012;40(D1):D790–D796. 10.1093/nar/gkr1046 22102587 PMC3245076

[15] Pathak RK, Taj G, Pandey D, Arora S, Kumar A. Modeling of the MAPK machinery activation in response to various abiotic and biotic stresses in plants by a system biology approach. Bioinformation. 2013;9(9):443–449. 10.6026/97320630009443 23847397 PMC3705613

[16] Gupta MK, Misra K. Modeling and simulation analysis of propyl-thiouracil (PTU), an anti-thyroid drug on thyroid peroxidase (TPO), thyroid stimulating hormone receptor (TSHR), and sodium iodide (NIS) symporter based on systems biology approach. Network Modeling Analysis in Health Informatics and Bioinformatics. 2013;2(1):45–57. 10.1007/s13721-013-0023-0

[17] Dräger A, Planatscher H, Motsou Wouamba D, Schröder A, Hucka M, Endler L, et al SBML2L(A)T(E)X: conversion of SBML files into human-readable reports. Bioinformatics. 2009;25(11):1455–1456. 10.1093/bioinformatics/btp170 19307240 PMC2682517

[18] Li C, Donizelli M, Rodriguez N, Dharuri H, Endler L, Chelliah V, et al BioModels Database: An enhanced, curated and annotated resource for published quantitative kinetic models. BMC Systems Biology. 2010;4(92). 10.1186/1752-0509-4-92 PMC2909940 20587024

[19] Chelliah V, Juty N, Ajmera I, Ali R, Dumousseau M, Glont M, et al BioModels: ten-year anniversary. Nucleic Acids Research. 2015;43(D1):D542–D548. 10.1093/nar/gku1181 25414348 PMC4383975

[20] King ZA, Lu J, Dräger A, Miller P, Federowicz S, Lerman JA, et al BiGG Models: A platform for integrating, standardizing and sharing genome-scale models. Nucleic Acids Research. 2015;p. gkv1049. 10.1093/nar/gkv1049 PMC4702785 26476456

[21] King ZA. BiGG Models ID Specification and Guidelines; 2015. Available from: https://github.com/SBRG/bigg_models/wiki/BiGG-Models-ID-Specification-and-Guidelines.

[22] Courtot M, Juty N, Knupfer C, Waltemath D, Zhukova A, Drager A, et al Controlled vocabularies and semantics in systems biology. Molecular Systems Biology. 2011;7:543–543. 10.1038/msb.2011.77 22027554 PMC3261705

[23] Eichner J, Topf F, Dräger A, Wrzodek C, Wanke D, Zell A. TFpredict and SABINE: sequence-based prediction of structural and functional characteristics of transcription factors. PLoS ONE. 2013;8(12):e82238 10.1371/journal.pone.0082238 24349230 PMC3861411

[24] Jones P, Binns D, Chang HY, Fraser M, Li W, McAnulla C, et al InterProScan 5: genome-scale protein function classification. Bioinformatics. 2014;30(9):1236–1240. 10.1093/bioinformatics/btu031 24451626 PMC3998142

[25] Matys V. TRANSFAC(R): transcriptional regulation, from patterns to profiles. Nucleic Acids Research. 2003;31(1):374–378. 10.1093/nar/gkg108 12520026 PMC165555

[26] Cartharius K, Frech K, Grote K, Klocke B, Haltmeier M, Klingenhoff A, et al MatInspector and beyond: promoter analysis based on transcription factor binding sites. Bioinformatics. 2005;21(13):2933–2942. 10.1093/bioinformatics/bti473 15860560

[27] Schröder A, Wollnik J, Wrzodek C, Dräger A, Bonin M, Burk O, et al Inferring statin-induced gene regulatory relationships in primary human hepatocytes. Bioinformatics. 2011;27(18):2473–2477. 21757465 10.1093/bioinformatics/btr416

[28] Gallagher RI, Espina V. Reverse phase protein arrays: mapping the path towards personalized medicine. Molecular Diagnosis & Therapy. 2014;18(6):619–630. 10.1007/s40291-014-0122-3 25358623 PMC4732707

[29] Unterberger EB, Eichner J, Wrzodek C, Lempiäinen H, Luisier R, Terranova R, et al Ha-ras and *β*-catenin oncoproteins orchestrate metabolic programs in mouse liver tumors. International Journal of Cancer. 2014;135(7):1574–1585. 10.1002/ijc.28798 24535843

[30] Eichner J, Heubach Y, Ruff M, Kohlhof H, Strobl S, Mayer B, et al RPPApipe: A pipeline for the analysis of reverse-phase protein array data. Biosystems. 2014;122:19–24. 10.1016/j.biosystems.2014.06.009 24951946

[31] Wrzodek C, Eichner J, Büchel F, Zell A. InCroMAP: integrated analysis of cross-platform microarray and pathway data. Bioinformatics (Oxford, England). 2013;29(4):506–8. 10.1093/bioinformatics/bts709 PMC3570209 23257199

[32] Waters MD, Jackson M, Lea I. Characterizing and predicting carcinogenicity and mode of action using conventional and toxicogenomics methods. Mutation Research. 2010;705(3):184–200. 10.1016/j.mrrev.2010.04.005 20399889

[33] Uehara T, Ono A, Maruyama T, Kato I, Yamada H, Ohno Y, et al The Japanese toxicogenomics project: application of toxicogenomics. Molecular Nutrition & Food Research. 2010;54(2):218–227. 10.1002/mnfr.200900169 20041446

[34] Ganter B, Tugendreich S, Pearson CI, Ayanoglu E, Baumhueter S, Bostian KA, et al Development of a large-scale chemogenomics database to improve drug candidate selection and to understand mechanisms of chemical toxicity and action. Journal of Biotechnology. 2005;119(3):219–244. 16005536 10.1016/j.jbiotec.2005.03.022

[35] Römer M, Backert L, Eichner J, Zell A. ToxDBScan: Large-Scale Similarity Screening of Toxicological Databases for Drug Candidates. International Journal of Molecular Sciences. 2014;15(10):19037–19055. 10.3390/ijms151019037 25338045 PMC4227259

[36] R Core Team. R: A Language and Environment for Statistical Computing. Vienna, Austria; 2015. Available from: https://www.r-project.org/.

[37] Haimovitz-Friedman A, Kolesnick RN, Fuks Z. Ceramide signaling in apoptosis. British Medical Bulletin. 1997;53(3):539–553. 10.1093/oxfordjournals.bmb.a011629 9374036

[38] Obeid L, Linardic C, Karolak L, Hannun Y. Programmed cell death induced by ceramide. Science. 1993;259(5102):1769–1771. 10.1126/science.8456305 8456305

[39] Schaefer CF, Anthony K, Krupa S, Buchoff J, Day M, Hannay T, et al PID: the Pathway Interaction Database. Nucleic Acids Research. 2009;37(suppl 1):D674–D679. 10.1093/nar/gkn653 18832364 PMC2686461

[40] Chaouiya C, Bérenguier D, Keating SM, Naldi A, van Iersel MP, Rodriguez N, et al SBML qualitative models: a model representation format and infrastructure to foster interactions between qualitative modelling formalisms and tools. BMC Systems Biology. 2013;7(1):135 10.1186/1752-0509-7-135 24321545 PMC3892043

[41] Konig M, Drager A, Holzhutter HG. CySBML: a Cytoscape plugin for SBML. Bioinformatics. 2012;28(18):2402–2403. 10.1093/bioinformatics/bts432 22772946

[42] Song L, Li D, Zeng X, Wu Y, Guo L, Zou Q. nDNA-prot: identification of DNA-binding proteins based on unbalanced classification. BMC Bioinformatics. 2014;15(1):298 10.1186/1471-2105-15-298 25196432 PMC4165999

[43] The UniProt Consortium. UniProt: a hub for protein information. Nucleic Acids Research. 2015;43(D1):D204–D212. 10.1093/nar/gku989 25348405 PMC4384041

[44] Wan F, Lenardo MJ. Specification of DNA binding activity of NF-kappaB proteins. Cold Spring Harbor Perspectives in Biology. 2009;1(4):a000067 10.1101/cshperspect.a000067 20066093 PMC2773628

[45] Ames BN, McCann J, Yamasaki E. Methods for detecting carcinogens and mutagens with the salmonella/mammalian-microsome mutagenicity test. Mutation Research. 1975;31(6):347–363. 768755 10.1016/0165-1161(75)90046-1

[46] Silva Lima B, Van der Laan JW. Mechanisms of nongenotoxic carcinogenesis and assessment of the human hazard. Regulatory Toxicology and Pharmacology. 2000;32(2):135–43. 11067770 10.1006/rtph.2000.1427

[47] Edgar R. Gene Expression Omnibus: NCBI gene expression and hybridization array data repository. Nucleic Acids Research. 2002;30(1):207–210. 10.1093/nar/30.1.207 11752295 PMC99122

[48] Römer M, Eichner J, Metzger U, Templin MF, Plummer S, Ellinger-Ziegelbauer H, et al Cross-platform toxicogenomics for the prediction of non-genotoxic hepatocarcinogenesis in rat. PLoS ONE. 2014;9(5):e97640 10.1371/journal.pone.0097640 24830643 PMC4022579

[49] Smyth GK. limma: Linear Models for Microarray Data In: Gentleman R, Carey VJ, Huber W, Irizarry RA, Dudoit S, editors. Bioinformatics and Computational Biology Solutions Using R and Bioconductor. Statistics for Biology and Health. New York: Springer-Verlag; 2005 p. 397–420.

[50] Benjamini Y, Hochberg Y. Controlling the false discovery rate: a practical and powerful approach to multiple testing. Journal of the Royal Statistical Society. 1995;57(1):289–300.

[51] Kanehisa M, Goto S. KEGG: kyoto encyclopedia of genes and genomes. Nucleic acids research. 2000;28(1):27–30. 10.1093/nar/28.1.27 10592173 PMC102409

[52] Krause F, Uhlendorf J, Lubitz T, Schulz M, Klipp E, Liebermeister W. Annotation and merging of SBML models with semanticSBML. Bioinformatics. 2010;26(3):421–422. 10.1093/bioinformatics/btp642 19933161

[53] Dogrusoz U, Erson EZ, Giral E, Demir E, Babur O, Cetintas A, et al PATIKAweb: a Web interface for analyzing biological pathways through advanced querying and visualization. Bioinformatics. 2006;22(3):374–375. 10.1093/bioinformatics/bti776 16287939

[54] Ganter M, Bernard T, Moretti S, Stelling J, Pagni M. MetaNetX.org: a website and repository for accessing, analysing and manipulating metabolic networks. Bioinformatics. 2013;29(6):815–816. 10.1093/bioinformatics/btt036 23357920 PMC3597148

[55] Liu B, Xu J, Lan X, Xu R, Zhou J, Wang X, et al iDNA-Prot|dis: Identifying DNA-Binding Proteins by Incorporating Amino Acid Distance-Pairs and Reduced Alphabet Profile into the General Pseudo Amino Acid Composition. PLoS ONE. 2014;9(9):e106691 10.1371/journal.pone.0106691 25184541 PMC4153653

[56] Wachter A, Bernhardt S, Beissbarth T, Korf U. Analysis of Reverse Phase Protein Array Data: From Experimental Design towards Targeted Biomarker Discovery. Microarrays. 2015;4(4):520–539. 10.3390/microarrays4040520 27600238 PMC4996411

[57] List M, Block I, Pedersen ML, Christiansen H, Schmidt S, Thomassen M, et al Microarray R-based analysis of complex lysate experiments with MIRACLE. Bioinformatics. 2014;30(17):i631–i638. 10.1093/bioinformatics/btu473 25161257 PMC4147925

[58] Pan JB, Hu SC, Wang H, Zou Q, Ji ZL. PaGeFinder: quantitative identification of spatiotemporal pattern genes. Bioinformatics. 2012;28(11):1544–1545. 10.1093/bioinformatics/bts169 22492640 PMC3356841

[59] Pan JB, Hu SC, Shi D, Cai MC, Li YB, Zou Q, et al PaGenBase: A Pattern Gene Database for the Global and Dynamic Understanding of Gene Function. PLoS ONE. 2013;8(12):e80747 10.1371/journal.pone.0080747 24312499 PMC3846610

[60] Nystrom-Persson J, Igarashi Y, Ito M, Morita M, Nakatsu N, Yamada H, et al Toxygates: interactive toxicity analysis on a hybrid microarray and linked data platform. Bioinformatics. 2013;29(23):3080–3086. 10.1093/bioinformatics/btt531 24048354

[61] Xing L, Wu L, Liu Y, Ai N, Lu X, Fan X. LTMap: a web server for assessing the potential liver toxicity by genome-wide transcriptional expression data. Journal of Applied Toxicology. 2014;34(7):805–809. 10.1002/jat.2923 24022982

[62] Liu B, Liu F, Wang X, Chen J, Fang L, Chou KC. Pse-in-One: a web server for generating various modes of pseudo components of DNA, RNA, and protein sequences. Nucleic Acids Research. 2015;43(W1):W65–W71. 10.1093/nar/gkv458 25958395 PMC4489303

