# Supplementary figures and images for: ZBIT Bioinformatics Toolbox: A Web-Platform for Systems Biology and Expression Data Analysis
Source: PLoS One. 2016 Feb 16;11(2):e0149263. doi: 10.1371/journal.pone.0149263 (PMC4801062; doi:10.1371/journal.pone.0149263)

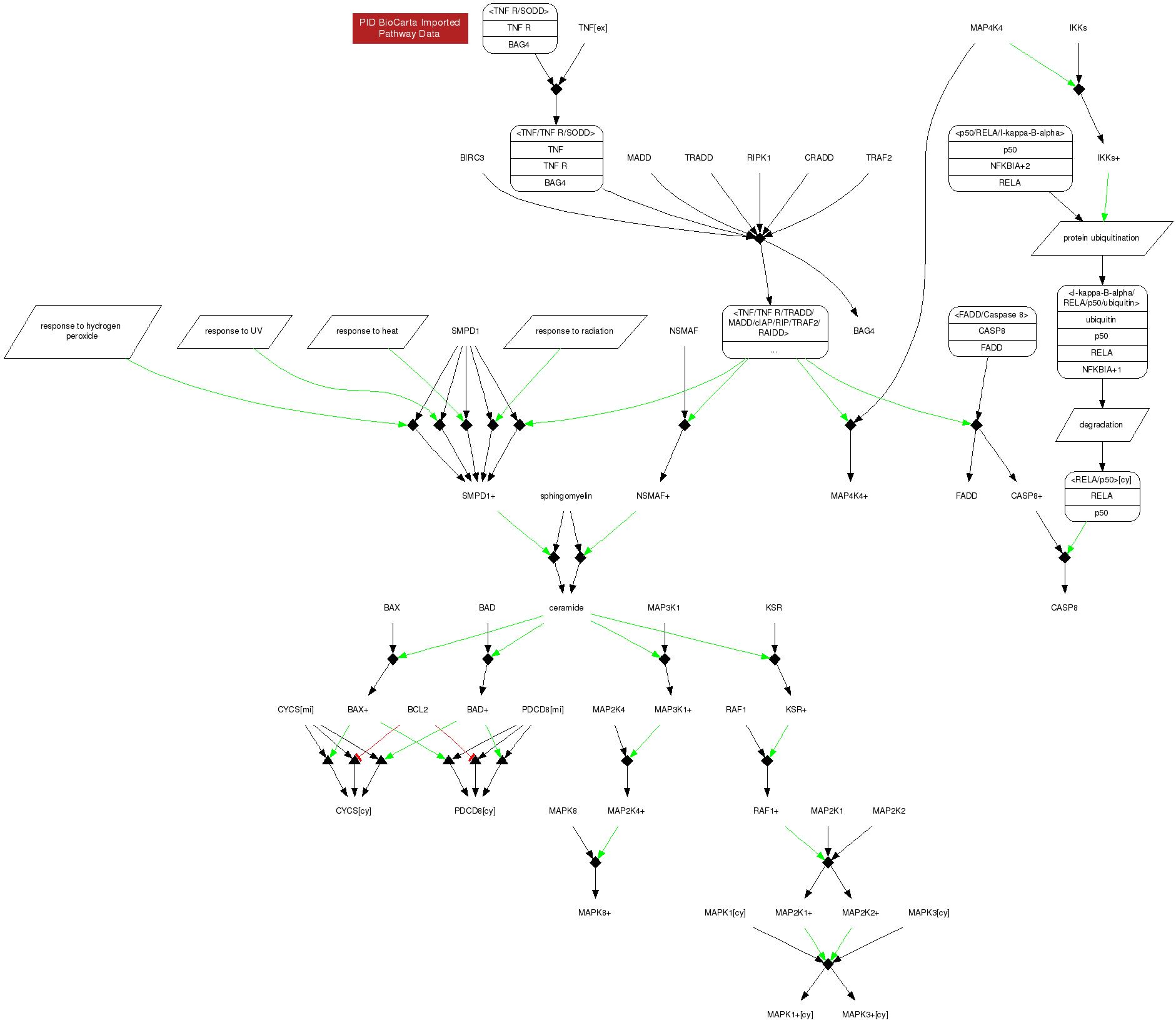

Supplement: S1 Fig — This is a graphical representation of the NCI curated ceramide signaling pathway obtained from the Pathway Interaction Database (PID). (JPEG) [file pone.0149263.s001.jpeg]

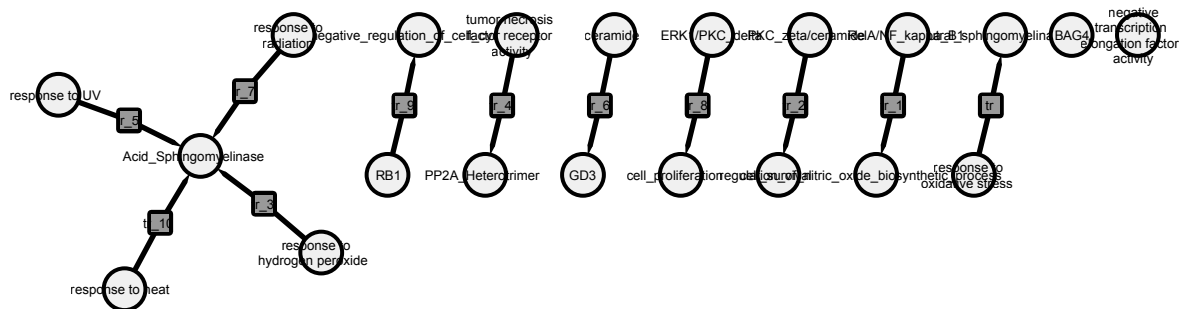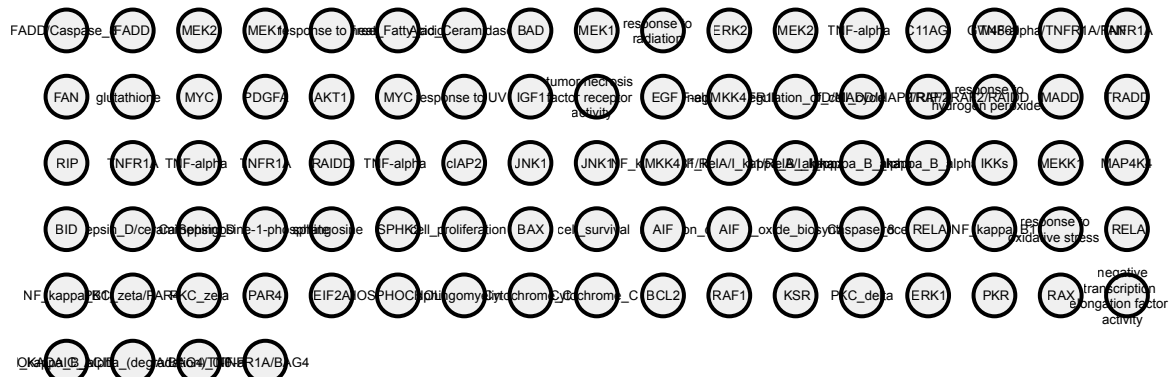

Supplement: S2 Fig — This network represents the full SBML model of the ceramide signaling pathway generated by BioPAX2SBML and SBMLsqueezer. Gray squares represent reactions, light gray circles reactants, black arrows participation in a reaction, blue lines indicate enzymatic behavior. The network was created with CySBML. (PDF) [file pone.0149263.s002.pdf]
