# Supplementary material for: ZBIT Bioinformatics Toolbox: A Web-Platform for Systems Biology and Expression Data Analysis
Source: PLoS One. 2016 Feb 16;11(2):e0149263. doi: 10.1371/journal.pone.0149263 (PMC4801062; doi:10.1371/journal.pone.0149263)
Supplement: S3 Text — This is a human-readable report that was generated with SBML2LaTeX from the full SBML model of the ceramide signaling pathway generated by BioPAX2SBML and SBMLsqueezer. (PDF) [file pone.0149263.s005.pdf]

SBML Model Report

Model name: “Ceramide signaling pathway (Homo sapiens (human))”

May 22, 2015

1 General Overview

This is a document in SBML Level 3 Version 1 format. This Model was created by and ZBIT, University of T?bingen, WSI-CogSys .

Table **??** gives an overview of the quantities of all components of this model.

Table 1: The SBML components in this model.
All components are described in more detail in the following sections.

Element	Quantity	Element	Quantity
Compartment types	0	Compartments	5
Species types	0	Species	93
Events	0	Constraints	0
Reactions	50	Function definitions	0
Parameters	263	Unit definitions	5
Rules	0	Initial assignments	0

Model notes

Model of ?Ceramide signaling pathway? in ?Homo sapiens (human)?

BioPAX comment: ?BioPAX output created 2012_09_17 12:21::40, converted from the Pathway Interaction Database, National Cancer Institute, http://pid.nci.nih.gov.?

This model has been automatically generated by BioPAX2SBML V1.0.1Original entry

Model Annotation

The following resources provide further information about this model:

This model is described by urn:miriam:pubmed:21700675.

This biological entity occurs in urn:miriam:taxonomy:9606.

2 Unit definitions

This is an overview of five Unit definitions.

2.1 Unit definition time

Name Predefined unit time

Definition s

2.2 Unit definition volume

Name Predefined unit volume

Definition l

2.3 Unit definition substance

Name Predefined unit substance

Definition mol

2.4 Unit definition mole_per_second

Name mole per second

Definition $\mathrm{mol} \cdot \mathrm{s}^{-1}$

2.5 Unit definition per_second

Name per second

Definition s^{-1}

3 Compartments

This model contains five Compartments.

Table 2: Properties of each Compartment.

ID	Name	SBO	Spatial dimensions	Size	Unit	Constant	Outside
default	default	0000410	3	1	litre	☑	
cytoplasm	cytoplasm		3	1	litre	☑	
mitochondria	mitochondria		3	1	litre	☑	
transmembrane	transmembrane		3	1	litre	☑	
extracellular_region	extracellular region		3	1	litre	☑	

3.1 Compartment default

This is an three-dimensional compartment with a constant size of one l.

Name default

SBO:0000410 implicit compartment

3.2 Compartment cytoplasm

This is an three-dimensional compartment with a constant size of one l.

Name cytoplasm

3.3 Compartment mitochondria

This is an three-dimensional compartment with a constant size of one l.

Name mitochondria

3.4 Compartment transmembrane

This is an three-dimensional compartment with a constant size of one l.

Name transmembrane

3.5 Compartment extracellular_region

This is an three-dimensional compartment with a constant size of one l.

Name extracellular region

4 Species

This model contains 93 Species. Section ?? provides further details and the derived rates of change of each species.

Table 3: Properties of each Species.

ID	Name	Compartment	Derived unit	Constant	Boundary condition
RAX	RAX	default	$mol \cdot l^{-1}$	⊟	⊟
PKR	PKR	default	$mol \cdot l^{-1}$	⊟	⊟
ceramide	ceramide	default	$mol \cdot l^{-1}$	⊟	⊟
ERK1	ERK1	cytoplasm	$mol \cdot l^{-1}$	⊟	⊟
PKC_delta	PKC_delta	default	$mol \cdot l^{-1}$	⊟	⊟
KSR	KSR	default	$mol \cdot l^{-1}$	⊟	⊟
RAF1	RAF1	default	$mol \cdot l^{-1}$	⊟	⊟
BCL2	BCL2	default	$mol \cdot l^{-1}$	⊟	⊟
Cytochrome_C	Cytochrome_C	mitochondria	$mol \cdot l^{-1}$	⊟	⊟
Cytochrome_C_1	Cytochrome_C	cytoplasm	$mol \cdot l^{-1}$	⊟	⊟
Neutral-_sphingomyelinase-_II	Neutral_sphingomyelinase_II	default	$mol \cdot l^{-1}$	⊟	⊟
sphingomyelin	sphingomyelin	default	$mol \cdot l^{-1}$	⊟	⊟
PHOSPHOCHOLINE	PHOSPHOCHOLINE	default	$mol \cdot l^{-1}$	⊟	⊟
EIF2A	EIF2A	default	$mol \cdot l^{-1}$	⊟	⊟
PAR4	PAR4	default	$mol \cdot l^{-1}$	⊟	⊟
PKC_zeta	PKC_zeta	default	$mol \cdot l^{-1}$	⊟	⊟
response_to-_oxidative_stress	response to oxidative stress	default	$mol \cdot l^{-1}$	⊟	⊟
NF_kappa_B1	NF_kappa_B1	default	$mol \cdot l^{-1}$	⊟	⊟
RELA	RELA	default	$mol \cdot l^{-1}$	⊟	⊟

Produced by SBML2LaTeX version 1.0 for JSBML

Produced by SBML2LaTeX version 1.0 for JSBML

ID	Name	Compartment	Derived unit	Constant	Boundary condition
regulation_of_nitric_oxide_biosynthetic_process	regulation_of_nitric_oxide_biosynthetic_process	default	$mol \cdot l^{-1}$	⊟	⊟
Caspase_8	Caspase_8	cytoplasm	$mol \cdot l^{-1}$	⊟	⊟
AIF	AIF	mitochondria	$mol \cdot l^{-1}$	⊟	⊟
AIF_1	AIF	cytoplasm	$mol \cdot l^{-1}$	⊟	⊟
cell_survival	cell_survival	default	$mol \cdot l^{-1}$	⊟	⊟
cell_proliferation	cell_proliferation	default	$mol \cdot l^{-1}$	⊟	⊟
BAX	BAX	default	$mol \cdot l^{-1}$	⊟	⊟
SPHK2	SPHK2	default	$mol \cdot l^{-1}$	⊟	⊟
sphingosine	sphingosine	default	$mol \cdot l^{-1}$	⊟	⊟
Sphingosine1phosphate	Sphingosine-1-phosphate	default	$mol \cdot l^{-1}$	⊟	⊟
GD3	GD3	default	$mol \cdot l^{-1}$	⊟	⊟
Cathepsin_D	Cathepsin_D	default	$mol \cdot l^{-1}$	⊟	⊟
BID	BID	cytoplasm	$mol \cdot l^{-1}$	⊟	⊟
MAP4K4	MAP4K4	default	$mol \cdot l^{-1}$	⊟	⊟
MEKK1	MEKK1	default	$mol \cdot l^{-1}$	⊟	⊟
IKKs	IKKs	default	$mol \cdot l^{-1}$	⊟	⊟
I_kappa_B_alpha	I_kappa_B_alpha	default	$mol \cdot l^{-1}$	⊟	⊟
MKK4	MKK4	default	$mol \cdot l^{-1}$	⊟	⊟
JNK1	JNK1	cytoplasm	$mol \cdot l^{-1}$	⊟	⊟
JNK1_1	JNK1	default	$mol \cdot l^{-1}$	⊟	⊟
cIAP2	cIAP2	cytoplasm	$mol \cdot l^{-1}$	⊟	⊟
RAIDD	RAIDD	default	$mol \cdot l^{-1}$	⊟	⊟
TNFalpha	TNF-alpha	default	$mol \cdot l^{-1}$	⊟	⊟
TNFR1A	TNFR1A	transmembrane	$mol \cdot l^{-1}$	⊟	⊟
RIP	RIP	cytoplasm	$mol \cdot l^{-1}$	⊟	⊟

ID	Name	Compartment	Derived unit	Constant	Boundary condition
TRADD	TRADD	default	$mol \cdot l^{-1}$	⊟	⊟
MADD	MADD	default	$mol \cdot l^{-1}$	⊟	⊟
TRAF2	TRAF2	cytoplasm	$mol \cdot l^{-1}$	⊟	⊟
response_to_hydrogen_peroxide	response to hydrogen peroxide	default	$mol \cdot l^{-1}$	⊟	⊟
Acid_Sphingomyelinase	Acid_Sphingomyelinase	default	$mol \cdot l^{-1}$	⊟	⊟
MKK4_1	MKK4	cytoplasm	$mol \cdot l^{-1}$	⊟	⊟
EGF	EGF	extracellular_region	$mol \cdot l^{-1}$	⊟	⊟
negative_regulation_of_cell_cycle	negative_regulation_of_cell_cycle	default	$mol \cdot l^{-1}$	⊟	⊟
IGF1	IGF1	default	$mol \cdot l^{-1}$	⊟	⊟
tumor_necrosis_factor_receptor_activity	tumor necrosis factor receptor activity	default	$mol \cdot l^{-1}$	⊟	⊟
AKT1	AKT1	cytoplasm	$mol \cdot l^{-1}$	⊟	⊟
response_to_UV	response to UV	default	$mol \cdot l^{-1}$	⊟	⊟
PDGFA	PDGFA	default	$mol \cdot l^{-1}$	⊟	⊟
MYC	MYC	default	$mol \cdot l^{-1}$	⊟	⊟
glutathione	glutathione	default	$mol \cdot l^{-1}$	⊟	⊟
FAN	FAN	default	$mol \cdot l^{-1}$	⊟	⊟
TNFR1A_1	TNFR1A	default	$mol \cdot l^{-1}$	⊟	⊟
GW4869	GW4869	default	$mol \cdot l^{-1}$	⊟	⊟
C11AG	C11AG	default	$mol \cdot l^{-1}$	⊟	⊟
TNFalpha_1	TNF-alpha	extracellular_region	$mol \cdot l^{-1}$	⊟	⊟
MEK2	MEK2	default	$mol \cdot l^{-1}$	⊟	⊟
ERK2	ERK2	cytoplasm	$mol \cdot l^{-1}$	⊟	⊟
MEK1	MEK1	default	$mol \cdot l^{-1}$	⊟	⊟

Produced by SBML2LaTeX version 1.0 for JSBML

ID	Name	Compartment	Derived unit	Constant	Boundary condition
BAD	BAD	default	$mol \cdot l^{-1}$	⊟	⊟
Acid_Ceramidase	Acid_Ceramidase	default	$mol \cdot l^{-1}$	⊟	⊟
Free_Fatty_acid	Free_Fatty_acid	default	$mol \cdot l^{-1}$	⊟	⊟
response_to-_radiation	response to radiation	default	$mol \cdot l^{-1}$	⊟	⊟
MEK1_1	MEK1	cytoplasm	$mol \cdot l^{-1}$	⊟	⊟
MEK2_1	MEK2	cytoplasm	$mol \cdot l^{-1}$	⊟	⊟
FADD	FADD	default	$mol \cdot l^{-1}$	⊟	⊟
RB1	RB1	default	$mol \cdot l^{-1}$	⊟	⊟
response_to_heat	response to heat	default	$mol \cdot l^{-1}$	⊟	⊟
BAG4	BAG4	cytoplasm	$mol \cdot l^{-1}$	⊟	⊟
I_kappa_B_alpha-_degradation_00	I_kappa_B_alpha_(degradation)_0-0	default	$mol \cdot l^{-1}$	⊟	⊟
OKADAIC_ACID	OKADAIC_ACID	default	$mol \cdot l^{-1}$	⊟	⊟
negative-_transcription-_elongation-_factor_activity	negative transcription elongation factor activity	default	$mol \cdot l^{-1}$	⊟	⊟
ERK1PKC_delta	ERK1/PKC_delta	default	$mol \cdot l^{-1}$	⊟	⊟
PKC_zetaPAR4	PKC_zeta/PAR4	default	$mol \cdot l^{-1}$	⊟	⊟
PKC_zetaceramide	PKC_zeta/ceramide	default	$mol \cdot l^{-1}$	⊟	⊟
RelANF_kappa_B1	RelA/NF_kappa_B1	cytoplasm	$mol \cdot l^{-1}$	⊟	⊟
Cathepsin-_Dceramide	Cathepsin_D/ceramide	default	$mol \cdot l^{-1}$	⊟	⊟
NF_kappa_B1RelAI-_kappa_B_alpha	NF_kappa_B1/RelA/I_kappa_B_alpha	cytoplasm	$mol \cdot l^{-1}$	⊟	⊟
NF_kappa_B1RelAI-_kappa_B_alpha_1	NF_kappa_B1/RelA/I_kappa_B_alpha	cytoplasm	$mol \cdot l^{-1}$	⊟	⊟
TNFalphaTNFR1ATRADDMADDcIAP2RIPTRAF2RAIDD	TNF-alpha/TNFR1A/TRADD/MADD/cIAP2/RIP/TRAF2/RAIDD	default	$mol \cdot l^{-1}$	⊟	⊟

Produced by SBML2LaTeX version 1.0 for JSBML

ID	Name	Compartment	Derived unit	Constant	Boundary condition
PP2A_Heterotrimer	PP2A_Heterotrimer	mitochondria	$mol \cdot l^{-1}$	⊟	⊟
TNFalphaTNFR1AFAN	TNF-alpha/TNFR1A/FAN	default	$mol \cdot l^{-1}$	⊟	⊟
FADDCaspase_8	FADD/Caspase_8	default	$mol \cdot l^{-1}$	⊟	⊟
TNFR1ABAG4	TNFR1A/BAG4	default	$mol \cdot l^{-1}$	⊟	⊟
TNFR1ABAG4TNFalpha	TNFR1A/BAG4/TNF-alpha	default	$mol \cdot l^{-1}$	⊟	⊟

5 Parameters

This model contains 263 global Parameters.

Table 4: Properties of each Parameter.

ID	Name	SBO	Value	Unit	Constant
vmar_unknown1_2	Revere maximal velocity of reaction unknown1_2	0000325	1.0	$\text{mol} \cdot \text{s}^{-1}$	☑
kmc_unknown1_2_PKR	Michaelis constant of species PKR in reaction unknown1_2	0000027	1.0	mol	☑
hco_unknown1_2	Reaction cooperativity	0000382	1.0	dimensionless	☑
vmaf_unknown1_2	Forward maximal velocity of reaction unknown1_2	0000324	1.0	$\text{mol} \cdot \text{s}^{-1}$	☑
kmc_unknown2_ERK1PKC_delta	Michaelis constant of species ERK1/PKC_delta in reaction unknown2	0000027	1.0	mol	☑
vmar_unknown2	Revere maximal velocity of reaction unknown2	0000325	1.0	$\text{mol} \cdot \text{s}^{-1}$	☑
kmc_unknown2_PKC_delta	Michaelis constant of species PKC_delta in reaction unknown2	0000027	1.0	mol	☑
kmc_unknown2_ERK1	Michaelis constant of species ERK1 in reaction unknown2	0000027	1.0	mol	☑
hco_unknown2	Reaction cooperativity	0000382	1.0	dimensionless	☑
vmaf_unknown2	Forward maximal velocity of reaction unknown2	0000324	1.0	$\text{mol} \cdot \text{s}^{-1}$	☑

ID	Name	SBO	Value	Unit	Constant
vmar-_unknown3	Revere maximal velocity of reaction unknown3	0000325	1.0	$\mathrm{mol \cdot s^{-1}}$	☑
kmc-_unknown3_KSR	Michaelis constant of species KSR in reaction unknown3	0000027	1.0	mol	☑
hco_unknown3	Reaction cooperativity	0000382	1.0	dimensionless	☑
vmaf-_unknown3	Forward maximal velocity of reaction unknown3	0000324	1.0	$\mathrm{mol \cdot s^{-1}}$	☑
vmar-_unknown4_1	Revere maximal velocity of reaction unknown4_1	0000325	1.0	$\mathrm{mol \cdot s^{-1}}$	☑
kmc-_unknown4_1-_RAF1	Michaelis constant of species RAF1 in reaction unknown4_1	0000027	1.0	mol	☑
hco-_unknown4_1	Reaction cooperativity	0000382	1.0	dimensionless	☑
vmaf-_unknown4_1	Forward maximal velocity of reaction unknown4_1	0000324	1.0	$\mathrm{mol \cdot s^{-1}}$	☑
kmc-_unknown5_1-_Cytochrome-_C_1	Michaelis constant of species Cytochrome-_C in reaction unknown5_1	0000027	1.0	mol	☑
vmar-_unknown5_1	Revere maximal velocity of reaction unknown5_1	0000325	1.0	$\mathrm{mol \cdot s^{-1}}$	☑
kmc-_unknown5_1-_Cytochrome_C	Michaelis constant of species Cytochrome-_C in reaction unknown5_1	0000027	1.0	mol	☑
hco-_unknown5_1	Reaction cooperativity	0000382	1.0	dimensionless	☑

ID	Name	SBO	Value	Unit	Constant
vmaf-_unknown5_1	Forward maximal velocity of reaction unknown5_1	0000324	1.0	$mol \cdot s^{-1}$	☑
kmc-_unknown6-_PHOSPHOCHOLINE	Michaelis constant of species PHOSPHOCHOLINE in reaction unknown6	0000027	1.0	mol	☑
kmc-_unknown6-_ceramide	Michaelis constant of species ceramide in reaction unknown6	0000027	1.0	mol	☑
vmar-_unknown6	Revere maximal velocity of reaction unknown6	0000325	1.0	$mol \cdot s^{-1}$	☑
kmc-_unknown6-_sphingomyelin	Michaelis constant of species sphingomyelin in reaction unknown6	0000027	1.0	mol	☑
hco_unknown6	Reaction cooperativity	0000382	1.0	dimensionless	☑
vmaf-_unknown6	Forward maximal velocity of reaction unknown6	0000324	1.0	$mol \cdot s^{-1}$	☑
vmar-_unknown7	Revere maximal velocity of reaction unknown7	0000325	1.0	$mol \cdot s^{-1}$	☑
kmc-_unknown7-_EIF2A	Michaelis constant of species EIF2A in reaction unknown7	0000027	1.0	mol	☑
hco_unknown7	Reaction cooperativity	0000382	1.0	dimensionless	☑
vmaf-_unknown7	Forward maximal velocity of reaction unknown7	0000324	1.0	$mol \cdot s^{-1}$	☑
kmc-_unknown8-_1_PKC-_zetaceramide	Michaelis constant of species PKC-_zeta/ceramide in reaction unknown8_1	0000027	1.0	mol	☑

ID	Name	SBO	Value	Unit	Constant
kmc-_unknown8_1-_PKC_zetaPAR4	Michaelis constant of species PKC-_zeta/PAR4 in reaction unknown8_1	0000027	1.0	mol	☑
vmar-_unknown8_1	Revere maximal velocity of reaction unknown8_1	0000325	1.0	$\mathrm{mol} \cdot \mathrm{s}^{-1}$	☑
kmc-_unknown8_1-_PKC_zeta	Michaelis constant of species PKC-_zeta in reaction unknown8_1	0000027	1.0	mol	☑
kmc-_unknown8_1-_ceramide	Michaelis constant of species ceramide in reaction unknown8_1	0000027	1.0	mol	☑
kmc-_unknown8_1-_PAR4	Michaelis constant of species PAR4 in reaction unknown8_1	0000027	1.0	mol	☑
hco-_unknown8_1	Reaction cooperativity	0000382	1.0	dimensionless	☑
vmaf-_unknown8_1	Forward maximal velocity of reaction unknown8_1	0000324	1.0	$\mathrm{mol} \cdot \mathrm{s}^{-1}$	☑
kcrr-_unknown9-_TNFalphaTNFR1AFAN	Product catalytic rate constant of enzyme TNF-alpha/TNFR1A/FAN in reaction unknown9	0000321	1.0	s^{-1}	☑
kmc-_unknown9-_Neutral-_sphingomyelinase-_II-_TNFalphaTNFR1AFAN	Michaelis constant of species Neutral-_sphingomyelinase-II and enzyme TNF-alpha/TNFR1A/FAN in reaction unknown9	0000027	1.0	mol	☑

ID	Name	SBO	Value	Unit	Constant
hco-_unknown9-_TNFalphaTNFR1AFAN	Reaction cooperativity	0000382	1.0	dimensionless	☑
kcrf-_unknown9-_TNFalphaTNFR1AFAN	Substrate catalytic rate constant of enzyme TNF-alpha/TNFR1A/FAN in reaction unknown9	0000320	1.0	s^{-1}	☑
kcrr-_unknown9-_TNFalphaTNFR1ATRADDMADDcIAP2RIPTRAF2RAIDD	Product catalytic rate constant of enzyme TNF-alpha/TNFR1A/TRADD/MADD/cIAP2/RIP/TRAF2/RAIDD in reaction unknown9	0000321	1.0	s^{-1}	☑
kmc-_unknown9-_Neutral-_sphingomyelinase-_II-_TNFalphaTNFR1ATRADDMADDcIAP2RIPTRAF2RAIDD	Michaelis constant of species Neutral-_sphingomyelinase-II and enzyme TNF-alpha/TNFR1A/TRADD/MADD/cIAP2/RIP/TRAF2/RAIDD in reaction unknown9	0000027	1.0	mol	☑
hco-_unknown9-_TNFalphaTNFR1ATRADDMADDcIAP2RIPTRAF2RAIDD	Reaction cooperativity	0000382	1.0	dimensionless	☑
kcrf-_unknown9-_TNFalphaTNFR1ATRADDMADDcIAP2RIPTRAF2RAIDD	Substrate catalytic rate constant of enzyme TNF-alpha/TNFR1A/TRADD/MADD/cIAP2/RIP/TRAF2/RAIDD in reaction unknown9	0000320	1.0	s^{-1}	☑
kcrr-_unknown10-_2_RelANF-_kappa_B1	Product catalytic rate constant of enzyme RelA/NF-_kappa_B1 in reaction unknown10_2	0000321	1.0	s^{-1}	☑

ID	Name	SBO	Value	Unit	Constant
kmc-_unknown10-_2_Caspase-_8_RelANF-_kappa_B1	Michaelis constant of species Caspase-_8 and enzyme RelA/NF_kappa-_B1 in reaction unknown10_2	0000027	1.0	mol	☑
hco-_unknown10-_2_RelANF-_kappa_B1	Reaction cooperativity	0000382	1.0	dimensionless	☑
kcrf-_unknown10-_2_RelANF-_kappa_B1	Substrate catalytic rate constant of enzyme RelA/NF-_kappa_B1 in reaction unknown10_2	0000320	1.0	s^{-1}	☑
kmc-_unknown11-_AIF_1	Michaelis constant of species AIF in reaction unknown11	0000027	1.0	mol	☑
vmar-_unknown11	Revere maximal velocity of reaction unknown11	0000325	1.0	$mol \cdot s^{-1}$	☑
kmc-_unknown11-_AIF	Michaelis constant of species AIF in reaction unknown11	0000027	1.0	mol	☑
hco-_unknown11	Reaction cooperativity	0000382	1.0	dimensionless	☑
vmaf-_unknown11	Forward maximal velocity of reaction unknown11	0000324	1.0	$mol \cdot s^{-1}$	☑
vmar-_unknown12	Revere maximal velocity of reaction unknown12	0000325	1.0	$mol \cdot s^{-1}$	☑
kmc-_unknown12-_BAX	Michaelis constant of species BAX in reaction unknown12	0000027	1.0	mol	☑

ID	Name	SBO	Value	Unit	Constant
hco-_unknown12	Reaction cooperativity	0000382	1.0	dimensionless	☑
vmaf-_unknown12	Forward maximal velocity of reaction unknown12	0000324	1.0	$mol \cdot s^{-1}$	☑
kmc-_unknown13_1-_Sphingosine1phosphate	Michaelis constant of species Sphingosine-1-phosphate in reaction unknown13_1	0000027	1.0	mol	☑
vmar-_unknown13_1	Revere maximal velocity of reaction unknown13_1	0000325	1.0	$mol \cdot s^{-1}$	☑
kmc-_unknown13_1-_sphingosine	Michaelis constant of species sphingosine in reaction unknown13_1	0000027	1.0	mol	☑
hco-_unknown13_1	Reaction cooperativity	0000382	1.0	dimensionless	☑
vmaf-_unknown13_1	Forward maximal velocity of reaction unknown13_1	0000324	1.0	$mol \cdot s^{-1}$	☑
kcrr-_unknown14_1-_Cathepsin-_Dceramide	Product catalytic rate constant of enzyme Cathepsin-_D/ceramide in reaction unknown14-_1	0000321	1.0	s^{-1}	☑
kmc-_unknown14-_1_BID-_Cathepsin-_Dceramide	Michaelis constant of species BID and enzyme Cathepsin-_D/ceramide in reaction unknown14-_1	0000027	1.0	mol	☑
hco-_unknown14_1-_Cathepsin-_Dceramide	Reaction cooperativity	0000382	1.0	dimensionless	☑

ID	Name	SBO	Value	Unit	Constant
kcrf-_unknown14_1-_Cathepsin-_Dceramide	Substrate catalytic rate constant of enzyme Cathepsin-_D/ceramide in reaction unknown14-_1	0000320	1.0	s^{-1}	☑
kcrr-_unknown14_1-_TNFalphaTNFR1ATRADDMADDcIAP2RIPTRAF2RAIDD	Product catalytic rate constant of enzyme TNF-alpha/TNFR1A/TRADD/MADD/cIAP2/RIP/TRAF2/RAIDD in reaction unknown14_1	0000321	1.0	s^{-1}	☑
kmc-_unknown14-_1_BID-_TNFalphaTNFR1ATRADDMADDcIAP2RIPTRAF2RAIDD	Michaelis constant of species BID and enzyme TNF-alpha/TNFR1A/TRADD/MADD/cIAP2/RIP/TRAF2/RAIDD in reaction unknown14_1	0000027	1.0	mol	☑
hco-_unknown14_1-_TNFalphaTNFR1ATRADDMADDcIAP2RIPTRAF2RAIDD	Reaction cooperativity	0000382	1.0	dimensionless	☑
kcrf-_unknown14_1-_TNFalphaTNFR1ATRADDMADDcIAP2RIPTRAF2RAIDD	Substrate catalytic rate constant of enzyme TNF-alpha/TNFR1A/TRADD/MADD/cIAP2/RIP/TRAF2/RAIDD in reaction unknown14_1	0000320	1.0	s^{-1}	☑
kcrr-_unknown15_2-_TNFalphaTNFR1ATRADDMADDcIAP2RIPTRAF2RAIDD	Product catalytic rate constant of enzyme TNF-alpha/TNFR1A/TRADD/MADD/cIAP2/RIP/TRAF2/RAIDD in reaction unknown15_2	0000321	1.0	s^{-1}	☑

ID	Name	SBO	Value	Unit	Constant
kmc_unknown15_2_MAP4K4_TNFalphaTNFR1ATRADDMADDcIAP2RIPTRAF2RAIDD	Michaelis constant of species MAP4K4 and enzyme TNF-alpha/TNFR1A/TRADD/MADD/cIAP2/RIP/TRAF2/RAIDD in reaction unknown15_2	0000027	1.0	mol	☑
hco_unknown15_2_TNFalphaTNFR1ATRADDMADDcIAP2RIPTRAF2RAIDD	Reaction cooperativity	0000382	1.0	dimensionless	☑
kcrf_unknown15_2_TNFalphaTNFR1ATRADDMADDcIAP2RIPTRAF2RAIDD	Substrate catalytic rate constant of enzyme TNF-alpha/TNFR1A/TRADD/MADD/cIAP2/RIP/TRAF2/RAIDD in reaction unknown15_2	0000320	1.0	s^{-1}	☑
vmar_unknown16_1	Revere maximal velocity of reaction unknown16_1	0000325	1.0	$mol \cdot s^{-1}$	☑
kmc_unknown16_1_MEKK1	Michaelis constant of species MEKK1 in reaction unknown16_1	0000027	1.0	mol	☑
hco_unknown16_1	Reaction cooperativity	0000382	1.0	dimensionless	☑
vmaf_unknown16_1	Forward maximal velocity of reaction unknown16_1	0000324	1.0	$mol \cdot s^{-1}$	☑
vmar_unknown17	Revere maximal velocity of reaction unknown17	0000325	1.0	$mol \cdot s^{-1}$	☑
kmc_unknown17_IKKs	Michaelis constant of species IKKs in reaction unknown17	0000027	1.0	mol	☑
hco_unknown17	Reaction cooperativity	0000382	1.0	dimensionless	☑

ID	Name	SBO	Value	Unit	Constant
vmaf-_unknown17	Forward maximal velocity of reaction unknown17	0000324	1.0	$mol \cdot s^{-1}$	☑
kmc-_unknown18-_1_NF_kappa-_B1RelAI-_kappa_B-_alpha_1_PKC-_zetaPAR4	Michaelis constant of species NF-_kappa_B1/RelA/I-_kappa_B_alpha and enzyme PKC-_zeta/PAR4 in reaction unknown18_1	0000027	1.0	mol	☑
kcrr-_unknown18_1-_PKC_zetaPAR4	Product catalytic rate constant of enzyme PKC-_zeta/PAR4 in reaction unknown18_1	0000321	1.0	s^{-1}	☑
kmc-_unknown18-_1_NF_kappa-_B1RelAI-_kappa_B-_alpha_PKC-_zetaPAR4	Michaelis constant of species NF-_kappa_B1/RelA/I-_kappa_B_alpha and enzyme PKC-_zeta/PAR4 in reaction unknown18_1	0000027	1.0	mol	☑
hco-_unknown18_1-_PKC_zetaPAR4	Reaction cooperativity	0000382	1.0	dimensionless	☑
kcrf-_unknown18_1-_PKC_zetaPAR4	Substrate catalytic rate constant of enzyme PKC-_zeta/PAR4 in reaction unknown18_1	0000320	1.0	s^{-1}	☑
kmc-_unknown19-_JNK1_1_PKC-_zetaceramide	Michaelis constant of species JNK1 and enzyme PKC-_zeta/ceramide in reaction unknown19	0000027	1.0	mol	☑

ID	Name	SBO	Value	Unit	Constant
kcrr-_unknown19-_PKC-_zetaceramide	Product catalytic rate constant of enzyme PKC-_zeta/ceramide in reaction unknown19	0000321	1.0	s^{-1}	☑
kmc-_unknown19-_JNK1_PKC-_zetaceramide	Michaelis constant of species JNK1 and enzyme PKC-_zeta/ceramide in reaction unknown19	0000027	1.0	mol	☑
hco-_unknown19-_PKC-_zetaceramide	Reaction cooperativity	0000382	1.0	dimensionless	☑
kcrf-_unknown19-_PKC-_zetaceramide	Substrate catalytic rate constant of enzyme PKC-_zeta/ceramide in reaction unknown19	0000320	1.0	s^{-1}	☑
kcrr-_unknown20-_TNFalphaTNFR1ATRADDMADDcIAP2RIPTRAF2RAIDD	Product catalytic rate constant of enzyme TNF-alpha/TNFR1A/TRADD/MADD/cIAP2/RIP/TRAF2/RAIDD in reaction unknown20	0000321	1.0	s^{-1}	☑
kmc-_unknown20-_Acid-_Sphingomyelinase-_TNFalphaTNFR1ATRADDMADDcIAP2RIPTRAF2RAIDD	Michaelis constant of species Acid-_Sphingomyelinase and enzyme TNF-alpha/TNFR1A/TRADD/MADD/cIAP2/RIP/TRAF2/RAIDD in reaction unknown20	0000027	1.0	mol	☑
hco-_unknown20-_TNFalphaTNFR1ATRADDMADDcIAP2RIPTRAF2RAIDD	Reaction cooperativity	0000382	1.0	dimensionless	☑

ID	Name	SBO	Value	Unit	Constant
kcrf_unknown20_TNFalphaTNFR1ATRADDMADDcIAP2RIPTRAF2RAIDD	Substrate catalytic rate constant of enzyme TNF-alpha/TNFR1A/TRADD/MADD/cIAP2/RIP/TRAF2/RAIDD in reaction unknown20	0000320	1.0	s^{-1}	☑
kcrr_unknown20_TNFalphaTNFR1AFAN	Product catalytic rate constant of enzyme TNF-alpha/TNFR1A/FAN in reaction unknown20	0000321	1.0	s^{-1}	☑
kmc_unknown20_Acid_Sphingomyelinase_TNFalphaTNFR1AFAN	Michaelis constant of species Acid_Sphingomyelinase and enzyme TNF-alpha/TNFR1A/FAN in reaction unknown20	0000027	1.0	mol	☑
hco_unknown20_TNFalphaTNFR1AFAN	Reaction cooperativity	0000382	1.0	dimensionless	☑
kcrf_unknown20_TNFalphaTNFR1AFAN	Substrate catalytic rate constant of enzyme TNF-alpha/TNFR1A/FAN in reaction unknown20	0000320	1.0	s^{-1}	☑
kmc_unknown21_MKK4	Michaelis constant of species MKK4 in reaction unknown21	0000027	1.0	mol	☑
vmar_unknown21	Revere maximal velocity of reaction unknown21	0000325	1.0	$mol \cdot s^{-1}$	☑
kmc_unknown21_MKK4_1	Michaelis constant of species MKK4 in reaction unknown21	0000027	1.0	mol	☑

ID	Name	SBO	Value	Unit	Constant
hco-_unknown21	Reaction cooperativity	0000382	1.0	dimensionless	☑
vmaf-_unknown21	Forward maximal velocity of reaction unknown21	0000324	1.0	$mol \cdot s^{-1}$	☑
kmc-_unknown22-_Cathepsin-_Dceramide	Michaelis constant of species Cathepsin-_D/ceramide in reaction unknown22	0000027	1.0	mol	☑
vmar-_unknown22	Revere maximal velocity of reaction unknown22	0000325	1.0	$mol \cdot s^{-1}$	☑
kmc-_unknown22-_ceramide	Michaelis constant of species ceramide in reaction unknown22	0000027	1.0	mol	☑
kmc-_unknown22-_Cathepsin_D	Michaelis constant of species Cathepsin_D in reaction unknown22	0000027	1.0	mol	☑
hco-_unknown22	Reaction cooperativity	0000382	1.0	dimensionless	☑
vmaf-_unknown22	Forward maximal velocity of reaction unknown22	0000324	1.0	$mol \cdot s^{-1}$	☑
kmc-_unknown23-_ceramide	Michaelis constant of species ceramide in reaction unknown23	0000027	1.0	mol	☑
vmar-_unknown23	Revere maximal velocity of reaction unknown23	0000325	1.0	$mol \cdot s^{-1}$	☑
kmc-_unknown23-_sphingomyelin	Michaelis constant of species sphingomyelin in reaction unknown23	0000027	1.0	mol	☑

ID	Name	SBO	Value	Unit	Constant
hco-_unknown23	Reaction cooperativity	0000382	1.0	dimensionless	☑
vmaf-_unknown23	Forward maximal velocity of reaction unknown23	0000324	1.0	$\text{mol} \cdot \text{s}^{-1}$	☑
vmar-_unknown24	Revere maximal velocity of reaction unknown24	0000325	1.0	$\text{mol} \cdot \text{s}^{-1}$	☑
kmc-_unknown24-_PP2A-_Heterotrimer	Michaelis constant of species PP2A-_Heterotrimer in reaction unknown24	0000027	1.0	mol	☑
hco-_unknown24	Reaction cooperativity	0000382	1.0	dimensionless	☑
vmaf-_unknown24	Forward maximal velocity of reaction unknown24	0000324	1.0	$\text{mol} \cdot \text{s}^{-1}$	☑
kcrr-_unknown25-_1_PKC-_zetaceramide	Product catalytic rate constant of enzyme PKC-_zeta/ceramide in reaction unknown25_1	0000321	1.0	s^{-1}	☑
kmc-_unknown25-_1_AKT1_PKC-_zetaceramide	Michaelis constant of species AKT1 and enzyme PKC-_zeta/ceramide in reaction unknown25_1	0000027	1.0	mol	☑
hco-_unknown25-_1_PKC-_zetaceramide	Reaction cooperativity	0000382	1.0	dimensionless	☑
kcrf-_unknown25-_1_PKC-_zetaceramide	Substrate catalytic rate constant of enzyme PKC-_zeta/ceramide in reaction unknown25_1	0000320	1.0	s^{-1}	☑

ID	Name	SBO	Value	Unit	Constant
vmar-_unknown26	Revere maximal velocity of reaction unknown26	0000325	1.0	$\mathrm{mol} \cdot \mathrm{s}^{-1}$	☑
kmc-_unknown26-_SPHK2	Michaelis constant of species SPHK2 in reaction unknown26	0000027	1.0	mol	☑
hco-_unknown26	Reaction cooperativity	0000382	1.0	dimensionless	☑
vmaf-_unknown26	Forward maximal velocity of reaction unknown26	0000324	1.0	$\mathrm{mol} \cdot \mathrm{s}^{-1}$	☑
vmar-_unknown28	Revere maximal velocity of reaction unknown28	0000325	1.0	$\mathrm{mol} \cdot \mathrm{s}^{-1}$	☑
kmc-_unknown28-_PP2A-_Heterotrimer	Michaelis constant of species PP2A-_Heterotrimer in reaction unknown28	0000027	1.0	mol	☑
hco-_unknown28	Reaction cooperativity	0000382	1.0	dimensionless	☑
vmaf-_unknown28	Forward maximal velocity of reaction unknown28	0000324	1.0	$\mathrm{mol} \cdot \mathrm{s}^{-1}$	☑
vmar-_unknown30_1	Revere maximal velocity of reaction unknown30_1	0000325	1.0	$\mathrm{mol} \cdot \mathrm{s}^{-1}$	☑
kmc-_unknown30_1-_RAX	Michaelis constant of species RAX in reaction unknown30_1	0000027	1.0	mol	☑
hco-_unknown30_1	Reaction cooperativity	0000382	1.0	dimensionless	☑
vmaf-_unknown30_1	Forward maximal velocity of reaction unknown30_1	0000324	1.0	$\mathrm{mol} \cdot \mathrm{s}^{-1}$	☑
vmar-_unknown31	Revere maximal velocity of reaction unknown31	0000325	1.0	$\mathrm{mol} \cdot \mathrm{s}^{-1}$	☑

ID	Name	SBO	Value	Unit	Constant
kmc-_unknown31-_AKT1	Michaelis constant of species AKT1 in reaction unknown31	0000027	1.0	mol	☑
hco-_unknown31	Reaction cooperativity	0000382	1.0	dimensionless	☑
vmaf-_unknown31	Forward maximal velocity of reaction unknown31	0000324	1.0	$\mathrm{mol} \cdot \mathrm{s}^{-1}$	☑
kmc-_unknown32-_TNFalphaTNFR1AFAN	Michaelis constant of species TNF-alpha/TNFR1A/FAN in reaction unknown32	0000027	1.0	mol	☑
vmar-_unknown32	Revere maximal velocity of reaction unknown32	0000325	1.0	$\mathrm{mol} \cdot \mathrm{s}^{-1}$	☑
kmc-_unknown32-_FAN	Michaelis constant of species FAN in reaction unknown32	0000027	1.0	mol	☑
kmc-_unknown32-_TNFalpha_1	Michaelis constant of species TNF-alpha in reaction unknown32	0000027	1.0	mol	☑
kmc-_unknown32-_TNFR1A_1	Michaelis constant of species TNFR1A in reaction unknown32	0000027	1.0	mol	☑
hco-_unknown32	Reaction cooperativity	0000382	1.0	dimensionless	☑
vmaf-_unknown32	Forward maximal velocity of reaction unknown32	0000324	1.0	$\mathrm{mol} \cdot \mathrm{s}^{-1}$	☑
vmar-_unknown33_1	Revere maximal velocity of reaction unknown33_1	0000325	1.0	$\mathrm{mol} \cdot \mathrm{s}^{-1}$	☑

ID	Name	SBO	Value	Unit	Constant
kmc-_unknown33_1-_ERK1	Michaelis constant of species ERK1 in reaction unknown33_1	0000027	1.0	mol	☑
kmc-_unknown33_1-_ERK2	Michaelis constant of species ERK2 in reaction unknown33_1	0000027	1.0	mol	☑
hco-_unknown33_1	Reaction cooperativity	0000382	1.0	dimensionless	☑
vmaf-_unknown33_1	Forward maximal velocity of reaction unknown33_1	0000324	1.0	$\mathrm{mol} \cdot \mathrm{s}^{-1}$	☑
vmar-_unknown34	Revere maximal velocity of reaction unknown34	0000325	1.0	$\mathrm{mol} \cdot \mathrm{s}^{-1}$	☑
kmc-_unknown34-_ERK1	Michaelis constant of species ERK1 in reaction unknown34	0000027	1.0	mol	☑
kmc-_unknown34-_ERK2	Michaelis constant of species ERK2 in reaction unknown34	0000027	1.0	mol	☑
hco-_unknown34	Reaction cooperativity	0000382	1.0	dimensionless	☑
vmaf-_unknown34	Forward maximal velocity of reaction unknown34	0000324	1.0	$\mathrm{mol} \cdot \mathrm{s}^{-1}$	☑
kcrr-_unknown35-_PP2A-_Heterotrimer	Product catalytic rate constant of enzyme PP2A-_Heterotrimer in reaction unknown35	0000321	1.0	s^{-1}	☑

ID	Name	SBO	Value	Unit	Constant
kmc-_unknown35-_BCL2_PP2A-_Heterotrimer	Michaelis constant of species BCL2 and enzyme PP2A-_Heterotrimer in reaction unknown35	0000027	1.0	mol	☑
hco-_unknown35-_PP2A-_Heterotrimer	Reaction cooperativity	0000382	1.0	dimensionless	☑
kcrf-_unknown35-_PP2A-_Heterotrimer	Substrate catalytic rate constant of enzyme PP2A-_Heterotrimer in reaction unknown35	0000320	1.0	s^{-1}	☑
vmar-_unknown36	Revere maximal velocity of reaction unknown36	0000325	1.0	$mol \cdot s^{-1}$	☑
kmc-_unknown36-_BAD	Michaelis constant of species BAD in reaction unknown36	0000027	1.0	mol	☑
hco-_unknown36	Reaction cooperativity	0000382	1.0	dimensionless	☑
vmaf-_unknown36	Forward maximal velocity of reaction unknown36	0000324	1.0	$mol \cdot s^{-1}$	☑
vmar-_unknown37	Revere maximal velocity of reaction unknown37	0000325	1.0	$mol \cdot s^{-1}$	☑
kmc-_unknown37-_BAD	Michaelis constant of species BAD in reaction unknown37	0000027	1.0	mol	☑
hco-_unknown37	Reaction cooperativity	0000382	1.0	dimensionless	☑
vmaf-_unknown37	Forward maximal velocity of reaction unknown37	0000324	1.0	$mol \cdot s^{-1}$	☑

ID	Name	SBO	Value	Unit	Constant
kmc_unknown38_Free_Fatty_acid	Michaelis constant of species Free_Fatty_acid in reaction unknown38	0000027	1.0	mol	☑
kmc_unknown38_sphingosine	Michaelis constant of species sphingosine in reaction unknown38	0000027	1.0	mol	☑
vmar_unknown38	Revere maximal velocity of reaction unknown38	0000325	1.0	$mol \cdot s^{-1}$	☑
kmc_unknown38_ceramide	Michaelis constant of species ceramide in reaction unknown38	0000027	1.0	mol	☑
hco_unknown38	Reaction cooperativity	0000382	1.0	dimensionless	☑
vmaf_unknown38	Forward maximal velocity of reaction unknown38	0000324	1.0	$mol \cdot s^{-1}$	☑
kmc_unknown39_Free_Fatty_acid	Michaelis constant of species Free_Fatty_acid in reaction unknown39	0000027	1.0	mol	☑
kmc_unknown39_sphingosine	Michaelis constant of species sphingosine in reaction unknown39	0000027	1.0	mol	☑
vmar_unknown39	Revere maximal velocity of reaction unknown39	0000325	1.0	$mol \cdot s^{-1}$	☑
kmc_unknown39_ceramide	Michaelis constant of species ceramide in reaction unknown39	0000027	1.0	mol	☑
hco_unknown39	Reaction cooperativity	0000382	1.0	dimensionless	☑

ID	Name	SBO	Value	Unit	Constant
vmaf-_unknown39	Forward maximal velocity of reaction unknown39	0000324	1.0	$\mathrm{mol} \cdot \mathrm{s}^{-1}$	☑
vmar-_unknown40	Revere maximal velocity of reaction unknown40	0000325	1.0	$\mathrm{mol} \cdot \mathrm{s}^{-1}$	☑
kmc-_unknown40-_ERK1	Michaelis constant of species ERK1 in reaction unknown40	0000027	1.0	mol	☑
kmc-_unknown40-_ERK2	Michaelis constant of species ERK2 in reaction unknown40	0000027	1.0	mol	☑
hco-_unknown40	Reaction cooperativity	0000382	1.0	dimensionless	☑
vmaf-_unknown40	Forward maximal velocity of reaction unknown40	0000324	1.0	$\mathrm{mol} \cdot \mathrm{s}^{-1}$	☑
kmc-_unknown41-_MEK1	Michaelis constant of species MEK1 in reaction unknown41	0000027	1.0	mol	☑
kmc-_unknown41-_MEK2	Michaelis constant of species MEK2 in reaction unknown41	0000027	1.0	mol	☑
vmar-_unknown41	Revere maximal velocity of reaction unknown41	0000325	1.0	$\mathrm{mol} \cdot \mathrm{s}^{-1}$	☑
kmc-_unknown41-_MEK2_1	Michaelis constant of species MEK2 in reaction unknown41	0000027	1.0	mol	☑
kmc-_unknown41-_MEK1_1	Michaelis constant of species MEK1 in reaction unknown41	0000027	1.0	mol	☑

ID	Name	SBO	Value	Unit	Constant
hco_unknown41	Reaction cooperativity	0000382	1.0	dimensionless	☑
vmaf_unknown41	Forward maximal velocity of reaction unknown41	0000324	1.0	$mol \cdot s^{-1}$	☑
kmc_unknown42_3_FADD_TNFalphaTNFR1ATRADDMADDcIAP2RIPTRAF2RAIDD	Michaelis constant of species FADD and enzyme TNF-alpha/TNFR1A/TRADD/MADD/cIAP2/RIP/TRAF2/RAIDD in reaction unknown42_3	0000027	1.0	mol	☑
kmc_unknown42_3_Caspase_8_TNFalphaTNFR1ATRADDMADDcIAP2RIPTRAF2RAIDD	Michaelis constant of species Caspase_8 and enzyme TNF-alpha/TNFR1A/TRADD/MADD/cIAP2/RIP/TRAF2/RAIDD in reaction unknown42_3	0000027	1.0	mol	☑
kcrr_unknown42_3_TNFalphaTNFR1ATRADDMADDcIAP2RIPTRAF2RAIDD	Product catalytic rate constant of enzyme TNF-alpha/TNFR1A/TRADD/MADD/cIAP2/RIP/TRAF2/RAIDD in reaction unknown42_3	0000321	1.0	s^{-1}	☑
kmc_unknown42_3_FADDCaspase_8_TNFalphaTNFR1ATRADDMADDcIAP2RIPTRAF2RAIDD	Michaelis constant of species FADD/Caspase_8 and enzyme TNF-alpha/TNFR1A/TRADD/MADD/cIAP2/RIP/TRAF2/RAIDD in reaction unknown42_3	0000027	1.0	mol	☑
hco_unknown42_3_TNFalphaTNFR1ATRADDMADDcIAP2RIPTRAF2RAIDD	Reaction cooperativity	0000382	1.0	dimensionless	☑

ID	Name	SBO	Value	Unit	Constant
kcrf-_unknown42_3-_TNFalphaTNFR1ATRADDMADDcIAP2RIPTRAF2RAIDD	Substrate catalytic rate constant of enzyme TNF-alpha/TNFR1A/TRADD/MADD/cIAP2/RIP/TRAF2/RAIDD in reaction unknown42_3	0000320	1.0	s^{-1}	☑
vmar-_unknown43	Revere maximal velocity of reaction unknown43	0000325	1.0	$mol \cdot s^{-1}$	☑
kmc-_unknown43-_RB1	Michaelis constant of species RB1 in reaction unknown43	0000027	1.0	mol	☑
hco-_unknown43	Reaction cooperativity	0000382	1.0	dimensionless	☑
vmaf-_unknown43	Forward maximal velocity of reaction unknown43	0000324	1.0	$mol \cdot s^{-1}$	☑
kmc-_unknown44_1-_TNFR1ABAG4TNFalpha	Michaelis constant of species TNFR1A/BAG4/TNF-alpha in reaction unknown44_1	0000027	1.0	mol	☑
vmar-_unknown44_1	Revere maximal velocity of reaction unknown44_1	0000325	1.0	$mol \cdot s^{-1}$	☑
kmc-_unknown44_1-_TNFalpha_1	Michaelis constant of species TNF-alpha in reaction unknown44_1	0000027	1.0	mol	☑
kmc-_unknown44_1-_TNFR1ABAG4	Michaelis constant of species TNFR1A/BAG4 in reaction unknown44_1	0000027	1.0	mol	☑
hco-_unknown44_1	Reaction cooperativity	0000382	1.0	dimensionless	☑

ID	Name	SBO	Value	Unit	Constant
vmaf-_unknown44_1	Forward maximal velocity of reaction unknown44_1	0000324	1.0	$mol \cdot s^{-1}$	☑
kmc-_unknown45_1-_BAG4	Michaelis constant of species BAG4 in reaction unknown45_1	0000027	1.0	mol	☑
kmc-_unknown45_1-_TNFalphaTNFR1A[illegible]	Michaelis constant of species TNF-alpha/TNFR1A/TRADD/MADD/cIAP2/RIP/TRAF2/RAIDD in reaction unknown45_1	0000027	1.0	mol	☑
vmar-_unknown45_1	Revere maximal velocity of reaction unknown45_1	0000325	1.0	$mol \cdot s^{-1}$	☑
kmc-_unknown45_1-_TNFR1ABAG4TNFalpha	Michaelis constant of species TNFR1A/BAG4/TNF-alpha in reaction unknown45_1	0000027	1.0	mol	☑
kmc-_unknown45_1-_cIAP2	Michaelis constant of species cIAP2 in reaction unknown45_1	0000027	1.0	mol	☑
kmc-_unknown45_1-_MADD	Michaelis constant of species MADD in reaction unknown45_1	0000027	1.0	mol	☑
kmc-_unknown45_1-_TRAF2	Michaelis constant of species TRAF2 in reaction unknown45_1	0000027	1.0	mol	☑
kmc-_unknown45_1-_TRADD	Michaelis constant of species TRADD in reaction unknown45_1	0000027	1.0	mol	☑

ID	Name	SBO	Value	Unit	Constant
kmc-_unknown45_1-_RAIDD	Michaelis constant of species RAIDD in reaction unknown45_1	0000027	1.0	mol	☑
kmc-_unknown45_1-_RIP	Michaelis constant of species RIP in reaction unknown45_1	0000027	1.0	mol	☑
hco-_unknown45_1	Reaction cooperativity	0000382	1.0	dimensionless	☑
vmaf-_unknown45_1	Forward maximal velocity of reaction unknown45_1	0000324	1.0	$mol \cdot s^{-1}$	☑
kmc-_unknown46-_FADD	Michaelis constant of species FADD in reaction unknown46	0000027	1.0	mol	☑
kmc-_unknown46-_Caspase_8	Michaelis constant of species Caspase-_8 in reaction unknown46	0000027	1.0	mol	☑
vmar-_unknown46	Revere maximal velocity of reaction unknown46	0000325	1.0	$mol \cdot s^{-1}$	☑
kmc-_unknown46-_FADDCaspase-_8	Michaelis constant of species FADD/Caspase-_8 in reaction unknown46	0000027	1.0	mol	☑
hco-_unknown46	Reaction cooperativity	0000382	1.0	dimensionless	☑
vmaf-_unknown46	Forward maximal velocity of reaction unknown46	0000324	1.0	$mol \cdot s^{-1}$	☑

ID	Name	SBO	Value	Unit	Constant
kmc-_unknown47-_I_kappa-_B_alpha-_degradation-_00	Michaelis constant of species I_kappa_B_alpha-_(degradation)_0-0 in reaction unknown47	0000027	1.0	mol	☑
kmc-_unknown47-_RelANF-_kappa_B1	Michaelis constant of species RelA/NF_kappa-_B1 in reaction unknown47	0000027	1.0	mol	☑
vmar-_unknown47	Revere maximal velocity of reaction unknown47	0000325	1.0	$mol \cdot s^{-1}$	☑
kmc-_unknown47-_NF_kappa-_B1RelAI-_kappa_B-_alpha_1	Michaelis constant of species NF-_kappa_B1/RelA/I-_kappa_B_alpha in reaction unknown47	0000027	1.0	mol	☑
hco-_unknown47	Reaction cooperativity	0000382	1.0	dimensionless	☑
vmaf-_unknown47	Forward maximal velocity of reaction unknown47	0000324	1.0	$mol \cdot s^{-1}$	☑
vmar-_unknown48	Revere maximal velocity of reaction unknown48	0000325	1.0	$mol \cdot s^{-1}$	☑
kmc-_unknown48-_RB1	Michaelis constant of species RB1 in reaction unknown48	0000027	1.0	mol	☑
hco-_unknown48	Reaction cooperativity	0000382	1.0	dimensionless	☑
vmaf-_unknown48	Forward maximal velocity of reaction unknown48	0000324	1.0	$mol \cdot s^{-1}$	☑

ID	Name	SBO	Value	Unit	Constant
vmar-_unknown49	Revere maximal velocity of reaction unknown49	0000325	1.0	$mol \cdot s^{-1}$	☑
kmc-_unknown49-_PP2A-_Heterotrimer	Michaelis constant of species PP2A-_Heterotrimer in reaction unknown49	0000027	1.0	mol	☑
hco-_unknown49	Reaction cooperativity	0000382	1.0	dimensionless	☑
vmaf-_unknown49	Forward maximal velocity of reaction unknown49	0000324	1.0	$mol \cdot s^{-1}$	☑
vmar-_unknown50	Revere maximal velocity of reaction unknown50	0000325	1.0	$mol \cdot s^{-1}$	☑
kmc-_unknown50-_PP2A-_Heterotrimer	Michaelis constant of species PP2A-_Heterotrimer in reaction unknown50	0000027	1.0	mol	☑
hco-_unknown50	Reaction cooperativity	0000382	1.0	dimensionless	☑
vmaf-_unknown50	Forward maximal velocity of reaction unknown50	0000324	1.0	$mol \cdot s^{-1}$	☑
kmc-_unknown51-_MEK1	Michaelis constant of species MEK1 in reaction unknown51	0000027	1.0	mol	☑
kmc-_unknown51-_MEK2	Michaelis constant of species MEK2 in reaction unknown51	0000027	1.0	mol	☑
vmar-_unknown51	Revere maximal velocity of reaction unknown51	0000325	1.0	$mol \cdot s^{-1}$	☑
kmc-_unknown51-_MEK2_1	Michaelis constant of species MEK2 in reaction unknown51	0000027	1.0	mol	☑

ID	Name	SBO	Value	Unit	Constant
kmc-_unknown51-_MEK1_1	Michaelis constant of species MEK1 in reaction unknown51	0000027	1.0	mol	☑
hco-_unknown51	Reaction cooperativity	0000382	1.0	dimensionless	☑
vmaf-_unknown51	Forward maximal velocity of reaction unknown51	0000324	1.0	$\mathrm{mol} \cdot \mathrm{s}^{-1}$	☑
kmc-_unknown53-_Free_Fatty-_acid	Michaelis constant of species Free_Fatty_acid in reaction unknown53	0000027	1.0	mol	☑
kmc-_unknown53-_sphingosine	Michaelis constant of species sphingosine in reaction unknown53	0000027	1.0	mol	☑
vmar-_unknown53	Revere maximal velocity of reaction unknown53	0000325	1.0	$\mathrm{mol} \cdot \mathrm{s}^{-1}$	☑
kmc-_unknown53-_ceramide	Michaelis constant of species ceramide in reaction unknown53	0000027	1.0	mol	☑
hco-_unknown53	Reaction cooperativity	0000382	1.0	dimensionless	☑
vmaf-_unknown53	Forward maximal velocity of reaction unknown53	0000324	1.0	$\mathrm{mol} \cdot \mathrm{s}^{-1}$	☑

6 Reactions

This model contains fifty Reactions. All reactions are listed in the following table and are subsequently described in detail. If a reaction is affected by one or more modifiers, the identifiers of the modifier species are written above the reaction arrow.

Table 5: Overview of all Reactions.

№	ID	Name	Reaction equation	SBO
1	unknown1_2	unknown1	$\text{PKR} \xrightleftharpoons{\text{RAX}} \text{PKR}$	0000176
2	unknown2	unknown2	$\text{ERK1} + \text{PKC\_delta} \xrightleftharpoons{\text{IGF1, ceramide}} \text{ERK1PKC\_delta}$	0000176
3	unknown3	unknown3	$\text{KSR} \xrightleftharpoons{\text{ceramide}} \text{KSR}$	0000176
4	unknown4_1	unknown4	$\text{RAF1} \xrightleftharpoons{\text{KSR}} \text{RAF1}$	0000176
5	unknown5_1	unknown5	$\text{Cytochrome\_C} \xrightleftharpoons{\text{BAX, BAD, BCL2}} \text{Cytochrome\_C\_1}$	0000176
6	unknown6	unknown6	$\text{sphingomyelin} \xrightleftharpoons{\text{GW4869, C11AG, Sphingosine1phosphate, EGF, Neutral}}$ [illegible] PHOSPHOCHOLINE	0000176
7	unknown7	unknown7	$\text{EIF2A} \xrightleftharpoons{\text{PKR}} \text{EIF2A}$	0000176
8	unknown8_1	unknown8	$\text{PAR4} + \text{ceramide} + \text{PKC\_zeta} \rightleftharpoons \text{PKC\_zetaPAR4} + \text{PKC\_zetaceramide}$	0000176
9	unknown9	unknown9	$\text{Neutral\_sphingomyelinase\_II} \xrightleftharpoons{\text{TNFalphaTNFR1ATRADDMADDcIAP2RIPTR}}$ [illegible]	0000176
10	unknown10_2	unknown10	$\text{Caspase\_8} \xrightleftharpoons{\text{RelANF\_kappa\_B1}} \text{Caspase\_8}$	0000176
11	unknown11	unknown11	$\text{AIF} \xrightleftharpoons{\text{BAD, BAX, BCL2}} \text{AIF\_1}$	0000176
12	unknown12	unknown12	$\text{BAX} \xrightleftharpoons{\text{GD3, ceramide}} \text{BAX}$	0000176
13	unknown13_1	unknown13	$\text{sphingosine} \xrightleftharpoons{\text{SPHK2}} \text{Sphingosine1phosphate}$	0000176
14	unknown14_1	unknown14	$\text{BID} \xrightleftharpoons{\text{TNFalphaTNFR1ATRADDMADDcIAP2RIPTRAF2RAIDD, Cathepsin\_Dcera}}$ [illegible]	0000176

Produced by SBML2LaTeX version 1.0 for JSBML

№	ID	Name	Reaction equation	SBO
15	unknown15_2	unknown15	MAP4K4 $\xrightleftharpoons{\text{TNFalphaTNFR1ATRADDMADDcIAP2RIPTRAF2RAIDD}}$ MAP4K4	0000176
16	unknown16_1	unknown16	MEKK1 $\xrightleftharpoons{\text{ceramide}}$ MEKK1	0000176
17	unknown17	unknown17	IKKs $\xrightleftharpoons{\text{MAP4K4}}$ IKKs	0000176
18	unknown18_1	unknown18	NF_kappa_B1RelAI_kappa_B_alpha $\xrightleftharpoons{\text{PKC\_zetaPAR4, IKKs}}$ NF_kappa_B1Rel	0000176
19	unknown19	unknown19	JNK1 $\xrightleftharpoons{\text{PKC\_zetaceramide, MKK4}}$ JNK1_1	0000176
20	unknown20	unknown20	Acid_Sphingomyelinase $\xrightleftharpoons{\text{TNFalphaTNFR1AFAN, TNFalphaTNFR1ATRADDMA}}$	0000176
21	unknown21	unknown21	MKK4_1 $\xrightleftharpoons{\text{MEKK1}}$ MKK4	0000176
22	unknown22	unknown22	Cathepsin_D + ceramide $\rightleftharpoons$ Cathepsin_Dceramide	0000176
23	unknown23	unknown23	sphingomyelin $\xrightleftharpoons{\text{EGF, Acid\_Sphingomyelinase}}$ ceramide	0000176
24	unknown24	unknown24	PP2A_Heterotrimer $\rightleftharpoons$ PP2A_Heterotrimer	0000176
25	unknown25_1	unknown25	AKT1 $\xrightleftharpoons{\text{PKC\_zetaceramide}}$ AKT1	0000176
26	unknown26	unknown26	SPHK2 $\xrightleftharpoons{\text{PDGFA}}$ SPHK2	0000176
27	unknown28	unknown28	PP2A_Heterotrimer $\rightleftharpoons$ PP2A_Heterotrimer	0000176
28	unknown30_1	unknown30	RAX $\xrightleftharpoons{\text{ceramide}}$ RAX	0000176
29	unknown31	unknown31	AKT1 $\rightleftharpoons$ AKT1	0000176
30	unknown32	unknown32	TNFR1A_1 + TNFalpha_1 + FAN $\rightleftharpoons$ TNFalphaTNFR1AFAN	0000176
31	unknown33_1	unknown33	ERK2 + ERK1 $\xrightleftharpoons{\text{MEK2}}$ ERK1 + ERK2	0000176
32	unknown34	unknown34	ERK2 + ERK1 $\xrightleftharpoons{\text{MEK1}}$ ERK1 + ERK2	0000176
33	unknown35	unknown35	BCL2 $\xrightleftharpoons{\text{PP2A\_Heterotrimer}}$ BCL2	0000176
34	unknown36	unknown36	BAD $\rightleftharpoons$ BAD	0000176
35	unknown37	unknown37	BAD $\xrightleftharpoons{\text{ceramide}}$ BAD	0000176

№	ID	Name	Reaction equation	SBO
36	unknown38	unknown38	ceramide $\xrightleftharpoons{\text{Acid\_Ceramidase}}$ sphingosine + Free_Fatty_acid	0000176
37	unknown39	unknown39	ceramide $\xrightleftharpoons{\text{PDGFA}}$ sphingosine + Free_Fatty_acid	0000176
38	unknown40	unknown40	ERK2 + ERK1 $\rightleftharpoons$ ERK1 + ERK2	0000176
39	unknown41	unknown41	MEK1_1 + MEK2_1 $\rightleftharpoons$ MEK2 + MEK1	0000176
40	unknown42_3	unknown42	FADDCaspase_8 $\xrightleftharpoons{\text{TNFalphaTNFR1ATRADDMADDcIAP2RIPTRAF2RAIDD}}$ Caspa FADD	0000176
41	unknown43	unknown43	RB1 $\xrightleftharpoons{\text{ceramide}}$ RB1	0000176
42	unknown44_1	unknown44	TNFR1ABAG4 + TNFalpha_1 $\rightleftharpoons$ TNFR1ABAG4TNFalpha	0000176
43	unknown45_1	unknown45	RIP + RAIDD + TRADD + TRAF2 + MADD + cIAP2 + TNFR1ABAG4TNFalpha $\rightleftharpoons$ TNFalphaTNFR1ATRADDMADDcIAP2RIPTRAF2RA BAG4	0000176
44	unknown46	unknown46	FADDCaspase_8 $\rightleftharpoons$ Caspase_8 + FADD	0000176
45	unknown47	unknown47	NF_kappa_B1RelAI_kappa_B_alpha_1 $\rightleftharpoons$ RelANF_kappa_B1 + I_kappa_B_alpha_degradation_00	0000176
46	unknown48	unknown48	RB1 $\rightleftharpoons$ RB1	0000176
47	unknown49	unknown49	PP2A_Heterotrimer $\xrightleftharpoons{\text{OKADAIC\_ACID}}$ PP2A_Heterotrimer	0000176
48	unknown50	unknown50	PP2A_Heterotrimer $\xrightleftharpoons{\text{ceramide}}$ PP2A_Heterotrimer	0000176
49	unknown51	unknown51	MEK1_1 + MEK2_1 $\xrightleftharpoons{\text{RAF1}}$ MEK2 + MEK1	0000176
50	unknown53	unknown53	ceramide $\rightleftharpoons$ sphingosine + Free_Fatty_acid	0000176

Produced by SBML2LaTeX version 1.0 for JSBML

6.1 Reaction unknown1_2

This is a reversible reaction of one reactant forming one product influenced by one modifier.

Name unknown1

SBO:0000176 biochemical reaction

Notes

MIRIAM Annotation This biological entity is described by urn:miriam:obo.eco:ECO%3A0000313.

Reaction equation

$$\mathtt{PKR} \xrightleftharpoons{\mathtt{RAX}} \mathtt{PKR} \quad (1)$$

Reactants

Table 6: Properties of each reactant.

Id	Name	SBO
PKR	unknown1	0000010

Modifiers

Table 7: Properties of each modifier.

Id	Name	SBO
RAX	mod_unknown1	0000013

Products

Table 8: Properties of each product.

Id	Name	SBO
PKR	unknown1	0000010

Kinetic law

SBO:0000528 common modular rate law

Derived unit $\mathrm{mol} \cdot \mathrm{s}^{-1}$

$$v_1 = \frac{\texttt{vmaf\_unknown1\_2} \cdot \left(\frac{[\text{PKR}] \cdot \text{vol}(\texttt{default})}{\texttt{kmc\_unknown1\_2\_PKR}}\right)^{\texttt{unknown1} \cdot \texttt{hco\_unknown1\_2}} - \texttt{vmar\_unknown1\_2} \cdot \left(\frac{[\text{PKR}] \cdot \text{vol}(\texttt{default})}{\texttt{kmc\_unknown1\_2\_PKR}}\right)^{\texttt{unknown1\_1} \cdot \texttt{h} \text{[illegible]}}}{\left(1 + \frac{[\text{PKR}] \cdot \text{vol}(\texttt{default})}{\texttt{kmc\_unknown1\_2\_PKR}}\right)^{\texttt{unknown1} \cdot \texttt{hco\_unknown1\_2}} + \left(1 + \frac{[\text{PKR}] \cdot \text{vol}(\texttt{default})}{\texttt{kmc\_unknown1\_2\_PKR}}\right)^{\texttt{unknown1\_1} \cdot \texttt{hco\_unknown1\_2}} - 1 \text{[illegible]}} \quad (2)$$

6.2 Reaction unknown2

This is a reversible reaction of two reactants forming one product influenced by two modifiers.

Name unknown2

SBO:0000176 biochemical reaction

Notes

MIRIAM Annotation This biological entity is described by urn:miriam:obo.eco:ECO%3A0000313.

Reaction equation

$$\texttt{ERK1} + \texttt{PKC\_delta} \xrightleftharpoons{\texttt{IGF1},\ \texttt{ceramide}} \texttt{ERK1PKC\_delta} \quad (3)$$

Reactants

Table 9: Properties of each reactant.

Id	Name	SBO
ERK1	unknown4	0000010
PKC_delta	unknown5	0000010

Modifiers

Table 10: Properties of each modifier.

Id	Name	SBO
IGF1	mod_unknown2_1	0000013
ceramide	mod_unknown2	0000013

Products

Table 11: Properties of each product.

Id	Name	SBO
ERK1PKC_delta	unknown8	0000010

Kinetic law

SBO:0000528 common modular rate law

Derived unit $\mathrm{mol} \cdot \mathrm{s}^{-1}$

$$v_2 = \frac{\mathtt{vmaf\_unknown2} \cdot \left(\frac{[\mathtt{ERK1}] \cdot \mathrm{vol}(\mathtt{cytoplasm})}{\mathtt{kmc\_unknown2\_ERK1}}\right)^{\mathtt{unknown4} \cdot \mathtt{hco\_unknown2}} \cdot \left(\frac{[\mathtt{PKC\_delta}] \cdot \mathrm{vol}(\mathtt{default})}{\mathtt{kmc\_unknown2\_PKC\_delta}}\right)^{\mathtt{unknown5} \cdot \mathtt{hco\_unknown2}} - \mathtt{vmar\_un}}{\left(1 + \frac{[\mathtt{ERK1}] \cdot \mathrm{vol}(\mathtt{cytoplasm})}{\mathtt{kmc\_unknown2\_ERK1}}\right)^{\mathtt{unknown4} \cdot \mathtt{hco\_unknown2}} \cdot \left(1 + \frac{[\mathtt{PKC\_delta}] \cdot \mathrm{vol}(\mathtt{default})}{\mathtt{kmc\_unknown2\_PKC\_delta}}\right)^{\mathtt{unknown5} \cdot \mathtt{hco\_unknown2}} + \left(1 + \frac{[\mathtt{ER}}{\mathtt{kmc}}\right.} \quad (4)$$

6.3 Reaction unknown3

This is a reversible reaction of one reactant forming one product influenced by one modifier.

Name unknown3

SBO:0000176 biochemical reaction

Notes

MIRIAM Annotation This biological entity is described by urn:miriam:obo.eco:ECO%3A0000313.

Reaction equation

$$\mathtt{KSR} \xrightleftharpoons{\mathtt{ceramide}} \mathtt{KSR} \quad (5)$$

Reactants

Table 12: Properties of each reactant.

Id	Name	SBO
KSR	unknown10	0000010

Modifiers

Table 13: Properties of each modifier.

Id	Name	SBO
ceramide	mod_unknown3	0000013

Products

Table 14: Properties of each product.

Id	Name	SBO
KSR	unknown10	0000010

Kinetic law

SBO:0000528 common modular rate law

Derived unit $\mathrm{mol} \cdot \mathrm{s}^{-1}$

$$v_3 = \frac{\mathtt{vmaf\_unknown3} \cdot \left(\frac{[\mathtt{KSR}] \cdot \mathrm{vol}(\mathtt{default})}{\mathtt{kmc\_unknown3\_KSR}}\right)^{\mathtt{unknown10} \cdot \mathtt{hco\_unknown3}} - \mathtt{vmar\_unknown3} \cdot \left(\frac{[\mathtt{KSR}] \cdot \mathrm{vol}(\mathtt{default})}{\mathtt{kmc\_unknown3\_KSR}}\right)^{\mathtt{unknown10\_1} \cdot \mathtt{hco\_unkno}\text{[illegible]}}}{\left(1 + \frac{[\mathtt{KSR}] \cdot \mathrm{vol}(\mathtt{default})}{\mathtt{kmc\_unknown3\_KSR}}\right)^{\mathtt{unknown10} \cdot \mathtt{hco\_unknown3}} + \left(1 + \frac{[\mathtt{KSR}] \cdot \mathrm{vol}(\mathtt{default})}{\mathtt{kmc\_unknown3\_KSR}}\right)^{\mathtt{unknown10\_1} \cdot \mathtt{hco\_unknown3}} - 1} \tag{6}$$

6.4 Reaction unknown4_1

This is a reversible reaction of one reactant forming one product influenced by one modifier.

Name unknown4

SBO:0000176 biochemical reaction

Notes

MIRIAM Annotation This biological entity is described by urn:miriam:obo.eco:ECO%3A0000313.

Reaction equation

$$\mathtt{RAF1} \xrightleftharpoons{\mathtt{KSR}} \mathtt{RAF1} \tag{7}$$

Reactants

Table 15: Properties of each reactant.

Id	Name	SBO
RAF1	hsa:5894	0000010

Modifiers

Table 16: Properties of each modifier.

Id	Name	SBO
KSR	mod_unknown4	0000013

Products

Table 17: Properties of each product.

Id	Name	SBO
RAF1	hsa:5894	0000010

Kinetic law

SBO:0000528 common modular rate law

Derived unit $\mathrm{mol} \cdot \mathrm{s}^{-1}$

$$v_4 = \frac{\mathtt{vmaf\_unknown4\_1} \cdot \left(\frac{[\mathtt{RAF1}] \cdot \mathrm{vol}(\mathtt{default})}{\mathtt{kmc\_unknown4\_1\_RAF1}}\right)^{\mathtt{hsa5894} \cdot \mathtt{hco\_unknown4\_1}} - \mathtt{vmar\_unknown4\_1} \cdot \left(\frac{[\mathtt{RAF1}] \cdot \mathrm{vol}(\mathtt{default})}{\mathtt{kmc\_unknown4\_1\_RAF1}}\right)^{\mathtt{hsa5894\_1} \cdot \mathtt{h}\text{[illegible]}}}{\left(1 + \frac{[\mathtt{RAF1}] \cdot \mathrm{vol}(\mathtt{default})}{\mathtt{kmc\_unknown4\_1\_RAF1}}\right)^{\mathtt{hsa5894} \cdot \mathtt{hco\_unknown4\_1}} + \left(1 + \frac{[\mathtt{RAF1}] \cdot \mathrm{vol}(\mathtt{default})}{\mathtt{kmc\_unknown4\_1\_RAF1}}\right)^{\mathtt{hsa5894\_1} \cdot \mathtt{hco\_unknown4\_1}} - 1} \quad (8)$$

6.5 Reaction unknown5_1

This is a reversible reaction of one reactant forming one product influenced by three modifiers.

Name unknown5

SBO:0000176 biochemical reaction

Notes

MIRIAM Annotation This biological entity is described by urn:miriam:pubmed:9873064.

This biological entity is described by urn:miriam:obo.eco:ECO%3A0000313.

Reaction equation

$$\texttt{Cytochrome\_C} \xrightleftharpoons{\texttt{BAX, BAD, BCL2}} \texttt{Cytochrome\_C\_1} \tag{9}$$

Reactants

Table 18: Properties of each reactant.

Id	Name	SBO
Cytochrome_C	unknown13	0000010

Modifiers

Table 19: Properties of each modifier.

Id	Name	SBO
BAX	mod_unknown5_1	0000013
BAD	mod_unknown5_2	0000013
BCL2	mod_unknown5	0000013

Products

Table 20: Properties of each product.

Id	Name	SBO
Cytochrome_C_1	unknown14	0000010

Kinetic law

SBO:0000528 common modular rate law

Derived unit $\mathrm{mol} \cdot \mathrm{s}^{-1}$

$$v_5 = \frac{\texttt{vmaf\_unknown5\_1} \cdot \left(\frac{[\texttt{Cytochrome\_C}] \cdot \mathrm{vol}(\texttt{mitochondria})}{\texttt{kmc\_unknown5\_1\_Cytochrome\_C}}\right)^{\texttt{unknown13} \cdot \texttt{hco\_unknown5\_1}} - \texttt{vmar\_unknown5\_1} \cdot \left(\frac{[\texttt{Cytochrome\_C\_1}] \cdot \mathrm{vo}\text{[illegible]}}{\texttt{kmc\_unknown5\_1\_Cyt}\text{[illegible]}}\right.\text{[illegible]}}{\left(1 + \frac{[\texttt{Cytochrome\_C}] \cdot \mathrm{vol}(\texttt{mitochondria})}{\texttt{kmc\_unknown5\_1\_Cytochrome\_C}}\right)^{\texttt{unknown13} \cdot \texttt{hco\_unknown5\_1}} + \left(1 + \frac{[\texttt{Cytochrome\_C\_1}] \cdot \mathrm{vol}(\texttt{cytoplasm})}{\texttt{kmc\_unknown5\_1\_Cytochrome\_C\_1}}\right)^{\texttt{unknown}\text{[illegible]}}} \tag{10}$$

6.6 Reaction unknown6

This is a reversible reaction of one reactant forming two products influenced by five modifiers.

Name unknown6

SBO:0000176 biochemical reaction

Notes

MIRIAM Annotation This biological entity is described by urn:miriam:obo.eco:ECO%3A0000313.

Reaction equation

$$\texttt{sphingomyelin} \xrightleftharpoons{\texttt{GW4869, C11AG, Sphingosine1phosphate, EGF, Neutral\_sphingomyelinase\_II}} \texttt{ceramide} + \texttt{P}\text{[illegible]} \quad (11)$$

Reactants

Table 21: Properties of each reactant.

Id	Name	SBO
sphingomyelin	unknown16	0000010

Modifiers

Table 22: Properties of each modifier.

Id	Name	SBO
GW4869	mod_unknown6_3	0000013
C11AG	mod_unknown6_4	0000013
Sphingosine1phosphate	mod_unknown6_1	0000013
EGF	mod_unknown6_2	0000013
Neutral_sphingomyelinase_II	mod_unknown6	0000013

Products

Table 23: Properties of each product.

Id	Name	SBO
ceramide	unknown3	0000010
PHOSPHOCHOLINE	unknown18	0000010

Kinetic law

SBO:0000528 common modular rate law

Derived unit $\mathrm{mol} \cdot \mathrm{s}^{-1}$

$$v_6 = \frac{\texttt{vmaf\_unknown6} \cdot \left(\frac{[\texttt{sphingomyelin}] \cdot \mathrm{vol}(\texttt{default})}{\texttt{kmc\_unknown6\_sphingomyelin}}\right)^{\texttt{unknown16} \cdot \texttt{hco\_unknown6}} - \texttt{vmar\_unknown6} \cdot \left(\frac{[\texttt{ceramide}] \cdot \mathrm{vol}(\texttt{default})}{\texttt{kmc\_unknown6\_ceramide}}\right)^{\texttt{unkno}[illegible]}}{\left(1 + \frac{[\texttt{sphingomyelin}] \cdot \mathrm{vol}(\texttt{default})}{\texttt{kmc\_unknown6\_sphingomyelin}}\right)^{\texttt{unknown16} \cdot \texttt{hco\_unknown6}} + \left(1 + \frac{[\texttt{ceramide}] \cdot \mathrm{vol}(\texttt{default})}{\texttt{kmc\_unknown6\_ceramide}}\right)^{\texttt{unknown3\_1} \cdot \texttt{hco\_unknown6}} \cdot [illegible]} \quad (12)$$

6.7 Reaction unknown7

This is a reversible reaction of one reactant forming one product influenced by one modifier.

Name unknown7

SBO:0000176 biochemical reaction

Notes

MIRIAM Annotation This biological entity is described by urn:miriam:obo.eco:ECO%3A0000313.

Reaction equation

$$\texttt{EIF2A} \xrightleftharpoons{\texttt{PKR}} \texttt{EIF2A} \quad (13)$$

Reactants

Table 24: Properties of each reactant.

Id	Name	SBO
EIF2A	hsa:83939	0000010

Modifiers

Table 25: Properties of each modifier.

Id	Name	SBO
PKR	mod_unknown7	0000013

Products

Table 26: Properties of each product.

Id	Name	SBO
EIF2A	hsa:83939	0000010

Kinetic law

SBO:0000528 common modular rate law

Derived unit $\mathrm{mol} \cdot \mathrm{s}^{-1}$

$$v_7 = \frac{\mathtt{vmaf\_unknown7} \cdot \left(\frac{[\mathtt{EIF2A}] \cdot \mathrm{vol}(\mathtt{default})}{\mathtt{kmc\_unknown7\_EIF2A}}\right)^{\mathtt{hsa83939} \cdot \mathtt{hco\_unknown7}} - \mathtt{vmar\_unknown7} \cdot \left(\frac{[\mathtt{EIF2A}] \cdot \mathrm{vol}(\mathtt{default})}{\mathtt{kmc\_unknown7\_EIF2A}}\right)^{\mathtt{hsa83939\_1} \cdot \mathtt{hco\_unk}\text{[illegible]}}}{\left(1 + \frac{[\mathtt{EIF2A}] \cdot \mathrm{vol}(\mathtt{default})}{\mathtt{kmc\_unknown7\_EIF2A}}\right)^{\mathtt{hsa83939} \cdot \mathtt{hco\_unknown7}} + \left(1 + \frac{[\mathtt{EIF2A}] \cdot \mathrm{vol}(\mathtt{default})}{\mathtt{kmc\_unknown7\_EIF2A}}\right)^{\mathtt{hsa83939\_1} \cdot \mathtt{hco\_unknown7}} - 1} \quad (14)$$

6.8 Reaction unknown8_1

This is a reversible reaction of three reactants forming two products.

Name unknown8

SBO:0000176 biochemical reaction

Notes

MIRIAM Annotation This biological entity is described by urn:miriam:pubmed:15901738.

This biological entity is described by urn:miriam:obo.eco:ECO%3A0000313.

Reaction equation

$$\mathtt{PAR4} + \mathtt{ceramide} + \mathtt{PKC\_zeta} \rightleftharpoons \mathtt{PKC\_zetaPAR4} + \mathtt{PKC\_zetaceramide} \quad (15)$$

Reactants

Table 27: Properties of each reactant.

Id	Name	SBO
PAR4	unknown25	0000010
ceramide	unknown3	0000010
PKC_zeta	unknown27	0000010

Products

Table 28: Properties of each product.

Id	Name	SBO
PKC_zetaPAR4	unknown30	0000010
PKC_zetaceramide	unknown33	0000010

Kinetic law

SBO:0000528 common modular rate law

Derived unit $\mathrm{mol} \cdot \mathrm{s}^{-1}$

$$v_8 = \frac{\mathtt{vmaf\_unknown8\_1} \cdot \left(\frac{[\mathtt{PAR4}] \cdot \mathrm{vol}(\mathtt{default})}{\mathtt{kmc\_unknown8\_1\_PAR4}}\right)^{\mathtt{unknown25} \cdot \mathtt{hco\_unknown8\_1}} \cdot \left(\frac{[\mathtt{ceramide}] \cdot \mathrm{vol}(\mathtt{default})}{\mathtt{kmc\_unknown8\_1\_ceramide}}\right)^{\mathtt{unknown3\_2} \cdot \mathtt{hco\_unknown8\_1}} \cdot (}{\left(1 + \frac{[\mathtt{PAR4}] \cdot \mathrm{vol}(\mathtt{default})}{\mathtt{kmc\_unknown8\_1\_PAR4}}\right)^{\mathtt{unknown25} \cdot \mathtt{hco\_unknown8\_1}} \cdot \left(1 + \frac{[\mathtt{ceramide}] \cdot \mathrm{vol}(\mathtt{default})}{\mathtt{kmc\_unknown8\_1\_ceramide}}\right)^{\mathtt{unknown3\_2} \cdot \mathtt{hco\_unknown8\_1}} \cdot (1 +} \quad (16)$$

6.9 Reaction unknown9

This is a reversible reaction of one reactant forming one product influenced by three modifiers.

Name unknown9

SBO:0000176 biochemical reaction

Notes

MIRIAM Annotation This biological entity is described by urn:miriam:obo.go:GO%3A0006979.

This biological entity is described by urn:miriam:obo.eco:ECO%3A0000313.

Reaction equation

$$\texttt{Neutral\_sphingomyelinase\_II} \xrightleftharpoons{\texttt{TNFalphaTNFR1ATRADDMADDcIAP2RIPTRAF2RAIDD}, \texttt{TNFalphaTNFR1AFAN}, \texttt{glut}} \tag{17}$$

Reactants

Table 29: Properties of each reactant.

Id	Name	SBO
Neutral_sphingomyelinase_II	unknown15	0000010

Modifiers

Table 30: Properties of each modifier.

Id	Name	SBO
TNFalphaTNFR1ATRADDMADDcIAP2RIPTRAF2RAIDD	mod_unknown9_1	0000460
TNFalphaTNFR1AFAN	mod_unknown9_2	0000460
glutathione	mod_unknown9	0000013

Products

Table 31: Properties of each product.

Id	Name	SBO
Neutral_sphingomyelinase_II	unknown15	0000010

Kinetic law

SBO:0000528 common modular rate law

Derived unit $\mathrm{mol} \cdot \mathrm{s}^{-1}$

$$v_9 = [\texttt{TNFalphaTNFR1ATRADDMADDcIAP2RIPTRAF2RAIDD}] \cdot \mathrm{vol}\left(\texttt{default}\right) \cdot \frac{\texttt{kcrf\_unknown9\_TNFalphaTNFR1ATRADDMADDcIAP2RIPTRAF2RAIDD} \cdot \left(\frac{[\texttt{Neutral\_sphingomyelin}\text{[illegible]}}{\texttt{kmc\_unknown9\_Neutral\_sphingomyelinase\_II\_TNF}\text{[illegible]}}\right.}{\left(1 + \frac{[\texttt{Neutral\_sphingomyelinas}\text{[illegible]}}{\texttt{kmc\_unknown9\_Neutral\_sphingomyelinase\_II\_TNFal}\text{[illegible]}}\right.} \quad (18)$$

$$+ [\texttt{TNFalphaTNFR1AFAN}] \cdot \mathrm{vol}\left(\texttt{default}\right) \cdot \frac{\texttt{kcrf\_unknown9\_TNFalphaTNFR1AFAN} \cdot \left(\frac{[\texttt{Neutral\_sphingomyelinase\_II}] \cdot \mathrm{vol}(\texttt{default})}{\texttt{kmc\_unknown9\_Neutral\_sphingomyelinase\_II\_TNFalphaTNFR1AFAN}}\right)^{\texttt{unknown15} \cdot \texttt{hco\_unk}\text{[illegible]}}}{\left(1 + \frac{[\texttt{Neutral\_sphingomyelinase\_II}] \cdot \mathrm{vol}(\texttt{default})}{\texttt{kmc\_unknown9\_Neutral\_sphingomyelinase\_II\_TNFalphaTNFR1AFAN}}\right)^{\texttt{unknown15} \cdot \texttt{hco\_unkno}\text{[illegible]}}}$$

6.10 Reaction unknown10_2

This is a reversible reaction of one reactant forming one product influenced by one modifier.

Name unknown10

SBO:0000176 biochemical reaction

Notes

MIRIAM Annotation This biological entity is described by urn:miriam:obo.eco:ECO%3A0000313.

Reaction equation

$$\texttt{Caspase\_8} \xrightleftharpoons{\texttt{RelANF\_kappa\_B1}} \texttt{Caspase\_8} \quad (19)$$

Reactants

Table 32: Properties of each reactant.

Id	Name	SBO
Caspase_8	unknown42	0000010

Modifiers

Table 33: Properties of each modifier.

Id	Name	SBO
RelANF_kappa_B1	mod_unknown10	0000460

Products

Table 34: Properties of each product.

Id	Name	SBO
Caspase_8	unknown42	0000010

Kinetic law

SBO:0000528 common modular rate law

Derived unit $\mathrm{mol} \cdot \mathrm{s}^{-1}$

$$v_{10} = [\texttt{RelANF\_kappa\_B1}] \cdot \mathrm{vol}\left(\texttt{cytoplasm}\right) \cdot \frac{\texttt{kcrf\_unknown10\_2\_RelANF\_kappa\_B1} \cdot \left(\frac{[\texttt{Caspase\_8}] \cdot \mathrm{vol}(\texttt{cytoplasm})}{\texttt{kmc\_unknown10\_2\_Caspase\_8\_RelANF\_kappa\_B1}}\right)^{\texttt{unknown42} \cdot \texttt{hco\_unknown10\_2\_RelANF\_kap}\text{[illegible]}}}{\left(1 + \frac{[\texttt{Caspase\_8}] \cdot \mathrm{vol}(\texttt{cytoplasm})}{\texttt{kmc\_unknown10\_2\_Caspase\_8\_RelANF\_kappa\_B1}}\right)^{\texttt{unknown42} \cdot \texttt{hco\_unknown10\_2\_RelANF\_kappa}\text{[illegible]}}} \quad (20)$$

6.11 Reaction unknown11

This is a reversible reaction of one reactant forming one product influenced by three modifiers.

Name unknown11

SBO:0000176 biochemical reaction

Notes

MIRIAM Annotation This biological entity is described by urn:miriam:obo.eco:ECO%3A0000313.

Reaction equation

$$\texttt{AIF} \xrightleftharpoons{\texttt{BAD, BAX, BCL2}} \texttt{AIF\_1} \quad (21)$$

Reactants

Table 35: Properties of each reactant.

Id	Name	SBO
AIF	unknown44	0000010

Modifiers

Table 36: Properties of each modifier.

Id	Name	SBO
BAD	mod_unknown11_2	0000013
BAX	mod_unknown11_1	0000013
BCL2	mod_unknown11	0000013

Products

Table 37: Properties of each product.

Id	Name	SBO
AIF_1	unknown45	0000010

Kinetic law

SBO:0000528 common modular rate law

Derived unit $\mathrm{mol} \cdot \mathrm{s}^{-1}$

$$v_{11} = \frac{\mathtt{vmaf\_unknown11} \cdot \left(\frac{[\mathtt{AIF}] \cdot \mathrm{vol}(\mathtt{mitochondria})}{\mathtt{kmc\_unknown11\_AIF}}\right)^{\mathtt{unknown44} \cdot \mathtt{hco\_unknown11}} - \mathtt{vmar\_unknown11} \cdot \left(\frac{[\mathtt{AIF\_1}] \cdot \mathrm{vol}(\mathtt{cytoplasm})}{\mathtt{kmc\_unknown11\_AIF\_1}}\right)^{\mathtt{unknown}[illegible]}}{\left(1 + \frac{[\mathtt{AIF}] \cdot \mathrm{vol}(\mathtt{mitochondria})}{\mathtt{kmc\_unknown11\_AIF}}\right)^{\mathtt{unknown44} \cdot \mathtt{hco\_unknown11}} + \left(1 + \frac{[\mathtt{AIF\_1}] \cdot \mathrm{vol}(\mathtt{cytoplasm})}{\mathtt{kmc\_unknown11\_AIF\_1}}\right)^{\mathtt{unknown45} \cdot \mathtt{hco\_unknown11}} - [illegible]} \quad (22)$$

6.12 Reaction unknown12

This is a reversible reaction of one reactant forming one product influenced by two modifiers.

Name unknown12

SBO:0000176 biochemical reaction

Notes

MIRIAM Annotation This biological entity is described by urn:miriam:obo.eco:ECO%3A0000313.

Reaction equation

$$\mathtt{BAX} \xrightleftharpoons{\mathtt{GD3},\ \mathtt{ceramide}} \mathtt{BAX} \quad (23)$$

Reactants

Table 38: Properties of each reactant.

Id	Name	SBO
BAX	hsa:581	0000010

Modifiers

Table 39: Properties of each modifier.

Id	Name	SBO
GD3	mod_unknown12_1	0000013
ceramide	mod_unknown12	0000013

Products

Table 40: Properties of each product.

Id	Name	SBO
BAX	hsa:581	0000010

Kinetic law

SBO:0000528 common modular rate law

Derived unit $\mathrm{mol} \cdot \mathrm{s}^{-1}$

$$v_{12} = \frac{\mathtt{vmaf\_unknown12} \cdot \left(\frac{[\mathtt{BAX}]\cdot \mathrm{vol}(\mathtt{default})}{\mathtt{kmc\_unknown12\_BAX}}\right)^{\mathtt{hsa581} \cdot \mathtt{hco\_unknown12}} - \mathtt{vmar\_unknown12} \cdot \left(\frac{[\mathtt{BAX}]\cdot \mathrm{vol}(\mathtt{default})}{\mathtt{kmc\_unknown12\_BAX}}\right)^{\mathtt{hsa581\_1} \cdot \mathtt{hco\_unkno}}}{\left(1 + \frac{[\mathtt{BAX}]\cdot \mathrm{vol}(\mathtt{default})}{\mathtt{kmc\_unknown12\_BAX}}\right)^{\mathtt{hsa581} \cdot \mathtt{hco\_unknown12}} + \left(1 + \frac{[\mathtt{BAX}]\cdot \mathrm{vol}(\mathtt{default})}{\mathtt{kmc\_unknown12\_BAX}}\right)^{\mathtt{hsa581\_1} \cdot \mathtt{hco\_unknown12}} - 1} \quad (24)$$

6.13 Reaction unknown13_1

This is a reversible reaction of one reactant forming one product influenced by one modifier.

Name unknown13

SBO:0000176 biochemical reaction

Notes

MIRIAM Annotation This biological entity is described by urn:miriam:obo.eco:ECO%3A0000313.

Reaction equation

$$\texttt{sphingosine} \xrightleftharpoons{\texttt{SPHK2}} \texttt{Sphingosine1phosphate} \tag{25}$$

Reactants

Table 41: Properties of each reactant.

Id	Name	SBO
sphingosine	unknown54	0000010

Modifiers

Table 42: Properties of each modifier.

Id	Name	SBO
SPHK2	mod_unknown13	0000013

Products

Table 43: Properties of each product.

Id	Name	SBO
Sphingosine1phosphate	unknown55	0000010

Kinetic law

SBO:0000528 common modular rate law

Derived unit $\mathrm{mol} \cdot \mathrm{s}^{-1}$

$$v_{13} = \frac{\texttt{vmaf\_unknown13\_1} \cdot \left(\frac{[\texttt{sphingosine}] \cdot \mathrm{vol}(\texttt{default})}{\texttt{kmc\_unknown13\_1\_sphingosine}}\right)^{\texttt{unknown54} \cdot \texttt{hco\_unknown13\_1}} - \texttt{vmar\_unknown13\_1} \cdot \left(\frac{[\texttt{Sphingosine1pho}\text{[illegible]}}{\texttt{kmc\_unknown13\_1\_S}\text{[illegible]}}\right.}{\left(1 + \frac{[\texttt{sphingosine}] \cdot \mathrm{vol}(\texttt{default})}{\texttt{kmc\_unknown13\_1\_sphingosine}}\right)^{\texttt{unknown54} \cdot \texttt{hco\_unknown13\_1}} + \left(1 + \frac{[\texttt{Sphingosine1phosphate}] \cdot \mathrm{vol}(\texttt{default})}{\texttt{kmc\_unknown13\_1\_Sphingosine1phosphate}}\right.\text{[illegible]}} \tag{26}$$

6.14 Reaction unknown14_1

This is a reversible reaction of one reactant forming one product influenced by two modifiers.

Name unknown14

SBO:0000176 biochemical reaction

Notes

MIRIAM Annotation This biological entity is described by urn:miriam:obo.eco:ECO%3A0000313.

Reaction equation

$$\text{BID} \xrightleftharpoons{\text{TNFalphaTNFR1ATRADDMADDcIAP2RIPTRAF2RAIDD, Cathepsin\_Dceramide}} \text{BID} \quad (27)$$

Reactants

Table 44: Properties of each reactant.

Id	Name	SBO
BID	hsa:637	0000010

Modifiers

Table 45: Properties of each modifier.

Id	Name	SBO
TNFalphaTNFR1ATRADDMADDcIAP2RIPTRAF2RAIDD	mod_unknown14_1	0000460
Cathepsin_Dceramide	mod_unknown14	0000460

Products

Table 46: Properties of each product.

Id	Name	SBO
BID	hsa:637	0000010

Kinetic law

SBO:0000528 common modular rate law

Derived unit $\mathrm{mol} \cdot \mathrm{s}^{-1}$

$$v_{14} = [\mathtt{TNFalphaTNFR1ATRADDMADDcIAP2RIPTRAF2RAIDD}] \cdot \mathrm{vol}\left(\mathtt{default}\right) \cdot \frac{\mathtt{kcrf\_unknown14\_1\_TNFalphaTNFR1ATRADDMADDcIAP2RIPTRAF2RAIDD} \cdot \left(\frac{[\mathtt{BID}] \cdot \mathrm{vol}(\mathtt{cytoplasm})}{\mathtt{kmc\_unknown14\_1\_BID\_TNFalphaTNFR1ATRADD}\text{[illegible]}}\right.\text{[illegible]}}{\left(1 + \frac{[\mathtt{BID}] \cdot \mathrm{vol}(\mathtt{cytoplasm})}{\mathtt{kmc\_unknown14\_1\_BID\_TNFalphaTNFR1ATRADDMA}\text{[illegible]}}\right.\text{[illegible]}} + [\mathtt{Cathepsin\_Dceramide}] \cdot \mathrm{vol}\left(\mathtt{default}\right) \cdot \frac{\mathtt{kcrf\_unknown14\_1\_Cathepsin\_Dceramide} \cdot \left(\frac{[\mathtt{BID}] \cdot \mathrm{vol}(\mathtt{cytoplasm})}{\mathtt{kmc\_unknown14\_1\_BID\_Cathepsin\_Dceramide}}\right)^{\mathtt{hsa637} \cdot \mathtt{hco\_unknown14\_1\_Cathepsi}\text{[illegible]}}}{\left(1 + \frac{[\mathtt{BID}] \cdot \mathrm{vol}(\mathtt{cytoplasm})}{\mathtt{kmc\_unknown14\_1\_BID\_Cathepsin\_Dceramide}}\right)^{\mathtt{hsa637} \cdot \mathtt{hco\_unknown14\_1\_Cathepsin\_}\text{[illegible]}}} \quad (28)$$

6.15 Reaction unknown15_2

This is a reversible reaction of one reactant forming one product influenced by one modifier.

Name unknown15

SBO:0000176 biochemical reaction

Notes

MIRIAM Annotation This biological entity is described by urn:miriam:obo.eco:ECO%3A0000313.

Reaction equation

$$\mathtt{MAP4K4} \xrightleftharpoons{\mathtt{TNFalphaTNFR1ATRADDMADDcIAP2RIPTRAF2RAIDD}} \mathtt{MAP4K4} \quad (29)$$

Reactants

Table 47: Properties of each reactant.

Id	Name	SBO
MAP4K4	hsa:9448	0000010

Modifiers

Table 48: Properties of each modifier.

Id	Name	SBO
TNFalphaTNFR1ATRADDMADDcIAP2RIPTRAF2RAIDD	mod_unknown15	0000460

Products

Table 49: Properties of each product.

Id	Name	SBO
MAP4K4	hsa:9448	0000010

Kinetic law

SBO:0000528 common modular rate law

Derived unit $\mathrm{mol} \cdot \mathrm{s}^{-1}$

$$v_{15} = [\mathtt{TNFalphaTNFR1ATRADDMADDcIAP2RIPTRAF2RAIDD}] \cdot \mathrm{vol}\left(\mathtt{default}\right) \tag{30}$$
$$\cdot \frac{\mathtt{kcrf\_unknown15\_2\_TNFalphaTNFR1ATRADDMADDcIAP2RIPTRAF2RAIDD} \cdot \left(\frac{[\mathtt{MAP4K4}] \cdot \mathrm{vol}(\mathtt{defau}\text{[illegible]}}{\mathtt{kmc\_unknown15\_2\_MAP4K4\_TNFalphaTNFR1ATRA}\text{[illegible]}}\right.}{\left(1 + \frac{[\mathtt{MAP4K4}] \cdot \mathrm{vol}(\mathtt{default})}{\mathtt{kmc\_unknown15\_2\_MAP4K4\_TNFalphaTNFR1ATRADD}\text{[illegible]}}\right.}$$

6.16 Reaction unknown16_1

This is a reversible reaction of one reactant forming one product influenced by one modifier.

Name unknown16

SBO:0000176 biochemical reaction

Notes

MIRIAM Annotation This biological entity is described by urn:miriam:obo.eco:ECO%3A0000313.

Reaction equation

$$\mathtt{MEKK1} \xrightleftharpoons{\mathtt{ceramide}} \mathtt{MEKK1} \tag{31}$$

Reactants

Table 50: Properties of each reactant.

Id	Name	SBO
MEKK1	unknown61	0000010

Modifiers

Table 51: Properties of each modifier.

Id	Name	SBO
ceramide	mod_unknown16	0000013

Products

Table 52: Properties of each product.

Id	Name	SBO
MEKK1	unknown61	0000010

Kinetic law

SBO:0000528 common modular rate law

Derived unit $\mathrm{mol} \cdot \mathrm{s}^{-1}$

$$v_{16} = \frac{\mathtt{vmaf\_unknown16\_1} \cdot \left(\frac{[\mathtt{MEKK1}] \cdot \mathrm{vol}(\mathtt{default})}{\mathtt{kmc\_unknown16\_1\_MEKK1}}\right)^{\mathtt{unknown61} \cdot \mathtt{hco\_unknown16\_1}} - \mathtt{vmar\_unknown16\_1} \cdot \left(\frac{[\mathtt{MEKK1}] \cdot \mathrm{vol}(\mathtt{default})}{\mathtt{kmc\_unknown16\_1\_MEKK1}}\right)^{[illegible]}}{\left(1 + \frac{[\mathtt{MEKK1}] \cdot \mathrm{vol}(\mathtt{default})}{\mathtt{kmc\_unknown16\_1\_MEKK1}}\right)^{\mathtt{unknown61} \cdot \mathtt{hco\_unknown16\_1}} + \left(1 + \frac{[\mathtt{MEKK1}] \cdot \mathrm{vol}(\mathtt{default})}{\mathtt{kmc\_unknown16\_1\_MEKK1}}\right)^{\mathtt{unknown61\_1} \cdot \mathtt{hco\_unknow}[illegible]}} \tag{32}$$

6.17 Reaction unknown17

This is a reversible reaction of one reactant forming one product influenced by one modifier.

Name unknown17

SBO:0000176 biochemical reaction

Notes

MIRIAM Annotation This biological entity is described by urn:miriam:obo.eco:ECO%3A0000313.

Reaction equation

$$\texttt{IKKs} \xrightleftharpoons{\texttt{MAP4K4}} \texttt{IKKs} \tag{33}$$

Reactants

Table 53: Properties of each reactant.

Id	Name	SBO
IKKs	unknown63	0000010

Modifiers

Table 54: Properties of each modifier.

Id	Name	SBO
MAP4K4	mod_unknown17	0000013

Products

Table 55: Properties of each product.

Id	Name	SBO
IKKs	unknown63	0000010

Kinetic law

SBO:0000528 common modular rate law

Derived unit $\mathrm{mol} \cdot \mathrm{s}^{-1}$

$$v_{17} = \frac{\texttt{vmaf\_unknown17} \cdot \left(\frac{[\texttt{IKKs}] \cdot \mathrm{vol}(\texttt{default})}{\texttt{kmc\_unknown17\_IKKs}}\right)^{\texttt{unknown63} \cdot \texttt{hco\_unknown17}} - \texttt{vmar\_unknown17} \cdot \left(\frac{[\texttt{IKKs}] \cdot \mathrm{vol}(\texttt{default})}{\texttt{kmc\_unknown17\_IKKs}}\right)^{\texttt{unknown63\_1} \cdot \texttt{h}[illegible]}}{\left(1 + \frac{[\texttt{IKKs}] \cdot \mathrm{vol}(\texttt{default})}{\texttt{kmc\_unknown17\_IKKs}}\right)^{\texttt{unknown63} \cdot \texttt{hco\_unknown17}} + \left(1 + \frac{[\texttt{IKKs}] \cdot \mathrm{vol}(\texttt{default})}{\texttt{kmc\_unknown17\_IKKs}}\right)^{\texttt{unknown63\_1} \cdot \texttt{hco\_unknown17}} - 1} \tag{34}$$

6.18 Reaction unknown18_1

This is a reversible reaction of one reactant forming one product influenced by two modifiers.

Name unknown18

SBO:0000176 biochemical reaction

Notes

MIRIAM Annotation This biological entity is described by urn:miriam:obo.eco:ECO%3A0000313.

Reaction equation

$$\texttt{NF\_kappa\_B1RelAI\_kappa\_B\_alpha} \xrightleftharpoons{\texttt{PKC\_zetaPAR4, IKKs}} \texttt{NF\_kappa\_B1RelAI\_kappa\_B\_alpha\_1} \quad (35)$$

Reactants

Table 56: Properties of each reactant.

Id	Name	SBO
NF_kappa_B1RelAI_kappa_B_alpha	unknown67	0000010

Modifiers

Table 57: Properties of each modifier.

Id	Name	SBO
PKC_zetaPAR4	mod_unknown18_1	0000460
IKKs	mod_unknown18	0000013

Products

Table 58: Properties of each product.

Id	Name	SBO
NF_kappa_B1RelAI_kappa_B_alpha_1	unknown70	0000010

Kinetic law

SBO:0000528 common modular rate law

Derived unit $\mathrm{mol} \cdot \mathrm{s}^{-1}$

$$v_{18} = [\mathtt{PKC\_zetaPAR4}] \cdot \mathrm{vol}\left(\mathtt{default}\right) \cdot \frac{\mathtt{kcrf\_unknown18\_1\_PKC\_zetaPAR4} \cdot \left(\frac{[\mathtt{NF\_kappa\_B1RelAI\_kappa\_B\_alpha}] \cdot \mathrm{vol}(\mathtt{cytoplasm})}{\mathtt{kmc\_unknown18\_1\_NF\_kappa\_B1RelAI\_kappa\_B\_alpha\_PKC\_zetaPAR4}}\right)^{\mathtt{unknown67} \cdot \mathtt{hco\_unknown}}}{\left(1 + \frac{[\mathtt{NF\_kappa\_B1RelAI\_kappa\_B\_alpha}] \cdot \mathrm{vol}(\mathtt{cytoplasm})}{\mathtt{kmc\_unknown18\_1\_NF\_kappa\_B1RelAI\_kappa\_B\_alpha\_PKC\_zetaPAR4}}\right)^{\mathtt{unknown67} \cdot \mathtt{hco\_unknown18}}} \quad (36)$$

6.19 Reaction unknown19

This is a reversible reaction of one reactant forming one product influenced by two modifiers.

Name unknown19

SBO:0000176 biochemical reaction

Notes

MIRIAM Annotation This biological entity is described by urn:miriam:obo.eco:ECO%3A0000313.

Reaction equation

$$\mathtt{JNK1} \xrightleftharpoons{\mathtt{PKC\_zetaceramide},\ \mathtt{MKK4}} \mathtt{JNK1\_1} \quad (37)$$

Reactants

Table 59: Properties of each reactant.

Id	Name	SBO
JNK1	unknown72	0000010

Modifiers

Table 60: Properties of each modifier.

Id	Name	SBO
PKC_zetaceramide	mod_unknown19_1	0000460
MKK4	mod_unknown19	0000013

Products

Table 61: Properties of each product.

Id	Name	SBO
JNK1_1	unknown73	0000010

Kinetic law

SBO:0000528 common modular rate law

Derived unit $\mathrm{mol} \cdot \mathrm{s}^{-1}$

$$v_{19} = [\mathtt{PKC\_zetaceramide}] \cdot \mathrm{vol}(\mathtt{default}) \cdot \frac{\mathtt{kcrf\_unknown19\_PKC\_zetaceramide} \cdot \left(\frac{[\mathtt{JNK1}] \cdot \mathrm{vol}(\mathtt{cytoplasm})}{\mathtt{kmc\_unknown19\_JNK1\_PKC\_zetaceramide}}\right)^{\mathtt{unknown72} \cdot \mathtt{hco\_unknown19\_PKC\_zetaceramide}}}{\left(1 + \frac{[\mathtt{JNK1}] \cdot \mathrm{vol}(\mathtt{cytoplasm})}{\mathtt{kmc\_unknown19\_JNK1\_PKC\_zetaceramide}}\right)^{\mathtt{unknown72} \cdot \mathtt{hco\_unknown19\_PKC\_zetaceramide}} +} \quad (38)$$

6.20 Reaction unknown20

This is a reversible reaction of one reactant forming one product influenced by two modifiers.

Name unknown20

SBO:0000176 biochemical reaction

Notes

MIRIAM Annotation This biological entity is described by urn:miriam:obo.go:GO%3A0042542.

This biological entity is described by urn:miriam:obo.eco:ECO%3A0000313.

Reaction equation

$$\mathtt{Acid\_Sphingomyelinase} \xrightleftharpoons{\mathtt{TNFalphaTNFR1AFAN},\ \mathtt{TNFalphaTNFR1ATRADDMADDcIAP2RIPTRAF2RAIDD}} \mathtt{Acid\_Sphin} \quad (39)$$

Reactants

Table 62: Properties of each reactant.

Id	Name	SBO
Acid_Sphingomyelinase	unknown86	0000010

Modifiers

Table 63: Properties of each modifier.

Id	Name	SBO
TNFalphaTNFR1AFAN	mod_unknown20_1	0000460
TNFalphaTNFR1ATRADDMADDcIAP2RIPTRAF2RAIDD	mod_unknown20	0000460

Products

Table 64: Properties of each product.

Id	Name	SBO
Acid_Sphingomyelinase	unknown86	0000010

Kinetic law

SBO:0000528 common modular rate law

Derived unit $\mathrm{mol} \cdot \mathrm{s}^{-1}$

$$v_{20} = [\mathtt{TNFalphaTNFR1AFAN}] \cdot \mathrm{vol}(\mathtt{default}) \cdot \frac{\mathtt{kcrf\_unknown20\_TNFalphaTNFR1AFAN} \cdot \left(\frac{[\mathtt{Acid\_Sphingomyelinase}] \cdot \mathrm{vol}(\mathtt{default})}{\mathtt{kmc\_unknown20\_Acid\_Sphingomyelinase\_TNFalphaTNFR1AFAN}}\right)^{\mathtt{unknown86} \cdot \mathtt{hco\_unknow}\text{[illegible]}}}{\left(1 + \frac{[\mathtt{Acid\_Sphingomyelinase}] \cdot \mathrm{vol}(\mathtt{default})}{\mathtt{kmc\_unknown20\_Acid\_Sphingomyelinase\_TNFalphaTNFR1AFAN}}\right)^{\mathtt{unknown86} \cdot \mathtt{hco\_unknown20}\text{[illegible]}} \text{[illegible]}} + [\mathtt{TNFalphaTNFR1ATRADDMADDcIAP2RIPTRAF2RAIDD}] \cdot \mathrm{vol}(\mathtt{default}) \cdot \frac{\mathtt{kcrf\_unknown20\_TNFalphaTNFR1ATRADDMADDcIAP2RIPTRAF2RAIDD} \cdot \left(\frac{[\mathtt{Acid\_Sphingomyelina}\text{[illegible]}}{\mathtt{kmc\_unknown20\_Acid\_Sphingomyelinase\_TNFalp}\text{[illegible]}}\right.\text{[illegible]}}{\left(1 + \frac{[\mathtt{Acid\_Sphingomyelinase}]\text{[illegible]}}{\mathtt{kmc\_unknown20\_Acid\_Sphingomyelinase\_TNFalpha}\text{[illegible]}}\right.\text{[illegible]}} \quad (40)$$

6.21 Reaction unknown21

This is a reversible reaction of one reactant forming one product influenced by one modifier.

Name unknown21

SBO:0000176 biochemical reaction

Notes

MIRIAM Annotation This biological entity is described by urn:miriam:obo.eco:ECO%3A0000313.

Reaction equation

$$\texttt{MKK4\_1} \xrightleftharpoons{\texttt{MEKK1}} \texttt{MKK4} \tag{41}$$

Reactants

Table 65: Properties of each reactant.

Id	Name	SBO
MKK4_1	unknown91	0000010

Modifiers

Table 66: Properties of each modifier.

Id	Name	SBO
MEKK1	mod_unknown21	0000013

Products

Table 67: Properties of each product.

Id	Name	SBO
MKK4	unknown71	0000010

Kinetic law

SBO:0000528 common modular rate law

Derived unit $\mathrm{mol} \cdot \mathrm{s}^{-1}$

$$v_{21} = \frac{\texttt{vmaf\_unknown21} \cdot \left(\frac{[\texttt{MKK4\_1}] \cdot \mathrm{vol}(\texttt{cytoplasm})}{\texttt{kmc\_unknown21\_MKK4\_1}}\right)^{\texttt{unknown91} \cdot \texttt{hco\_unknown21}} - \texttt{vmar\_unknown21} \cdot \left(\frac{[\texttt{MKK4}] \cdot \mathrm{vol}(\texttt{default})}{\texttt{kmc\_unknown21\_MKK4}}\right)^{\texttt{unknown71} \cdot \text{[illegible]}}}{\left(1 + \frac{[\texttt{MKK4\_1}] \cdot \mathrm{vol}(\texttt{cytoplasm})}{\texttt{kmc\_unknown21\_MKK4\_1}}\right)^{\texttt{unknown91} \cdot \texttt{hco\_unknown21}} + \left(1 + \frac{[\texttt{MKK4}] \cdot \mathrm{vol}(\texttt{default})}{\texttt{kmc\_unknown21\_MKK4}}\right)^{\texttt{unknown71} \cdot \texttt{hco\_unknown21}} - 1} \tag{42}$$

6.22 Reaction unknown22

This is a reversible reaction of two reactants forming one product.

Name unknown22

SBO:0000176 biochemical reaction

Notes

MIRIAM Annotation This biological entity is described by urn:miriam:pubmed:10508159.

This biological entity is described by urn:miriam:obo.eco:ECO%3A0000313.

Reaction equation

$$\texttt{Cathepsin\_D} + \texttt{ceramide} \rightleftharpoons \texttt{Cathepsin\_Dceramide} \tag{43}$$

Reactants

Table 68: Properties of each reactant.

Id	Name	SBO
Cathepsin_D	unknown57	0000010
ceramide	unknown3	0000010

Products

Table 69: Properties of each product.

Id	Name	SBO
Cathepsin_Dceramide	unknown59	0000010

Kinetic law

SBO:0000528 common modular rate law

Derived unit $\mathrm{mol} \cdot \mathrm{s}^{-1}$

$$v_{22} = \frac{\texttt{vmaf\_unknown22} \cdot \left(\frac{[\texttt{Cathepsin\_D}] \cdot \mathrm{vol}(\texttt{default})}{\texttt{kmc\_unknown22\_Cathepsin\_D}}\right)^{\texttt{unknown57} \cdot \texttt{hco\_unknown22}} \cdot \left(\frac{[\texttt{ceramide}] \cdot \mathrm{vol}(\texttt{default})}{\texttt{kmc\_unknown22\_ceramide}}\right)^{\texttt{unknown3\_3} \cdot \texttt{hco\_unknown22}}}{\left(1 + \frac{[\texttt{Cathepsin\_D}] \cdot \mathrm{vol}(\texttt{default})}{\texttt{kmc\_unknown22\_Cathepsin\_D}}\right)^{\texttt{unknown57} \cdot \texttt{hco\_unknown22}} \cdot \left(1 + \frac{[\texttt{ceramide}] \cdot \mathrm{vol}(\texttt{default})}{\texttt{kmc\_unknown22\_ceramide}}\right)^{\texttt{unknown3\_3} \cdot \texttt{hco\_unknown22}}} \tag{44}$$

6.23 Reaction unknown23

This is a reversible reaction of one reactant forming one product influenced by two modifiers.

Name unknown23

SBO:0000176 biochemical reaction

Notes

MIRIAM Annotation This biological entity is described by urn:miriam:obo.eco:ECO%3A0000313.

Reaction equation

$$\texttt{sphingomyelin} \xrightleftharpoons{\texttt{EGF},\ \texttt{Acid\_Sphingomyelinase}} \texttt{ceramide} \tag{45}$$

Reactants

Table 70: Properties of each reactant.

Id	Name	SBO
sphingomyelin	unknown16	0000010

Modifiers

Table 71: Properties of each modifier.

Id	Name	SBO
EGF	mod_unknown23_1	0000013
Acid_Sphingomyelinase	mod_unknown23	0000013

Products

Table 72: Properties of each product.

Id	Name	SBO
ceramide	unknown3	0000010

Kinetic law

SBO:0000528 common modular rate law

Derived unit $\mathrm{mol \cdot s^{-1}}$

$$v_{23} = \frac{\texttt{vmaf\_unknown23} \cdot \left(\frac{[\texttt{sphingomyelin}] \cdot \mathrm{vol}(\texttt{default})}{\texttt{kmc\_unknown23\_sphingomyelin}}\right)^{\texttt{unknown16\_2} \cdot \texttt{hco\_unknown23}} - \texttt{vmar\_unknown23} \cdot \left(\frac{[\texttt{ceramide}] \cdot \mathrm{vol}(\texttt{default}\text{[illegible]}}{\texttt{kmc\_unknown23\_ceramid}\text{[illegible]}}\right.\text{[illegible]}}{\left(1 + \frac{[\texttt{sphingomyelin}] \cdot \mathrm{vol}(\texttt{default})}{\texttt{kmc\_unknown23\_sphingomyelin}}\right)^{\texttt{unknown16\_2} \cdot \texttt{hco\_unknown23}} + \left(1 + \frac{[\texttt{ceramide}] \cdot \mathrm{vol}(\texttt{default})}{\texttt{kmc\_unknown23\_ceramide}}\right)^{\texttt{unknown3\_4} \cdot \texttt{hco\_un}\text{[illegible]}}} \quad (46)$$

6.24 Reaction `unknown24`

This is a reversible reaction of one reactant forming one product.

Name unknown24

SBO:0000176 biochemical reaction

Notes

MIRIAM Annotation This biological entity is described by urn:miriam:obo.go:GO%3A0005031.

This biological entity is described by urn:miriam:obo.eco:ECO%3A0000313.

Reaction equation

$$\texttt{PP2A\_Heterotrimer} \rightleftharpoons \texttt{PP2A\_Heterotrimer} \quad (47)$$

Reactants

Table 73: Properties of each reactant.

Id	Name	SBO
PP2A_Heterotrimer	unknown111	0000010

Products

Table 74: Properties of each product.

Id	Name	SBO
PP2A_Heterotrimer	unknown111	0000010

Kinetic law

SBO:0000528 common modular rate law

Derived unit $\mathrm{mol} \cdot \mathrm{s}^{-1}$

$$v_{24} = \frac{\mathtt{vmaf\_unknown24} \cdot \left(\frac{[\mathtt{PP2A\_Heterotrimer}] \cdot \mathrm{vol}(\mathtt{mitochondria})}{\mathtt{kmc\_unknown24\_PP2A\_Heterotrimer}}\right)^{\mathtt{unknown111} \cdot \mathtt{hco\_unknown24}} - \mathtt{vmar\_unknown24} \cdot \left(\frac{[\mathtt{PP2A\_Heterotr}\text{[illegible]}}{\mathtt{kmc\_unknown2}\text{[illegible]}}\right.\text{[illegible]}}{\left(1 + \frac{[\mathtt{PP2A\_Heterotrimer}] \cdot \mathrm{vol}(\mathtt{mitochondria})}{\mathtt{kmc\_unknown24\_PP2A\_Heterotrimer}}\right)^{\mathtt{unknown111} \cdot \mathtt{hco\_unknown24}} + \left(1 + \frac{[\mathtt{PP2A\_Heterotrimer}] \cdot \mathrm{vol}(\mathtt{mitochondr}\text{[illegible]}}{\mathtt{kmc\_unknown24\_PP2A\_Heterotrimer}\text{[illegible]}}\right.\text{[illegible]}} \quad (48)$$

6.25 Reaction `unknown25_1`

This is a reversible reaction of one reactant forming one product influenced by one modifier.

Name unknown25

SBO:0000176 biochemical reaction

Notes

MIRIAM Annotation This biological entity is described by urn:miriam:obo.eco:ECO%3A0000313.

Reaction equation

$$\mathtt{AKT1} \xrightleftharpoons{\mathtt{PKC\_zetaceramide}} \mathtt{AKT1} \quad (49)$$

Reactants

Table 75: Properties of each reactant.

Id	Name	SBO
AKT1	hsa:207	0000010

Modifiers

Table 76: Properties of each modifier.

Id	Name	SBO
PKC_zetaceramide	mod_unknown25	0000460

Products

Table 77: Properties of each product.

Id	Name	SBO
AKT1	hsa:207	0000010

Kinetic law

SBO:0000528 common modular rate law

Derived unit $\mathrm{mol} \cdot \mathrm{s}^{-1}$

$$v_{25} = [\mathtt{PKC\_zetaceramide}] \cdot \mathrm{vol}(\mathtt{default}) \cdot \frac{\mathtt{kcrf\_unknown25\_1\_PKC\_zetaceramide} \cdot \left(\frac{[\mathtt{AKT1}] \cdot \mathrm{vol}(\mathtt{cytoplasm})}{\mathtt{kmc\_unknown25\_1\_AKT1\_PKC\_zetaceramide}}\right)^{\mathtt{hsa207} \cdot \mathtt{hco\_unknown25\_1\_PKC\_zetacerami}}}{\left(1 + \frac{[\mathtt{AKT1}] \cdot \mathrm{vol}(\mathtt{cytoplasm})}{\mathtt{kmc\_unknown25\_1\_AKT1\_PKC\_zetaceramide}}\right)^{\mathtt{hsa207} \cdot \mathtt{hco\_unknown25\_1\_PKC\_zetaceramide}}} \quad (50)$$

6.26 Reaction unknown26

This is a reversible reaction of one reactant forming one product influenced by one modifier.

Name unknown26

SBO:0000176 biochemical reaction

Notes

MIRIAM Annotation This biological entity is described by urn:miriam:obo.eco:ECO%3A0000313.

Reaction equation

$$\mathtt{SPHK2} \xrightleftharpoons{\mathtt{PDGFA}} \mathtt{SPHK2} \quad (51)$$

Reactants

Table 78: Properties of each reactant.

Id	Name	SBO
SPHK2	hsa:56848	0000010

Modifiers

Table 79: Properties of each modifier.

Id	Name	SBO
PDGFA	mod_unknown26	0000013

Products

Table 80: Properties of each product.

Id	Name	SBO
SPHK2	hsa:56848	0000010

Kinetic law

SBO:0000528 common modular rate law

Derived unit $\mathrm{mol}\cdot\mathrm{s}^{-1}$

$$v_{26} = \frac{\mathtt{vmaf\_unknown26}\cdot\left(\frac{[\mathtt{SPHK2}]\cdot\mathrm{vol}(\mathtt{default})}{\mathtt{kmc\_unknown26\_SPHK2}}\right)^{\mathtt{hsa56848}\cdot\mathtt{hco\_unknown26}} - \mathtt{vmar\_unknown26}\cdot\left(\frac{[\mathtt{SPHK2}]\cdot\mathrm{vol}(\mathtt{default})}{\mathtt{kmc\_unknown26\_SPHK2}}\right)^{\mathtt{hsa56848\_1}\cdot\mathtt{h}\text{[illegible]}}}{\left(1+\frac{[\mathtt{SPHK2}]\cdot\mathrm{vol}(\mathtt{default})}{\mathtt{kmc\_unknown26\_SPHK2}}\right)^{\mathtt{hsa56848}\cdot\mathtt{hco\_unknown26}} + \left(1+\frac{[\mathtt{SPHK2}]\cdot\mathrm{vol}(\mathtt{default})}{\mathtt{kmc\_unknown26\_SPHK2}}\right)^{\mathtt{hsa56848\_1}\cdot\mathtt{hco\_unknown26}} - 1} \quad (52)$$

6.27 Reaction unknown28

This is a reversible reaction of one reactant forming one product.

Name unknown28

SBO:0000176 biochemical reaction

Notes

MIRIAM Annotation This biological entity is described by urn:miriam:pubmed:8393446.

This biological entity is described by urn:miriam:obo.eco:ECO%3A0000313.

Reaction equation

$$\mathtt{PP2A\_Heterotrimer} \rightleftharpoons \mathtt{PP2A\_Heterotrimer} \quad (53)$$

Reactants

Table 81: Properties of each reactant.

Id	Name	SBO
PP2A_Heterotrimer	unknown111	0000010

Products

Table 82: Properties of each product.

Id	Name	SBO
PP2A_Heterotrimer	unknown111	0000010

Kinetic law

SBO:0000528 common modular rate law

Derived unit $\mathrm{mol \cdot s^{-1}}$

$$v_{27} = \frac{\mathtt{vmaf\_unknown28} \cdot \left(\frac{[\mathtt{PP2A\_Heterotrimer}] \cdot \mathrm{vol}(\mathtt{mitochondria})}{\mathtt{kmc\_unknown28\_PP2A\_Heterotrimer}}\right)^{\mathtt{unknown111\_2} \cdot \mathtt{hco\_unknown28}} - \mathtt{vmar\_unknown28} \cdot \left(\frac{[\mathtt{PP2A\_Heterot}}{\mathtt{kmc\_unknow}}\right.}{\left(1 + \frac{[\mathtt{PP2A\_Heterotrimer}] \cdot \mathrm{vol}(\mathtt{mitochondria})}{\mathtt{kmc\_unknown28\_PP2A\_Heterotrimer}}\right)^{\mathtt{unknown111\_2} \cdot \mathtt{hco\_unknown28}} + \left(1 + \frac{[\mathtt{PP2A\_Heterotrimer}] \cdot \mathrm{vol}(\mathtt{mitochonc}}{\mathtt{kmc\_unknown28\_PP2A\_Heterotrim}}\right.} \tag{54}$$

6.28 Reaction unknown30_1

This is a reversible reaction of one reactant forming one product influenced by one modifier.

Name unknown30

SBO:0000176 biochemical reaction

Notes

MIRIAM Annotation This biological entity is described by urn:miriam:pubmed:11148216.

This biological entity is described by urn:miriam:obo.eco:ECO%3A0000313.

Reaction equation

$$\mathtt{RAX} \xrightleftharpoons{\mathtt{ceramide}} \mathtt{RAX} \tag{55}$$

Reactants

Table 83: Properties of each reactant.

Id	Name	SBO
RAX	hsa:30062	0000010

Modifiers

Table 84: Properties of each modifier.

Id	Name	SBO
ceramide	mod_unknown30	0000013

Products

Table 85: Properties of each product.

Id	Name	SBO
RAX	hsa:30062	0000010

Kinetic law

SBO:0000528 common modular rate law

Derived unit $\mathrm{mol} \cdot \mathrm{s}^{-1}$

$$v_{28} = \frac{\mathtt{vmaf\_unknown30\_1} \cdot \left(\frac{[\mathtt{RAX}] \cdot \mathrm{vol}(\mathtt{default})}{\mathtt{kmc\_unknown30\_1\_RAX}}\right)^{\mathtt{hsa30062} \cdot \mathtt{hco\_unknown30\_1}} - \mathtt{vmar\_unknown30\_1} \cdot \left(\frac{[\mathtt{RAX}] \cdot \mathrm{vol}(\mathtt{default})}{\mathtt{kmc\_unknown30\_1\_RAX}}\right)^{\mathtt{hsa300}\text{[illegible]}}}{\left(1 + \frac{[\mathtt{RAX}] \cdot \mathrm{vol}(\mathtt{default})}{\mathtt{kmc\_unknown30\_1\_RAX}}\right)^{\mathtt{hsa30062} \cdot \mathtt{hco\_unknown30\_1}} + \left(1 + \frac{[\mathtt{RAX}] \cdot \mathrm{vol}(\mathtt{default})}{\mathtt{kmc\_unknown30\_1\_RAX}}\right)^{\mathtt{hsa30062\_1} \cdot \mathtt{hco\_unknown30\_1}} \text{[illegible]}} \quad (56)$$

6.29 Reaction unknown31

This is a reversible reaction of one reactant forming one product.

Name unknown31

SBO:0000176 biochemical reaction

Notes

MIRIAM Annotation This biological entity is described by urn:miriam:pubmed:11723139.

This biological entity is described by urn:miriam:obo.eco:ECO%3A0000313.

Reaction equation

$$\text{AKT1} \rightleftharpoons \text{AKT1} \tag{57}$$

Reactants

Table 86: Properties of each reactant.

Id	Name	SBO
AKT1	hsa:207	0000010

Products

Table 87: Properties of each product.

Id	Name	SBO
AKT1	hsa:207	0000010

Kinetic law

SBO:0000528 common modular rate law

Derived unit $\mathrm{mol \cdot s^{-1}}$

$$v_{29} = \frac{\text{vmaf\_unknown31} \cdot \left(\frac{[\text{AKT1}] \cdot \text{vol}(\text{cytoplasm})}{\text{kmc\_unknown31\_AKT1}}\right)^{\text{hsa207\_2} \cdot \text{hco\_unknown31}} - \text{vmar\_unknown31} \cdot \left(\frac{[\text{AKT1}] \cdot \text{vol}(\text{cytoplasm})}{\text{kmc\_unknown31\_AKT1}}\right)^{\text{hsa207\_3} \cdot \text{hco}}}{\left(1 + \frac{[\text{AKT1}] \cdot \text{vol}(\text{cytoplasm})}{\text{kmc\_unknown31\_AKT1}}\right)^{\text{hsa207\_2} \cdot \text{hco\_unknown31}} + \left(1 + \frac{[\text{AKT1}] \cdot \text{vol}(\text{cytoplasm})}{\text{kmc\_unknown31\_AKT1}}\right)^{\text{hsa207\_3} \cdot \text{hco\_unknown31}} - 1} \tag{58}$$

6.30 Reaction unknown32

This is a reversible reaction of three reactants forming one product.

Name unknown32

SBO:0000176 biochemical reaction

Notes

MIRIAM Annotation This biological entity is described by urn:miriam:pubmed:8808629.

This biological entity is described by urn:miriam:obo.eco:ECO%3A0000313.

Reaction equation

$$\texttt{TNFR1A\_1} + \texttt{TNFalpha\_1} + \texttt{FAN} \rightleftharpoons \texttt{TNFalphaTNFR1AFAN} \tag{59}$$

Reactants

Table 88: Properties of each reactant.

Id	Name	SBO
TNFR1A_1	unknown150	0000010
TNFalpha_1	unknown172	0000010
FAN	unknown148	0000010

Products

Table 89: Properties of each product.

Id	Name	SBO
TNFalphaTNFR1AFAN	unknown151	0000010

Kinetic law

SBO:0000528 common modular rate law

Derived unit $\mathrm{mol} \cdot \mathrm{s}^{-1}$

$$v_{30} = \frac{\texttt{vmaf\_unknown32} \cdot \left(\frac{[\texttt{TNFR1A\_1}] \cdot \mathrm{vol}(\texttt{default})}{\texttt{kmc\_unknown32\_TNFR1A\_1}}\right)^{\texttt{unknown150} \cdot \texttt{hco\_unknown32}} \cdot \left(\frac{[\texttt{TNFalpha\_1}] \cdot \mathrm{vol}(\texttt{extracellular\_region})}{\texttt{kmc\_unknown32\_TNFalpha\_1}}\right)^{\texttt{unknown172} \cdot \texttt{h}\text{[illegible]}}}{\left(1 + \frac{[\texttt{TNFR1A\_1}] \cdot \mathrm{vol}(\texttt{default})}{\texttt{kmc\_unknown32\_TNFR1A\_1}}\right)^{\texttt{unknown150} \cdot \texttt{hco\_unknown32}} \cdot \left(1 + \frac{[\texttt{TNFalpha\_1}] \cdot \mathrm{vol}(\texttt{extracellular\_region})}{\texttt{kmc\_unknown32\_TNFalpha\_1}}\right)^{\texttt{unknown172} \cdot \texttt{hco}\text{[illegible]}}} \tag{60}$$

6.31 Reaction unknown33_1

This is a reversible reaction of two reactants forming two products influenced by one modifier.

Name unknown33

SBO:0000176 biochemical reaction

Notes

MIRIAM Annotation This biological entity is described by urn:miriam:obo.eco:ECO%3A0000313.

Reaction equation

$$\mathtt{ERK2} + \mathtt{ERK1} \xrightleftharpoons{\mathtt{MEK2}} \mathtt{ERK1} + \mathtt{ERK2} \tag{61}$$

Reactants

Table 90: Properties of each reactant.

Id	Name	SBO
ERK2	unknown179	0000010
ERK1	unknown4	0000010

Modifiers

Table 91: Properties of each modifier.

Id	Name	SBO
MEK2	mod_unknown33	0000013

Products

Table 92: Properties of each product.

Id	Name	SBO
ERK1	unknown4	0000010
ERK2	unknown179	0000010

Kinetic law

SBO:0000528 common modular rate law

Derived unit $\mathrm{mol} \cdot \mathrm{s}^{-1}$

$$v_{31} = \frac{\mathtt{vmaf\_unknown33\_1} \cdot \left(\frac{[\mathtt{ERK2}] \cdot \mathrm{vol}(\mathtt{cytoplasm})}{\mathtt{kmc\_unknown33\_1\_ERK2}}\right)^{\mathtt{unknown179} \cdot \mathtt{hco\_unknown33\_1}} \cdot \left(\frac{[\mathtt{ERK1}] \cdot \mathrm{vol}(\mathtt{cytoplasm})}{\mathtt{kmc\_unknown33\_1\_ERK1}}\right)^{\mathtt{unknown4\_2} \cdot \mathtt{hco\_unknown33\_1}}}{\left(1 + \frac{[\mathtt{ERK2}] \cdot \mathrm{vol}(\mathtt{cytoplasm})}{\mathtt{kmc\_unknown33\_1\_ERK2}}\right)^{\mathtt{unknown179} \cdot \mathtt{hco\_unknown33\_1}} \cdot \left(1 + \frac{[\mathtt{ERK1}] \cdot \mathrm{vol}(\mathtt{cytoplasm})}{\mathtt{kmc\_unknown33\_1\_ERK1}}\right)^{\mathtt{unknown4\_2} \cdot \mathtt{hco\_unknown33\_1}} +} \quad (62)$$

6.32 Reaction unknown34

This is a reversible reaction of two reactants forming two products influenced by one modifier.

Name unknown34

SBO:0000176 biochemical reaction

Notes

MIRIAM Annotation This biological entity is described by urn:miriam:obo.eco:ECO%3A0000313.

Reaction equation

$$\mathtt{ERK2} + \mathtt{ERK1} \xrightleftharpoons{\mathtt{MEK1}} \mathtt{ERK1} + \mathtt{ERK2} \quad (63)$$

Reactants

Table 93: Properties of each reactant.

Id	Name	SBO
ERK2	unknown179	0000010
ERK1	unknown4	0000010

Modifiers

Table 94: Properties of each modifier.

Id	Name	SBO
MEK1	mod_unknown34	0000013

Products

Table 95: Properties of each product.

Id	Name	SBO
ERK1	unknown4	0000010
ERK2	unknown179	0000010

Kinetic law

SBO:0000528 common modular rate law

Derived unit $\mathrm{mol} \cdot \mathrm{s}^{-1}$

$$v_{32} = \frac{\mathtt{vmaf\_unknown34} \cdot \left(\frac{[\mathtt{ERK2}] \cdot \mathrm{vol}(\mathtt{cytoplasm})}{\mathtt{kmc\_unknown34\_ERK2}}\right)^{\mathtt{unknown179\_2} \cdot \mathtt{hco\_unknown34}} \cdot \left(\frac{[\mathtt{ERK1}] \cdot \mathrm{vol}(\mathtt{cytoplasm})}{\mathtt{kmc\_unknown34\_ERK1}}\right)^{\mathtt{unknown4\_4} \cdot \mathtt{hco\_unknown34}} - \mathtt{vm}}{\left(1 + \frac{[\mathtt{ERK2}] \cdot \mathrm{vol}(\mathtt{cytoplasm})}{\mathtt{kmc\_unknown34\_ERK2}}\right)^{\mathtt{unknown179\_2} \cdot \mathtt{hco\_unknown34}} \cdot \left(1 + \frac{[\mathtt{ERK1}] \cdot \mathrm{vol}(\mathtt{cytoplasm})}{\mathtt{kmc\_unknown34\_ERK1}}\right)^{\mathtt{unknown4\_4} \cdot \mathtt{hco\_unknown34}} + \left(1 +\right.} \quad (64)$$

6.33 Reaction unknown35

This is a reversible reaction of one reactant forming one product influenced by one modifier.

Name unknown35

SBO:0000176 biochemical reaction

Notes

MIRIAM Annotation This biological entity is described by urn:miriam:pubmed:10400650.

This biological entity is described by urn:miriam:obo.eco:ECO%3A0000313.

Reaction equation

$$\mathtt{BCL2} \xrightleftharpoons{\mathtt{PP2A\_Heterotrimer}} \mathtt{BCL2} \quad (65)$$

Reactants

Table 96: Properties of each reactant.

Id	Name	SBO
BCL2	hsa:596	0000010

Modifiers

Table 97: Properties of each modifier.

Id	Name	SBO
PP2A_Heterotrimer	mod_unknown35	0000460

Products

Table 98: Properties of each product.

Id	Name	SBO
BCL2	hsa:596	0000010

Kinetic law

SBO:0000528 common modular rate law

Derived unit $\mathrm{mol \cdot s^{-1}}$

$$v_{33} = [\mathtt{PP2A\_Heterotrimer}] \cdot \mathrm{vol}\left(\mathtt{mitochondria}\right) \tag{66}$$
$$\cdot \frac{\mathtt{kcrf\_unknown35\_PP2A\_Heterotrimer} \cdot \left(\frac{[\mathtt{BCL2}] \cdot \mathrm{vol}(\mathtt{default})}{\mathtt{kmc\_unknown35\_BCL2\_PP2A\_Heterotrimer}}\right)^{\mathtt{hsa596 \cdot hco\_unknown35\_PP2A\_Heterotrimer}}}{\left(1 + \frac{[\mathtt{BCL2}] \cdot \mathrm{vol}(\mathtt{default})}{\mathtt{kmc\_unknown35\_BCL2\_PP2A\_Heterotrimer}}\right)^{\mathtt{hsa596 \cdot hco\_unknown35\_PP2A\_Heterotrimer}} +}$$

6.34 Reaction unknown36

This is a reversible reaction of one reactant forming one product.

Name unknown36

SBO:0000176 biochemical reaction

Notes

MIRIAM Annotation This biological entity is described by urn:miriam:obo.eco:ECO%3A0000313.

Reaction equation

$$\mathtt{BAD} \rightleftharpoons \mathtt{BAD} \tag{67}$$

Reactants

Table 99: Properties of each reactant.

Id	Name	SBO
BAD	hsa:572	0000010

Products

Table 100: Properties of each product.

Id	Name	SBO
BAD	hsa:572	0000010

Kinetic law

SBO:0000528 common modular rate law

Derived unit $\mathrm{mol} \cdot \mathrm{s}^{-1}$

$$v_{34} = \frac{\mathtt{vmaf\_unknown36} \cdot \left(\frac{[\mathtt{BAD}] \cdot \mathrm{vol}(\mathtt{default})}{\mathtt{kmc\_unknown36\_BAD}}\right)^{\mathtt{hsa572} \cdot \mathtt{hco\_unknown36}} - \mathtt{vmar\_unknown36} \cdot \left(\frac{[\mathtt{BAD}] \cdot \mathrm{vol}(\mathtt{default})}{\mathtt{kmc\_unknown36\_BAD}}\right)^{\mathtt{hsa572\_1} \cdot \mathtt{hco\_unkno}\text{[illegible]}}}{\left(1 + \frac{[\mathtt{BAD}] \cdot \mathrm{vol}(\mathtt{default})}{\mathtt{kmc\_unknown36\_BAD}}\right)^{\mathtt{hsa572} \cdot \mathtt{hco\_unknown36}} + \left(1 + \frac{[\mathtt{BAD}] \cdot \mathrm{vol}(\mathtt{default})}{\mathtt{kmc\_unknown36\_BAD}}\right)^{\mathtt{hsa572\_1} \cdot \mathtt{hco\_unknown36}} - 1} \quad (68)$$

6.35 Reaction unknown37

This is a reversible reaction of one reactant forming one product influenced by one modifier.

Name unknown37

SBO:0000176 biochemical reaction

Notes

MIRIAM Annotation This biological entity is described by urn:miriam:obo.eco:ECO%3A0000313.

Reaction equation

$$\mathtt{BAD} \xrightleftharpoons{\mathtt{ceramide}} \mathtt{BAD} \quad (69)$$

Reactants

Table 101: Properties of each reactant.

Id	Name	SBO
BAD	hsa:572	0000010

Modifiers

Table 102: Properties of each modifier.

Id	Name	SBO
ceramide	mod_unknown37	0000013

Products

Table 103: Properties of each product.

Id	Name	SBO
BAD	hsa:572	0000010

Kinetic law

SBO:0000528 common modular rate law

Derived unit $\mathrm{mol} \cdot \mathrm{s}^{-1}$

$$v_{35} = \frac{\mathtt{vmaf\_unknown37} \cdot \left(\frac{[\mathtt{BAD}] \cdot \mathrm{vol}(\mathtt{default})}{\mathtt{kmc\_unknown37\_BAD}}\right)^{\mathtt{hsa572\_2} \cdot \mathtt{hco\_unknown37}} - \mathtt{vmar\_unknown37} \cdot \left(\frac{[\mathtt{BAD}] \cdot \mathrm{vol}(\mathtt{default})}{\mathtt{kmc\_unknown37\_BAD}}\right)^{\mathtt{hsa572\_3} \cdot \mathtt{hco\_unk}[illegible]}}{\left(1 + \frac{[\mathtt{BAD}] \cdot \mathrm{vol}(\mathtt{default})}{\mathtt{kmc\_unknown37\_BAD}}\right)^{\mathtt{hsa572\_2} \cdot \mathtt{hco\_unknown37}} + \left(1 + \frac{[\mathtt{BAD}] \cdot \mathrm{vol}(\mathtt{default})}{\mathtt{kmc\_unknown37\_BAD}}\right)^{\mathtt{hsa572\_3} \cdot \mathtt{hco\_unknown37}} - 1} \quad (70)$$

6.36 Reaction unknown38

This is a reversible reaction of one reactant forming two products influenced by one modifier.

Name unknown38

SBO:0000176 biochemical reaction

Notes

MIRIAM Annotation This biological entity is described by urn:miriam:obo.eco:ECO%3A0000313.

Reaction equation

$$\texttt{ceramide} \xrightleftharpoons{\texttt{Acid\_Ceramidase}} \texttt{sphingosine} + \texttt{Free\_Fatty\_acid} \tag{71}$$

Reactants

Table 104: Properties of each reactant.

Id	Name	SBO
ceramide	unknown3	0000010

Modifiers

Table 105: Properties of each modifier.

Id	Name	SBO
Acid_Ceramidase	mod_unknown38	0000013

Products

Table 106: Properties of each product.

Id	Name	SBO
sphingosine	unknown54	0000010
Free_Fatty_acid	unknown207	0000010

Kinetic law

SBO:0000528 common modular rate law

Derived unit $\mathrm{mol} \cdot \mathrm{s}^{-1}$

$$v_{36} = \frac{\texttt{vmaf\_unknown38} \cdot \left(\frac{[\texttt{ceramide}] \cdot \mathrm{vol}(\texttt{default})}{\texttt{kmc\_unknown38\_ceramide}}\right)^{\texttt{unknown3\_5} \cdot \texttt{hco\_unknown38}} - \texttt{vmar\_unknown38} \cdot \left(\frac{[\texttt{sphingosine}] \cdot \mathrm{vol}(\texttt{default})}{\texttt{kmc\_unknown38\_sphingosine}}\right)^{\text{[illegible]}}}{\left(1 + \frac{[\texttt{ceramide}] \cdot \mathrm{vol}(\texttt{default})}{\texttt{kmc\_unknown38\_ceramide}}\right)^{\texttt{unknown3\_5} \cdot \texttt{hco\_unknown38}} + \left(1 + \frac{[\texttt{sphingosine}] \cdot \mathrm{vol}(\texttt{default})}{\texttt{kmc\_unknown38\_sphingosine}}\right)^{\texttt{unknown54\_1} \cdot \texttt{hco\_unknow}\text{[illegible]}}} \tag{72}$$

6.37 Reaction unknown39

This is a reversible reaction of one reactant forming two products influenced by one modifier.

Name unknown39

SBO:0000176 biochemical reaction

Notes

MIRIAM Annotation This biological entity is described by urn:miriam:obo.eco:ECO%3A0000313.

Reaction equation

$$\texttt{ceramide} \xrightleftharpoons{\texttt{PDGFA}} \texttt{sphingosine} + \texttt{Free\_Fatty\_acid} \tag{73}$$

Reactants

Table 107: Properties of each reactant.

Id	Name	SBO
ceramide	unknown3	0000010

Modifiers

Table 108: Properties of each modifier.

Id	Name	SBO
PDGFA	mod_unknown39	0000013

Products

Table 109: Properties of each product.

Id	Name	SBO
sphingosine	unknown54	0000010
Free_Fatty_acid	unknown207	0000010

Kinetic law

SBO:0000528 common modular rate law

Derived unit $\mathrm{mol} \cdot \mathrm{s}^{-1}$

$$v_{37} = \frac{\texttt{vmaf\_unknown39} \cdot \left(\frac{[\texttt{ceramide}] \cdot \mathrm{vol}(\texttt{default})}{\texttt{kmc\_unknown39\_ceramide}}\right)^{\texttt{unknown3\_6} \cdot \texttt{hco\_unknown39}} - \texttt{vmar\_unknown39} \cdot \left(\frac{[\texttt{sphingosine}] \cdot \mathrm{vol}(\texttt{default})}{\texttt{kmc\_unknown39\_sphingosine}}\right)^{\text{[illegible]}}}{\left(1 + \frac{[\texttt{ceramide}] \cdot \mathrm{vol}(\texttt{default})}{\texttt{kmc\_unknown39\_ceramide}}\right)^{\texttt{unknown3\_6} \cdot \texttt{hco\_unknown39}} + \left(1 + \frac{[\texttt{sphingosine}] \cdot \mathrm{vol}(\texttt{default})}{\texttt{kmc\_unknown39\_sphingosine}}\right)^{\texttt{unknown54\_2} \cdot \texttt{hco\_unknow}\text{[illegible]}}} \quad (74)$$

6.38 Reaction unknown40

This is a reversible reaction of two reactants forming two products.

Name unknown40

SBO:0000176 biochemical reaction

Notes

MIRIAM Annotation This biological entity is described by urn:miriam:pubmed:7559390.

This biological entity is described by urn:miriam:obo.eco:ECO%3A0000313.

Reaction equation

$$\texttt{ERK2} + \texttt{ERK1} \rightleftharpoons \texttt{ERK1} + \texttt{ERK2} \quad (75)$$

Reactants

Table 110: Properties of each reactant.

Id	Name	SBO
ERK2	unknown179	0000010
ERK1	unknown4	0000010

Products

Table 111: Properties of each product.

Id	Name	SBO
ERK1	unknown4	0000010
ERK2	unknown179	0000010

Kinetic law

SBO:0000528 common modular rate law

Derived unit $\mathrm{mol} \cdot \mathrm{s}^{-1}$

$$v_{38} = \frac{\mathtt{vmaf\_unknown40} \cdot \left(\frac{[\mathtt{ERK2}] \cdot \mathrm{vol}(\mathtt{cytoplasm})}{\mathtt{kmc\_unknown40\_ERK2}}\right)^{\mathtt{unknown179\_4} \cdot \mathtt{hco\_unknown40}} \cdot \left(\frac{[\mathtt{ERK1}] \cdot \mathrm{vol}(\mathtt{cytoplasm})}{\mathtt{kmc\_unknown40\_ERK1}}\right)^{\mathtt{unknown4\_6} \cdot \mathtt{hco\_unknown40}} - \mathtt{vm}\text{[illegible]}}{\left(1 + \frac{[\mathtt{ERK2}] \cdot \mathrm{vol}(\mathtt{cytoplasm})}{\mathtt{kmc\_unknown40\_ERK2}}\right)^{\mathtt{unknown179\_4} \cdot \mathtt{hco\_unknown40}} \cdot \left(1 + \frac{[\mathtt{ERK1}] \cdot \mathrm{vol}(\mathtt{cytoplasm})}{\mathtt{kmc\_unknown40\_ERK1}}\right)^{\mathtt{unknown4\_6} \cdot \mathtt{hco\_unknown40}} + \left(1 + \text{[illegible]}\right.} \tag{76}$$

6.39 Reaction unknown41

This is a reversible reaction of two reactants forming two products.

Name unknown41

SBO:0000176 biochemical reaction

Notes

MIRIAM Annotation This biological entity is described by:

- urn:miriam:pubmed:8933152.
- urn:miriam:pubmed:10969079.

This biological entity is described by urn:miriam:obo.eco:ECO%3A0000313.

Reaction equation

$$\mathtt{MEK1\_1} + \mathtt{MEK2\_1} \rightleftharpoons \mathtt{MEK2} + \mathtt{MEK1} \tag{77}$$

Reactants

Table 112: Properties of each reactant.

Id	Name	SBO
MEK1_1	unknown232	0000010
MEK2_1	unknown233	0000010

Products

Table 113: Properties of each product.

Id	Name	SBO
MEK2	unknown178	0000010
MEK1	unknown183	0000010

Kinetic law

SBO:0000528 common modular rate law

Derived unit $\mathrm{mol} \cdot \mathrm{s}^{-1}$

$$v_{39} = \frac{\mathrm{vmaf\_unknown41} \cdot \left(\frac{[\mathrm{MEK1\_1}] \cdot \mathrm{vol}(\mathrm{cytoplasm})}{\mathrm{kmc\_unknown41\_MEK1\_1}}\right)^{\mathrm{unknown232} \cdot \mathrm{hco\_unknown41}} \cdot \left(\frac{[\mathrm{MEK2\_1}] \cdot \mathrm{vol}(\mathrm{cytoplasm})}{\mathrm{kmc\_unknown41\_MEK2\_1}}\right)^{\mathrm{unknown233} \cdot \mathrm{hco\_unknown41}} - \mathrm{v}}{\left(1 + \frac{[\mathrm{MEK1\_1}] \cdot \mathrm{vol}(\mathrm{cytoplasm})}{\mathrm{kmc\_unknown41\_MEK1\_1}}\right)^{\mathrm{unknown232} \cdot \mathrm{hco\_unknown41}} \cdot \left(1 + \frac{[\mathrm{MEK2\_1}] \cdot \mathrm{vol}(\mathrm{cytoplasm})}{\mathrm{kmc\_unknown41\_MEK2\_1}}\right)^{\mathrm{unknown233} \cdot \mathrm{hco\_unknown41}} + (1}$$ [illegible] (78)

6.40 Reaction unknown42_3

This is a reversible reaction of one reactant forming two products influenced by one modifier.

Name unknown42

SBO:0000176 biochemical reaction

Notes

MIRIAM Annotation This biological entity is described by urn:miriam:obo.eco:ECO%3A0000313.

Reaction equation

$$\mathrm{FADDCaspase\_8} \xrightleftharpoons{\mathrm{TNFalphaTNFR1ATRADDMADDcIAP2RIPTRAF2RAIDD}} \mathrm{Caspase\_8} + \mathrm{FADD} \quad (79)$$

Reactants

Table 114: Properties of each reactant.

Id	Name	SBO
FADDCaspase_8	unknown247	0000010

Modifiers

Table 115: Properties of each modifier.

Id	Name	SBO
TNFalphaTNFR1ATRADDMADDcIAP2RIPTRAF2RAIDD	mod_unknown42	0000460

Products

Table 116: Properties of each product.

Id	Name	SBO
Caspase_8	unknown42	0000010
FADD	hsa:8772	0000010

Kinetic law

SBO:0000528 common modular rate law

Derived unit $\mathrm{mol} \cdot \mathrm{s}^{-1}$

$$v_{40} = [\mathtt{TNFalphaTNFR1ATRADDMADDcIAP2RIPTRAF2RAIDD}] \cdot \mathrm{vol}\left(\mathtt{default}\right) \cdot \frac{\mathtt{kcrf\_unknown42\_3\_TNFalphaTNFR1ATRADDMADDcIAP2RIPTRAF2RAIDD} \cdot \left(\frac{[\mathtt{FADDCaspase\_8}] \cdot \mathrm{vo}\text{[illegible]}}{\mathtt{kmc\_unknown42\_3\_FADDCaspase\_8\_TNFalphaTN}\text{[illegible]}}\right.}{\left(1 + \frac{[\mathtt{FADDCaspase\_8}] \cdot \mathrm{vol}(\mathtt{def}\text{[illegible]}}{\mathtt{kmc\_unknown42\_3\_FADDCaspase\_8\_TNFalphaTNFR1A}\text{[illegible]}}\right.} \quad (80)$$

6.41 Reaction unknown43

This is a reversible reaction of one reactant forming one product influenced by one modifier.

Name unknown43

SBO:0000176 biochemical reaction

Notes

MIRIAM Annotation This biological entity is described by urn:miriam:obo.eco:ECO%3A0000313.

Reaction equation

$$\texttt{RB1} \xrightleftharpoons{\texttt{ceramide}} \texttt{RB1} \tag{81}$$

Reactants

Table 117: Properties of each reactant.

Id	Name	SBO
RB1	hsa:5925	0000010

Modifiers

Table 118: Properties of each modifier.

Id	Name	SBO
ceramide	mod_unknown43	0000013

Products

Table 119: Properties of each product.

Id	Name	SBO
RB1	hsa:5925	0000010

Kinetic law

SBO:0000528 common modular rate law

Derived unit $\mathrm{mol} \cdot \mathrm{s}^{-1}$

$$v_{41} = \frac{\texttt{vmaf\_unknown43} \cdot \left(\frac{[\texttt{RB1}] \cdot \mathrm{vol}(\texttt{default})}{\texttt{kmc\_unknown43\_RB1}}\right)^{\texttt{hsa5925} \cdot \texttt{hco\_unknown43}} - \texttt{vmar\_unknown43} \cdot \left(\frac{[\texttt{RB1}] \cdot \mathrm{vol}(\texttt{default})}{\texttt{kmc\_unknown43\_RB1}}\right)^{\texttt{hsa5925\_1} \cdot \texttt{hco\_unk}[illegible]}}{\left(1 + \frac{[\texttt{RB1}] \cdot \mathrm{vol}(\texttt{default})}{\texttt{kmc\_unknown43\_RB1}}\right)^{\texttt{hsa5925} \cdot \texttt{hco\_unknown43}} + \left(1 + \frac{[\texttt{RB1}] \cdot \mathrm{vol}(\texttt{default})}{\texttt{kmc\_unknown43\_RB1}}\right)^{\texttt{hsa5925\_1} \cdot \texttt{hco\_unknown43}} - 1} \tag{82}$$

6.42 Reaction unknown44_1

This is a reversible reaction of two reactants forming one product.

Name unknown44

SBO:0000176 biochemical reaction

Notes

MIRIAM Annotation This biological entity is described by urn:miriam:obo.eco:ECO%3A0000313.

Reaction equation

$$\mathtt{TNFR1ABAG4} + \mathtt{TNFalpha\_1} \rightleftharpoons \mathtt{TNFR1ABAG4TNFalpha} \tag{83}$$

Reactants

Table 120: Properties of each reactant.

Id	Name	SBO
TNFR1ABAG4	unknown278	0000010
TNFalpha_1	unknown172	0000010

Products

Table 121: Properties of each product.

Id	Name	SBO
TNFR1ABAG4TNFalpha	unknown282	0000010

Kinetic law

SBO:0000528 common modular rate law

Derived unit $\mathrm{mol} \cdot \mathrm{s}^{-1}$

$$v_{42} = \frac{\mathtt{vmaf\_unknown44\_1} \cdot \left(\frac{[\mathtt{TNFR1ABAG4}] \cdot \mathrm{vol}(\mathtt{default})}{\mathtt{kmc\_unknown44\_1\_TNFR1ABAG4}}\right)^{\mathtt{unknown278} \cdot \mathtt{hco\_unknown44\_1}} \cdot \left(\frac{[\mathtt{TNFalpha\_1}] \cdot \mathrm{vol}(\mathtt{extracellular\_region})}{\mathtt{kmc\_unknown44\_1\_TNFalpha\_1}}\right)^{\mathtt{unkn}\text{[illegible]}}}{\left(1 + \frac{[\mathtt{TNFR1ABAG4}] \cdot \mathrm{vol}(\mathtt{default})}{\mathtt{kmc\_unknown44\_1\_TNFR1ABAG4}}\right)^{\mathtt{unknown278} \cdot \mathtt{hco\_unknown44\_1}} \cdot \left(1 + \frac{[\mathtt{TNFalpha\_1}] \cdot \mathrm{vol}(\mathtt{extracellular\_region})}{\mathtt{kmc\_unknown44\_1\_TNFalpha\_1}}\right)^{\mathtt{unkn}\text{[illegible]}}} \tag{84}$$

6.43 Reaction unknown45_1

This is a reversible reaction of seven reactants forming two products.

Name unknown45

SBO:0000176 biochemical reaction

Notes

MIRIAM Annotation This biological entity is described by urn:miriam:obo.eco:ECO%3A0000313.

Reaction equation

$$\mathtt{RIP} + \mathtt{RAIDD} + \mathtt{TRADD} + \mathtt{TRAF2} + \mathtt{MADD} + \mathtt{cIAP2} + \mathtt{TNFR1ABAG4TNFalpha} \rightleftharpoons \mathtt{TNFalphaTNFR1ATRADDMADDcIAP2RI} \tag{85}$$

Reactants

Table 122: Properties of each reactant.

Id	Name	SBO
RIP	unknown78	0000010
RAIDD	unknown75	0000010
TRADD	hsa:8717	0000010
TRAF2	hsa:7186	0000010
MADD	hsa:8567	0000010
cIAP2	unknown74	0000010
TNFR1ABAG4TNFalpha	unknown282	0000010

Products

Table 123: Properties of each product.

Id	Name	SBO
TNFalphaTNFR1ATRADDMADDcIAP2RIPTRAF2RAIDD	unknown79	0000010
BAG4	hsa:9530	0000010

Kinetic law

SBO:0000528 common modular rate law

Derived unit $\mathrm{mol} \cdot \mathrm{s}^{-1}$

$$v_{43} = \frac{\mathtt{vmaf\_unknown45\_1} \cdot \left(\frac{[\mathtt{RIP}]\cdot \mathrm{vol}(\mathtt{cytoplasm})}{\mathtt{kmc\_unknown45\_1\_RIP}}\right)^{\mathtt{unknown78}\cdot \mathtt{hco\_unknown45\_1}} \cdot \left(\frac{[\mathtt{RAIDD}]\cdot \mathrm{vol}(\mathtt{default})}{\mathtt{kmc\_unknown45\_1\_RAIDD}}\right)^{\mathtt{unknown75}\cdot \mathtt{hco\_unknown45\_1}} \cdot}{\left(1 + \frac{[\mathtt{RIP}]\cdot \mathrm{vol}(\mathtt{cytoplasm})}{\mathtt{kmc\_unknown45\_1\_RIP}}\right)^{\mathtt{unknown78}\cdot \mathtt{hco\_unknown45\_1}} \cdot \left(1 + \frac{[\mathtt{RAIDD}]\cdot \mathrm{vol}(\mathtt{default})}{\mathtt{kmc\_unknown45\_1\_RAIDD}}\right)^{\mathtt{unknown75}\cdot \mathtt{hco\_unknown45\_1}} \cdot \left(1 + \frac{[\mathtt{TRADD}]}{\mathtt{kmc\_unkn}}\right.} \quad (86)$$

6.44 Reaction unknown46

This is a reversible reaction of one reactant forming two products.

Name unknown46

SBO:0000176 biochemical reaction

Notes

MIRIAM Annotation This biological entity is described by:

- urn:miriam:pubmed:8681377.
- urn:miriam:pubmed:8681376.

This biological entity is described by urn:miriam:obo.eco:ECO%3A0000313.

Reaction equation

$$\mathtt{FADDCaspase\_8} \rightleftharpoons \mathtt{Caspase\_8} + \mathtt{FADD} \quad (87)$$

Reactants

Table 124: Properties of each reactant.

Id	Name	SBO
FADDCaspase_8	unknown247	0000010

Products

Table 125: Properties of each product.

Id	Name	SBO
Caspase_8	unknown42	0000010
FADD	hsa:8772	0000010

Kinetic law

SBO:0000528 common modular rate law

Derived unit $\mathrm{mol \cdot s^{-1}}$

$$v_{44} = \frac{\texttt{vmaf\_unknown46} \cdot \left(\frac{[\texttt{FADDCaspase\_8}] \cdot \mathrm{vol}(\texttt{default})}{\texttt{kmc\_unknown46\_FADDCaspase\_8}}\right)^{\texttt{unknown247\_1} \cdot \texttt{hco\_unknown46}} - \texttt{vmar\_unknown46} \cdot \left(\frac{[\texttt{Caspase\_8}] \cdot \mathrm{vol}(\texttt{cytopla}\text{[illegible]}}{\texttt{kmc\_unknown46\_Caspas}\text{[illegible]}}\right.}{\left(1 + \frac{[\texttt{FADDCaspase\_8}] \cdot \mathrm{vol}(\texttt{default})}{\texttt{kmc\_unknown46\_FADDCaspase\_8}}\right)^{\texttt{unknown247\_1} \cdot \texttt{hco\_unknown46}} + \left(1 + \frac{[\texttt{Caspase\_8}] \cdot \mathrm{vol}(\texttt{cytoplasm})}{\texttt{kmc\_unknown46\_Caspase\_8}}\right)^{\texttt{unknown42\_4} \cdot \texttt{hco\_u}\text{[illegible]}}} \quad (88)$$

6.45 Reaction unknown47

This is a reversible reaction of one reactant forming two products.

Name unknown47

SBO:0000176 biochemical reaction

Notes

MIRIAM Annotation This biological entity is described by urn:miriam:obo.eco:ECO%3A0000313.

Reaction equation

$$\texttt{NF\_kappa\_B1RelAI\_kappa\_B\_alpha\_1} \rightleftharpoons \texttt{RelANF\_kappa\_B1} + \texttt{I\_kappa\_B\_alpha\_degradation\_00} \quad (89)$$

Reactants

Table 126: Properties of each reactant.

Id	Name	SBO
NF_kappa_B1RelAI_kappa_B_alpha_1	unknown70	0000010

Products

Table 127: Properties of each product.

Id	Name	SBO
RelANF_kappa_B1	unknown38	0000010

Id	Name	SBO
I_kappa_B_alpha_degradation_00	unknown303	0000010

Kinetic law

SBO:0000528 common modular rate law

Derived unit $\mathrm{mol} \cdot \mathrm{s}^{-1}$

$$v_{45} = \frac{\mathtt{vmaf\_unknown47} \cdot \left(\frac{[\mathtt{NF\_kappa\_B1RelAI\_kappa\_B\_alpha\_1}] \cdot \mathrm{vol}(\mathtt{cytoplasm})}{\mathtt{kmc\_unknown47\_NF\_kappa\_B1RelAI\_kappa\_B\_alpha\_1}}\right)^{\mathtt{unknown70\_1} \cdot \mathtt{hco\_unknown47}} - \mathtt{vmar\_unknown47} \cdot \left(\frac{[\mathtt{Re}\text{[illegible]}}{\mathtt{km}\text{[illegible]}}\right.}{\left(1 + \frac{[\mathtt{NF\_kappa\_B1RelAI\_kappa\_B\_alpha\_1}] \cdot \mathrm{vol}(\mathtt{cytoplasm})}{\mathtt{kmc\_unknown47\_NF\_kappa\_B1RelAI\_kappa\_B\_alpha\_1}}\right)^{\mathtt{unknown70\_1} \cdot \mathtt{hco\_unknown47}} + \left(1 + \frac{[\mathtt{RelANF\_kappa\_B1}] \cdot \mathrm{vol}(\mathtt{cyto}\text{[illegible]}}{\mathtt{kmc\_unknown47\_RelANF\_ka}\text{[illegible]}}\right.} \quad (90)$$

6.46 Reaction unknown48

This is a reversible reaction of one reactant forming one product.

Name unknown48

SBO:0000176 biochemical reaction

Notes

MIRIAM Annotation This biological entity is described by urn:miriam:obo.eco:ECO%3A0000313.

Reaction equation

$$\mathtt{RB1} \rightleftharpoons \mathtt{RB1} \quad (91)$$

Reactants

Table 128: Properties of each reactant.

Id	Name	SBO
RB1	hsa:5925	0000010

Products

Table 129: Properties of each product.

Id	Name	SBO
RB1	hsa:5925	0000010

Kinetic law

SBO:0000528 common modular rate law

Derived unit $\text{mol} \cdot \text{s}^{-1}$

$$v_{46} = \frac{\text{vmaf\_unknown48} \cdot \left(\frac{[\text{RB1}] \cdot \text{vol}(\text{default})}{\text{kmc\_unknown48\_RB1}}\right)^{\text{hsa5925\_2} \cdot \text{hco\_unknown48}} - \text{vmar\_unknown48} \cdot \left(\frac{[\text{RB1}] \cdot \text{vol}(\text{default})}{\text{kmc\_unknown48\_RB1}}\right)^{\text{hsa5925\_3} \cdot \text{hco\_un}[illegible]}}{\left(1 + \frac{[\text{RB1}] \cdot \text{vol}(\text{default})}{\text{kmc\_unknown48\_RB1}}\right)^{\text{hsa5925\_2} \cdot \text{hco\_unknown48}} + \left(1 + \frac{[\text{RB1}] \cdot \text{vol}(\text{default})}{\text{kmc\_unknown48\_RB1}}\right)^{\text{hsa5925\_3} \cdot \text{hco\_unknown48}} - 1} \quad (92)$$

6.47 Reaction unknown49

This is a reversible reaction of one reactant forming one product influenced by one modifier.

Name unknown49

SBO:0000176 biochemical reaction

Notes

MIRIAM Annotation This biological entity is described by urn:miriam:obo.eco:ECO%3A0000313.

Reaction equation

$$\text{PP2A\_Heterotrimer} \xrightleftharpoons{\text{OKADAIC\_ACID}} \text{PP2A\_Heterotrimer} \quad (93)$$

Reactants

Table 130: Properties of each reactant.

Id	Name	SBO
PP2A_Heterotrimer	unknown111	0000010

Modifiers

Table 131: Properties of each modifier.

Id	Name	SBO
OKADAIC_ACID	mod_unknown49	0000013

Products

Table 132: Properties of each product.

Id	Name	SBO
PP2A_Heterotrimer	unknown111	0000010

Kinetic law

SBO:0000528 common modular rate law

Derived unit $\mathrm{mol} \cdot \mathrm{s}^{-1}$

$$v_{47} = \frac{\mathrm{vmaf\_unknown49} \cdot \left(\frac{[\mathrm{PP2A\_Heterotrimer}] \cdot \mathrm{vol}(\mathrm{mitochondria})}{\mathrm{kmc\_unknown49\_PP2A\_Heterotrimer}}\right)^{\mathrm{unknown111\_4} \cdot \mathrm{hco\_unknown49}} - \mathrm{vmar\_unknown49} \cdot \left(\frac{[\mathrm{PP2A\_Heterot}\text{[illegible]}}{\mathrm{kmc\_unknow}\text{[illegible]}}\right.}{\left(1 + \frac{[\mathrm{PP2A\_Heterotrimer}] \cdot \mathrm{vol}(\mathrm{mitochondria})}{\mathrm{kmc\_unknown49\_PP2A\_Heterotrimer}}\right)^{\mathrm{unknown111\_4} \cdot \mathrm{hco\_unknown49}} + \left(1 + \frac{[\mathrm{PP2A\_Heterotrimer}] \cdot \mathrm{vol}(\mathrm{mitochond}\text{[illegible]}}{\mathrm{kmc\_unknown49\_PP2A\_Heterotrim}\text{[illegible]}}\right.} \quad (94)$$

6.48 Reaction unknown50

This is a reversible reaction of one reactant forming one product influenced by one modifier.

Name unknown50

SBO:0000176 biochemical reaction

Notes

MIRIAM Annotation This biological entity is described by urn:miriam:obo.eco:ECO%3A0000313.

Reaction equation

$$\mathrm{PP2A\_Heterotrimer} \xrightleftharpoons{\mathrm{ceramide}} \mathrm{PP2A\_Heterotrimer} \quad (95)$$

Reactants

Table 133: Properties of each reactant.

Id	Name	SBO
PP2A_Heterotrimer	unknown111	0000010

Modifiers

Table 134: Properties of each modifier.

Id	Name	SBO
ceramide	mod_unknown50	0000013

Products

Table 135: Properties of each product.

Id	Name	SBO
PP2A_Heterotrimer	unknown111	0000010

Kinetic law

SBO:0000528 common modular rate law

Derived unit $\mathrm{mol} \cdot \mathrm{s}^{-1}$

$$v_{48} = \frac{\mathrm{vmaf\_unknown50} \cdot \left(\frac{[\mathrm{PP2A\_Heterotrimer}] \cdot \mathrm{vol}(\mathrm{mitochondria})}{\mathrm{kmc\_unknown50\_PP2A\_Heterotrimer}}\right)^{\mathrm{unknown111\_6} \cdot \mathrm{hco\_unknown50}} - \mathrm{vmar\_unknown50} \cdot \left(\frac{[\mathrm{PP2A\_Heterot}\text{[illegible]}}{\mathrm{kmc\_unknow}\text{[illegible]}}\right.}{\left(1 + \frac{[\mathrm{PP2A\_Heterotrimer}] \cdot \mathrm{vol}(\mathrm{mitochondria})}{\mathrm{kmc\_unknown50\_PP2A\_Heterotrimer}}\right)^{\mathrm{unknown111\_6} \cdot \mathrm{hco\_unknown50}} + \left(1 + \frac{[\mathrm{PP2A\_Heterotrimer}] \cdot \mathrm{vol}(\mathrm{mitochond}\text{[illegible]}}{\mathrm{kmc\_unknown50\_PP2A\_Heterotrim}\text{[illegible]}}\right.} \quad (96)$$

6.49 Reaction unknown51

This is a reversible reaction of two reactants forming two products influenced by one modifier.

Name unknown51

SBO:0000176 biochemical reaction

Notes

MIRIAM Annotation This biological entity is described by urn:miriam:obo.eco:ECO%3A0000313.

Reaction equation

$$\mathtt{MEK1\_1} + \mathtt{MEK2\_1} \xrightleftharpoons{\mathtt{RAF1}} \mathtt{MEK2} + \mathtt{MEK1} \tag{97}$$

Reactants

Table 136: Properties of each reactant.

Id	Name	SBO
MEK1_1	unknown232	0000010
MEK2_1	unknown233	0000010

Modifiers

Table 137: Properties of each modifier.

Id	Name	SBO
RAF1	mod_unknown51	0000013

Products

Table 138: Properties of each product.

Id	Name	SBO
MEK2	unknown178	0000010
MEK1	unknown183	0000010

Kinetic law

SBO:0000528 common modular rate law

Derived unit $\mathrm{mol} \cdot \mathrm{s}^{-1}$

$$v_{49} = \frac{\mathtt{vmaf\_unknown51} \cdot \left(\frac{[\mathtt{MEK1\_1}] \cdot \mathrm{vol}(\mathtt{cytoplasm})}{\mathtt{kmc\_unknown51\_MEK1\_1}}\right)^{\mathtt{unknown232\_1} \cdot \mathtt{hco\_unknown51}} \cdot \left(\frac{[\mathtt{MEK2\_1}] \cdot \mathrm{vol}(\mathtt{cytoplasm})}{\mathtt{kmc\_unknown51\_MEK2\_1}}\right)^{\mathtt{unknown233\_1} \cdot \mathtt{hco\_unknown51}}}{\left(1 + \frac{[\mathtt{MEK1\_1}] \cdot \mathrm{vol}(\mathtt{cytoplasm})}{\mathtt{kmc\_unknown51\_MEK1\_1}}\right)^{\mathtt{unknown232\_1} \cdot \mathtt{hco\_unknown51}} \cdot \left(1 + \frac{[\mathtt{MEK2\_1}] \cdot \mathrm{vol}(\mathtt{cytoplasm})}{\mathtt{kmc\_unknown51\_MEK2\_1}}\right)^{\mathtt{unknown233\_1} \cdot \mathtt{hco\_unknown51}} +} \tag{98}$$

6.50 Reaction unknown53

This is a reversible reaction of one reactant forming two products.

Name unknown53

SBO:0000176 biochemical reaction

Notes

MIRIAM Annotation This biological entity is described by urn:miriam:obo.eco:ECO%3A0000313.

Reaction equation

$$\text{ceramide} \rightleftharpoons \text{sphingosine} + \text{Free\_Fatty\_acid} \tag{99}$$

Reactants

Table 139: Properties of each reactant.

Id	Name	SBO
ceramide	unknown3	0000010

Products

Table 140: Properties of each product.

Id	Name	SBO
sphingosine	unknown54	0000010
Free_Fatty_acid	unknown207	0000010

Kinetic law

SBO:0000528 common modular rate law

Derived unit $\text{mol} \cdot \text{s}^{-1}$

$$v_{50} = \frac{\text{vmaf\_unknown53} \cdot \left(\frac{[\text{ceramide}] \cdot \text{vol}(\text{default})}{\text{kmc\_unknown53\_ceramide}}\right)^{\text{unknown3\_7} \cdot \text{hco\_unknown53}} - \text{vmar\_unknown53} \cdot \left(\frac{[\text{sphingosine}] \cdot \text{vol}(\text{default})}{\text{kmc\_unknown53\_sphingosine}}\right)^{[illegible]} [illegible]}{\left(1 + \frac{[\text{ceramide}] \cdot \text{vol}(\text{default})}{\text{kmc\_unknown53\_ceramide}}\right)^{\text{unknown3\_7} \cdot \text{hco\_unknown53}} + \left(1 + \frac{[\text{sphingosine}] \cdot \text{vol}(\text{default})}{\text{kmc\_unknown53\_sphingosine}}\right)^{\text{unknown54\_3} \cdot \text{hco\_unknow}[illegible]} [illegible]} \tag{100}$$

7 Derived Rate Equations

When interpreted as an ordinary differential equation framework, this model implies the following set of equations for the rate of change of the following species.

7.1 Species RAX

Name RAX

SBO:0000354 informational molecule segment

Notes **Description for ?RAX, MCOP3, RX?:** (RefSeq) retina and anterior neural fold homeobox

MIRIAM Annotation This biological entity is urn:miriam:entrez.gene:30062.

This biological entity is urn:miriam:kegg.genes:hsa%3A30062.

This biological entity is urn:miriam:hgnc:HGNC%3A18662.

This biological entity has an unknown relationship with:

- urn:miriam:uniprot:O75569.
- urn:miriam:uniprot:Q9Y2V3.

This biological entity has property urn:miriam:omim:601881.

This biological entity is urn:miriam:ensembl:ENSG00000134438.

This biological entity is described by urn:miriam:obo.eco:ECO%3A0000313.

Initial amount 1 mol

This species takes part in three reactions (as a reactant in unknown30_1 and as a product in unknown30_1 and as a modifier in unknown1_2).

$$\frac{\mathrm{d}}{\mathrm{d}t}\mathtt{RAX} = v_{28} - v_{28} \tag{101}$$

7.2 Species PKR

Name PKR

SBO:0000354 informational molecule segment

Notes

MIRIAM Annotation This biological entity has an unknown relationship with urn:miriam:uniprot:P19525.

This biological entity is described by urn:miriam:obo.eco:ECO%3A0000313.

Initial amount 1 mol

This species takes part in three reactions (as a reactant in unknown1_2 and as a product in unknown1_2 and as a modifier in unknown7).

$$\frac{d}{dt}\texttt{PKR} = v_1 - v_1 \tag{102}$$

7.3 Species ceramide

Name ceramide

SBO:0000247 simple chemical

Notes

MIRIAM Annotation This biological entity is urn:miriam:cas:24696-26-2.

This biological entity is described by urn:miriam:obo.eco:ECO%3A0000313.

Initial amount 1 mol

This species takes part in fifteen reactions (as a reactant in unknown8_1, unknown22, unknown38, unknown39, unknown53 and as a product in unknown6, unknown23 and as a modifier in unknown2, unknown3, unknown12, unknown16_1, unknown30_1, unknown37, unknown43, unknown50).

$$\frac{d}{dt}\texttt{ceramide} = v_6 + v_{23} - v_8 - v_{22} - v_{36} - v_{37} - v_{50} \tag{103}$$

7.4 Species ERK1

Name ERK1

SBO:0000354 informational molecule segment

Notes

MIRIAM Annotation This biological entity has an unknown relationship with urn:miriam:uniprot:P27361.

This biological entity is described by urn:miriam:obo.eco:ECO%3A0000313.

Initial amount 1 mol

This species takes part in seven reactions (as a reactant in unknown2, unknown33_1, unknown34, unknown40 and as a product in unknown33_1, unknown34, unknown40).

$$\frac{d}{dt}\texttt{ERK1} = v_{31} + v_{32} + v_{38} - v_2 - v_{31} - v_{32} - v_{38} \tag{104}$$

7.5 Species PKC_delta

Name PKC_delta

SBO:0000354 informational molecule segment

Notes

MIRIAM Annotation This biological entity has an unknown relationship with urn:miriam:uniprot:Q05655.

This biological entity is described by urn:miriam:obo.eco:ECO%3A0000313.

Initial amount 1 mol

This species takes part in one reaction (as a reactant in unknown2).

$$\frac{\mathrm{d}}{\mathrm{d}t}\texttt{PKC\_delta} = -v_2 \tag{105}$$

7.6 Species KSR

Name KSR

SBO:0000354 informational molecule segment

Notes

MIRIAM Annotation This biological entity has an unknown relationship with urn:miriam:uniprot:Q8IVT5.

This biological entity is described by urn:miriam:obo.eco:ECO%3A0000313.

Initial amount 1 mol

This species takes part in three reactions (as a reactant in unknown3 and as a product in unknown3 and as a modifier in unknown4_1).

$$\frac{\mathrm{d}}{\mathrm{d}t}\texttt{KSR} = v_3 - v_3 \tag{106}$$

7.7 Species RAF1

Name RAF1

SBO:0000354 informational molecule segment

Notes **Description for ?RAF1, CMD1NN, CRAF, NS5, Raf-1, c-Raf?:** (RefSeq) Raf-1 proto-oncogene, serine/threonine kinase (EC:2.7.11.1)

MIRIAM Annotation This biological entity has property urn:miriam:ec-code:2.7.11.1.

This biological entity is urn:miriam:entrez.gene:5894.

This biological entity is urn:miriam:kegg.genes:hsa%3A5894.

This biological entity is urn:miriam:hgnc:HGNC%3A9829.

This biological entity has an unknown relationship with:

- urn:miriam:uniprot:P04049.
- urn:miriam:uniprot:L7RRS6.

This biological entity has property urn:miriam:omim:164760.

This biological entity is urn:miriam:ensembl:ENSG00000132155.

This biological entity is described by urn:miriam:obo.eco:ECO%3A0000313.

Initial amount 1 mol

This species takes part in three reactions (as a reactant in unknown4_1 and as a product in unknown4_1 and as a modifier in unknown51).

$$\frac{\mathrm{d}}{\mathrm{d}t}\mathtt{RAF1} = v_{4} - v_{4} \tag{107}$$

7.8 Species BCL2

Name BCL2

SBO:0000354 informational molecule segment

Notes **Description for ?BCL2, Bcl-2, PPP1R50?:** (RefSeq) B-cell CLL/lymphoma 2

MIRIAM Annotation This biological entity is urn:miriam:entrez.gene:596.

This biological entity is urn:miriam:kegg.genes:hsa%3A596.

This biological entity is urn:miriam:hgnc:HGNC%3A990.

This biological entity has an unknown relationship with urn:miriam:uniprot:P10415.

This biological entity has property urn:miriam:omim:151430.

This biological entity is described by urn:miriam:obo.eco:ECO%3A0000313.

Initial amount 1 mol

This species takes part in four reactions (as a reactant in unknown35 and as a product in unknown35 and as a modifier in unknown5_1, unknown11).

$$\frac{\mathrm{d}}{\mathrm{d}t}\mathtt{BCL2} = v_{33} - v_{33} \tag{108}$$

7.9 Species Cytochrome_C

Name Cytochrome_C

SBO:0000354 informational molecule segment

Notes

MIRIAM Annotation This biological entity has an unknown relationship with urn:miriam:uniprot:P99999.

This biological entity is described by urn:miriam:obo.eco:ECO%3A0000313.

Initial amount 1 mol

This species takes part in one reaction (as a reactant in unknown5_1).

$$\frac{d}{dt}\texttt{Cytochrome\_C} = -v_5 \tag{109}$$

7.10 Species Cytochrome_C_1

Name Cytochrome_C

SBO:0000354 informational molecule segment

Notes

MIRIAM Annotation This biological entity has an unknown relationship with urn:miriam:uniprot:P99999.

This biological entity is described by urn:miriam:obo.eco:ECO%3A0000313.

Initial amount 1 mol

This species takes part in one reaction (as a product in unknown5_1).

$$\frac{d}{dt}\texttt{Cytochrome\_C\_1} = v_5 \tag{110}$$

7.11 Species Neutral_sphingomyelinase_II

Name Neutral_sphingomyelinase_II

SBO:0000354 informational molecule segment

Notes

MIRIAM Annotation This biological entity has an unknown relationship with urn:miriam:uniprot:Q9NY59.

This biological entity is described by urn:miriam:obo.eco:ECO%3A0000313.

Initial amount 1 mol

This species takes part in three reactions (as a reactant in unknown9 and as a product in unknown9 and as a modifier in unknown6).

$$\frac{d}{dt}\texttt{Neutral\_sphingomyelinase\_II} = v_9 - v_9 \tag{111}$$

7.12 Species sphingomyelin

Name sphingomyelin

SBO:0000247 simple chemical

Notes

MIRIAM Annotation This biological entity is urn:miriam:cas:9031-54-3.

This biological entity is described by urn:miriam:obo.eco:ECO%3A0000313.

Initial amount 1 mol

This species takes part in two reactions (as a reactant in unknown6, unknown23).

$$\frac{d}{dt}\texttt{sphingomyelin} = -v_6 - v_{23} \tag{112}$$

7.13 Species PHOSPHOCHOLINE

Name PHOSPHOCHOLINE

SBO:0000247 simple chemical

Notes

MIRIAM Annotation This biological entity is described by urn:miriam:obo.eco:ECO%3A0000313.

Initial amount 1 mol

This species takes part in one reaction (as a product in unknown6).

$$\frac{d}{dt}\texttt{PHOSPHOCHOLINE} = v_6 \tag{113}$$

7.14 Species EIF2A

Name EIF2A

SBO:0000354 informational molecule segment

Notes **Description for ?EIF2A, EIF-2A, MST089, MSTP004, MSTP089?:** (RefSeq) eukaryotic translation initiation factor 2A, 65kDa

MIRIAM Annotation This biological entity is urn:miriam:entrez.gene:83939.

This biological entity is urn:miriam:kegg.genes:hsa%3A83939.

This biological entity is urn:miriam:hgnc:HGNC%3A3254.

This biological entity has an unknown relationship with:

- urn:miriam:uniprot:Q9BY44.
- urn:miriam:uniprot:Q8NFM1.

This biological entity has property urn:miriam:omim:609234.

This biological entity is described by urn:miriam:obo.eco:ECO%3A0000313.

Initial amount 1 mol

This species takes part in two reactions (as a reactant in unknown7 and as a product in unknown7).

$$\frac{\mathrm{d}}{\mathrm{d}t}\texttt{EIF2A} = v_7 - v_7 \tag{114}$$

7.15 Species PAR4

Name PAR4

SBO:0000354 informational molecule segment

Notes

MIRIAM Annotation This biological entity has an unknown relationship with urn:miriam:uniprot:Q96IZ0.

This biological entity is described by urn:miriam:obo.eco:ECO%3A0000313.

Initial amount 1 mol

This species takes part in one reaction (as a reactant in unknown8_1).

$$\frac{\mathrm{d}}{\mathrm{d}t}\texttt{PAR4} = -v_8 \tag{115}$$

7.16 Species PKC_zeta

Name PKC_zeta

SBO:0000354 informational molecule segment

Notes

MIRIAM Annotation This biological entity has an unknown relationship with urn:miriam:uniprot:Q05513.

This biological entity is described by urn:miriam:obo.eco:ECO%3A0000313.

Initial amount 1 mol

This species takes part in one reaction (as a reactant in unknown8_1).

$$\frac{\mathrm{d}}{\mathrm{d}t}\texttt{PKC\_zeta} = -v_8 \tag{116}$$

7.17 Species response_to_oxidative_stress

Name response to oxidative stress

SBO:0000552 reference annotation

Notes

MIRIAM Annotation This biological entity is described by urn:miriam:obo.eco:ECO%3A0000313.

Initial amount 1 mol

This species does not take part in any reactions. Its quantity does hence not change over time:

$$\frac{\mathrm{d}}{\mathrm{d}t}\texttt{response\_to\_oxidative\_stress} = 0 \tag{117}$$

7.18 Species NF_kappa_B1

Name NF_kappa_B1

SBO:0000354 informational molecule segment

Notes

MIRIAM Annotation This biological entity has an unknown relationship with urn:miriam:uniprot:P19838.

This biological entity is described by urn:miriam:obo.eco:ECO%3A0000313.

Initial amount 1 mol

This species does not take part in any reactions. Its quantity does hence not change over time:

$$\frac{\mathrm{d}}{\mathrm{d}t}\texttt{NF\_kappa\_B1} = 0 \tag{118}$$

7.19 Species RELA

Name RELA

SBO:0000354 informational molecule segment

Notes **Description for ?RELA, NFKB3, p65?:** (RefSeq) v-rel avian reticuloendotheliosis viral oncogene homolog A

MIRIAM Annotation This biological entity is urn:miriam:entrez.gene:5970.

This biological entity is urn:miriam:kegg.genes:hsa%3A5970.

This biological entity is urn:miriam:hgnc:HGNC%3A9955.

This biological entity has an unknown relationship with urn:miriam:uniprot:Q04206.

This biological entity has property urn:miriam:omim:164014.

This biological entity is urn:miriam:ensembl:ENSG00000173039.

This biological entity is described by urn:miriam:obo.eco:ECO%3A0000313.

Initial amount 1 mol

This species does not take part in any reactions. Its quantity does hence not change over time:

$$\frac{\mathrm{d}}{\mathrm{d}t}\mathtt{RELA} = 0 \tag{119}$$

7.20 Species regulation_of_nitric_oxide_biosynthetic_process

Name regulation_of_nitric_oxide_biosynthetic_process

SBO:0000552 reference annotation

Notes

MIRIAM Annotation This biological entity is described by urn:miriam:obo.eco:ECO%3A0000313.

Initial amount 1 mol

This species does not take part in any reactions. Its quantity does hence not change over time:

$$\frac{\mathrm{d}}{\mathrm{d}t}\mathtt{regulation\_of\_nitric\_oxide\_biosynthetic\_process} = 0 \tag{120}$$

7.21 Species Caspase_8

Name Caspase_8

SBO:0000354 informational molecule segment

Notes

MIRIAM Annotation This biological entity has an unknown relationship with urn:miriam:uniprot:Q14790.

This biological entity is described by urn:miriam:obo.eco:ECO%3A0000313.

Initial amount 1 mol

This species takes part in four reactions (as a reactant in unknown10_2 and as a product in unknown10_2, unknown42_3, unknown46).

$$\frac{\mathrm{d}}{\mathrm{d}t}\texttt{Caspase\_8} = v_{10} + v_{40} + v_{44} - v_{10} \tag{121}$$

7.22 Species AIF

Name AIF

SBO:0000354 informational molecule segment

Notes

MIRIAM Annotation This biological entity has an unknown relationship with urn:miriam:uniprot:O95831.

This biological entity is described by urn:miriam:obo.eco:ECO%3A0000313.

Initial amount 1 mol

This species takes part in one reaction (as a reactant in unknown11).

$$\frac{\mathrm{d}}{\mathrm{d}t}\texttt{AIF} = -v_{11} \tag{122}$$

7.23 Species AIF_1

Name AIF

SBO:0000354 informational molecule segment

Notes

MIRIAM Annotation This biological entity has an unknown relationship with urn:miriam:uniprot:O95831.

This biological entity is described by urn:miriam:obo.eco:ECO%3A0000313.

Initial amount 1 mol

This species takes part in one reaction (as a product in unknown11).

$$\frac{\mathrm{d}}{\mathrm{d}t}\texttt{AIF\_1} = v_{11} \tag{123}$$

7.24 Species cell_survival

Name cell_survival

SBO:0000552 reference annotation

Notes

MIRIAM Annotation This biological entity is described by urn:miriam:obo.eco:ECO%3A0000313.

Initial amount 1 mol

This species does not take part in any reactions. Its quantity does hence not change over time:

$$\frac{\mathrm{d}}{\mathrm{d}t}\texttt{cell\_survival} = 0 \tag{124}$$

7.25 Species cell_proliferation

Name cell_proliferation

SBO:0000552 reference annotation

Notes

MIRIAM Annotation This biological entity is described by urn:miriam:obo.eco:ECO%3A0000313.

Initial amount 1 mol

This species does not take part in any reactions. Its quantity does hence not change over time:

$$\frac{\mathrm{d}}{\mathrm{d}t}\texttt{cell\_proliferation} = 0 \tag{125}$$

7.26 Species BAX

Name BAX

SBO:0000354 informational molecule segment

Notes **Description for ?BAX, BCL2L4?:** (RefSeq) BCL2-associated X protein

MIRIAM Annotation This biological entity is urn:miriam:entrez.gene:581.

This biological entity is urn:miriam:kegg.genes:hsa%3A581.

This biological entity is urn:miriam:hgnc:HGNC%3A959.

This biological entity has an unknown relationship with urn:miriam:uniprot:Q07812.

This biological entity has property urn:miriam:omim:600040.

This biological entity is urn:miriam:ensembl:ENSG00000087088.

This biological entity is described by urn:miriam:obo.eco:ECO%3A0000313.

Initial amount 1 mol

This species takes part in four reactions (as a reactant in unknown12 and as a product in unknown12 and as a modifier in unknown5_1, unknown11).

$$\frac{\mathrm{d}}{\mathrm{d}t}\mathtt{BAX} = v_{12} - v_{12} \tag{126}$$

7.27 Species SPHK2

Name SPHK2

SBO:0000354 informational molecule segment

Notes **Description for ?SPHK2, SK_2, SK-2, SPK_2, SPK-2?:** (RefSeq) sphingosine kinase 2 (EC:2.7.1.91)

MIRIAM Annotation This biological entity has property urn:miriam:ec-code:2.7.1.91.

This biological entity is urn:miriam:entrez.gene:56848.

This biological entity is urn:miriam:kegg.genes:hsa%3A56848.

This biological entity is urn:miriam:hgnc:HGNC%3A18859.

This biological entity has an unknown relationship with:

- urn:miriam:uniprot:Q9NRA0.
- urn:miriam:uniprot:B3KV83.

This biological entity has property urn:miriam:omim:607092.

This biological entity is urn:miriam:ensembl:ENSG00000063176.

This biological entity is described by urn:miriam:obo.eco:ECO%3A0000313.

Initial amount 1 mol

This species takes part in three reactions (as a reactant in unknown26 and as a product in unknown26 and as a modifier in unknown13_1).

$$\frac{d}{dt}\texttt{SPHK2} = v_{26} - v_{26} \tag{127}$$

7.28 Species sphingosine

Name sphingosine

SBO:0000247 simple chemical

Notes

MIRIAM Annotation This biological entity is urn:miriam:cas:123-78-4.

This biological entity is described by urn:miriam:obo.eco:ECO%3A0000313.

Initial amount 1 mol

This species takes part in four reactions (as a reactant in unknown13_1 and as a product in unknown38, unknown39, unknown53).

$$\frac{d}{dt}\texttt{sphingosine} = v_{36} + v_{37} + v_{50} - v_{13} \tag{128}$$

7.29 Species Sphingosine1phosphate

Name Sphingosine-1-phosphate

SBO:0000247 simple chemical

Notes

MIRIAM Annotation This biological entity is described by urn:miriam:obo.eco:ECO%3A0000313.

Initial amount 1 mol

This species takes part in two reactions (as a product in unknown13_1 and as a modifier in unknown6).

$$\frac{d}{dt}\texttt{Sphingosine1phosphate} = v_{13} \tag{129}$$

7.30 Species GD3

Name GD3

SBO:0000247 simple chemical

Notes

MIRIAM Annotation This biological entity is urn:miriam:cas:62010-37-1.

This biological entity is described by urn:miriam:obo.eco:ECO%3A0000313.

Initial amount 1 mol

This species takes part in one reaction (as a modifier in unknown12).

$$\frac{d}{dt}\texttt{GD3} = 0 \tag{130}$$

7.31 Species Cathepsin_D

Name Cathepsin_D

SBO:0000354 informational molecule segment

Notes

MIRIAM Annotation This biological entity has an unknown relationship with urn:miriam:uniprot:P07339.

This biological entity is described by urn:miriam:obo.eco:ECO%3A0000313.

Initial amount 1 mol

This species takes part in one reaction (as a reactant in unknown22).

$$\frac{d}{dt}\texttt{Cathepsin\_D} = -v_{22} \tag{131}$$

7.32 Species BID

Name BID

SBO:0000354 informational molecule segment

Notes **Description for ?BID, FP497?:** (RefSeq) BH3 interacting domain death agonist

MIRIAM Annotation This biological entity is urn:miriam:entrez.gene:637.

This biological entity is urn:miriam:kegg.genes:hsa%3A637.

This biological entity is urn:miriam:hgnc:HGNC%3A1050.

This biological entity has an unknown relationship with:

- urn:miriam:uniprot:A8ASI8.
- urn:miriam:uniprot:B3KT21.
- urn:miriam:uniprot:P55957.

This biological entity has property urn:miriam:omim:601997.

This biological entity is urn:miriam:ensembl:ENSG00000015475.

This biological entity is described by urn:miriam:obo.eco:ECO%3A0000313.

Initial amount 1 mol

This species takes part in two reactions (as a reactant in unknown14_1 and as a product in unknown14-_1).

$$\frac{\mathrm{d}}{\mathrm{d}t}\mathtt{BID} = v_{14} - v_{14} \tag{132}$$

7.33 Species MAP4K4

Name MAP4K4

SBO:0000354 informational molecule segment

Notes **Description for ?MAP4K4, FLH21957, HEL-S-31, HGK, MEKKK4, NIK?:** (RefSeq) mitogen-activated protein kinase kinase kinase kinase 4 (EC:2.7.11.1)

MIRIAM Annotation This biological entity has property urn:miriam:ec-code:2.7.11.1.

This biological entity is urn:miriam:entrez.gene:9448.

This biological entity is urn:miriam:kegg.genes:hsa%3A9448.

This biological entity is urn:miriam:hgnc:HGNC%3A6866.

This biological entity has an unknown relationship with urn:miriam:uniprot:O95819.

This biological entity has property urn:miriam:omim:604666.

This biological entity is urn:miriam:ensembl:ENSG00000071054.

This biological entity is described by urn:miriam:obo.eco:ECO%3A0000313.

Initial amount 1 mol

This species takes part in three reactions (as a reactant in unknown15_2 and as a product in unknown15_2 and as a modifier in unknown17).

$$\frac{\mathrm{d}}{\mathrm{d}t}\texttt{MAP4K4} = v_{15} - v_{15} \tag{133}$$

7.34 Species MEKK1

Name MEKK1

SBO:0000354 informational molecule segment

Notes

MIRIAM Annotation This biological entity has an unknown relationship with urn:miriam:uniprot:Q13233.

This biological entity is described by urn:miriam:obo.eco:ECO%3A0000313.

Initial amount 1 mol

This species takes part in three reactions (as a reactant in unknown16_1 and as a product in unknown16_1 and as a modifier in unknown21).

$$\frac{\mathrm{d}}{\mathrm{d}t}\texttt{MEKK1} = v_{16} - v_{16} \tag{134}$$

7.35 Species IKKs

Name IKKs

SBO:0000354 informational molecule segment

Notes

MIRIAM Annotation This biological entity is described by urn:miriam:obo.eco:ECO%3A0000313.

Initial amount 1 mol

This species takes part in three reactions (as a reactant in unknown17 and as a product in unknown17 and as a modifier in unknown18_1).

$$\frac{\mathrm{d}}{\mathrm{d}t}\texttt{IKKs} = v_{17} - v_{17} \tag{135}$$

7.36 Species I_kappa_B_alpha

Name I_kappa_B_alpha

SBO:0000354 informational molecule segment

Notes

MIRIAM Annotation This biological entity has an unknown relationship with urn:miriam:uniprot:P25963.

This biological entity is described by urn:miriam:obo.eco:ECO%3A0000313.

Initial amount 1 mol

This species does not take part in any reactions. Its quantity does hence not change over time:

$$\frac{\mathrm{d}}{\mathrm{d}t}\mathtt{I\_kappa\_B\_alpha} = 0 \tag{136}$$

7.37 Species MKK4

Name MKK4

SBO:0000354 informational molecule segment

Notes

MIRIAM Annotation This biological entity has an unknown relationship with urn:miriam:uniprot:P45985.

This biological entity is described by urn:miriam:obo.eco:ECO%3A0000313.

Initial amount 1 mol

This species takes part in two reactions (as a product in unknown21 and as a modifier in unknown19).

$$\frac{\mathrm{d}}{\mathrm{d}t}\mathtt{MKK4} = v_{21} \tag{137}$$

7.38 Species JNK1

Name JNK1

SBO:0000354 informational molecule segment

Notes

MIRIAM Annotation This biological entity has an unknown relationship with urn:miriam:uniprot:P45983.

This biological entity is described by urn:miriam:obo.eco:ECO%3A0000313.

Initial amount 1 mol

This species takes part in one reaction (as a reactant in unknown19).

$$\frac{d}{dt}\texttt{JNK1} = -v_{19} \tag{138}$$

7.39 Species JNK1_1

Name JNK1

SBO:0000354 informational molecule segment

Notes

MIRIAM Annotation This biological entity has an unknown relationship with urn:miriam:uniprot:P45983.

This biological entity is described by urn:miriam:obo.eco:ECO%3A0000313.

Initial amount 1 mol

This species takes part in one reaction (as a product in unknown19).

$$\frac{d}{dt}\texttt{JNK1\_1} = v_{19} \tag{139}$$

7.40 Species cIAP2

Name cIAP2

SBO:0000354 informational molecule segment

Notes

MIRIAM Annotation This biological entity has an unknown relationship with urn:miriam:uniprot:Q13489.

This biological entity is described by urn:miriam:obo.eco:ECO%3A0000313.

Initial amount 1 mol

This species takes part in one reaction (as a reactant in unknown45_1).

$$\frac{d}{dt}\texttt{cIAP2} = -v_{43} \tag{140}$$

7.41 Species RAIDD

Name RAIDD

SBO:0000354 informational molecule segment

Notes

MIRIAM Annotation This biological entity has an unknown relationship with urn:miriam:uniprot:P78560.

This biological entity is described by urn:miriam:obo.eco:ECO%3A0000313.

Initial amount 1 mol

This species takes part in one reaction (as a reactant in unknown45_1).

$$\frac{\mathrm{d}}{\mathrm{d}t}\texttt{RAIDD} = -v_{43} \tag{141}$$

7.42 Species TNFalpha

Name TNF-alpha

SBO:0000354 informational molecule segment

Notes

MIRIAM Annotation This biological entity has an unknown relationship with urn:miriam:uniprot:P01375.

This biological entity is described by urn:miriam:obo.eco:ECO%3A0000313.

Initial amount 1 mol

This species does not take part in any reactions. Its quantity does hence not change over time:

$$\frac{\mathrm{d}}{\mathrm{d}t}\texttt{TNFalpha} = 0 \tag{142}$$

7.43 Species TNFR1A

Name TNFR1A

SBO:0000354 informational molecule segment

Notes

MIRIAM Annotation This biological entity has an unknown relationship with urn:miriam:uniprot:P19438.

This biological entity is described by urn:miriam:obo.eco:ECO%3A0000313.

Initial amount 1 mol

This species does not take part in any reactions. Its quantity does hence not change over time:

$$\frac{d}{dt}\texttt{TNFR1A} = 0 \tag{143}$$

7.44 Species RIP

Name RIP

SBO:0000354 informational molecule segment

Notes

MIRIAM Annotation This biological entity has an unknown relationship with urn:miriam:uniprot:Q13546.

This biological entity is described by urn:miriam:obo.eco:ECO%3A0000313.

Initial amount 1 mol

This species takes part in one reaction (as a reactant in unknown45_1).

$$\frac{d}{dt}\texttt{RIP} = -v_{43} \tag{144}$$

7.45 Species TRADD

Name TRADD

SBO:0000354 informational molecule segment

Notes **Description for ?TRADD, Hs.89862?:** (RefSeq) TNFRSF1A-associated via death domain

MIRIAM Annotation This biological entity is urn:miriam:entrez.gene:8717.

This biological entity is urn:miriam:kegg.genes:hsa%3A8717.

This biological entity is urn:miriam:hgnc:HGNC%3A12030.

This biological entity has an unknown relationship with urn:miriam:uniprot:Q15628.

This biological entity has property urn:miriam:omim:603500.

This biological entity is described by urn:miriam:obo.eco:ECO%3A0000313.

Initial amount 1 mol

This species takes part in one reaction (as a reactant in unknown45_1).

$$\frac{d}{dt}\texttt{TRADD} = -v_{43} \tag{145}$$

7.46 Species MADD

Name MADD

SBO:0000354 informational molecule segment

Notes **Description for ?MADD, DENN, IG20, RAB3GEP?:** (RefSeq) MAP-kinase activating death domain

MIRIAM Annotation This biological entity is urn:miriam:entrez.gene:8567.

This biological entity is urn:miriam:kegg.genes:hsa%3A8567.

This biological entity is urn:miriam:hgnc:HGNC%3A6766.

This biological entity has an unknown relationship with urn:miriam:uniprot:Q8WXG6.

This biological entity has property urn:miriam:omim:603584.

This biological entity is urn:miriam:ensembl:ENSG00000110514.

This biological entity is described by urn:miriam:obo.eco:ECO%3A0000313.

Initial amount 1 mol

This species takes part in one reaction (as a reactant in unknown45_1).

$$\frac{d}{dt}\text{MADD} = -v_{43} \tag{146}$$

7.47 Species TRAF2

Name TRAF2

SBO:0000354 informational molecule segment

Notes **Description for ?TRAF2, MGC:45012, TRAP, TRAP3?:** (RefSeq) TNF receptor-associated factor 2

MIRIAM Annotation This biological entity is urn:miriam:entrez.gene:7186.

This biological entity is urn:miriam:kegg.genes:hsa%3A7186.

This biological entity is urn:miriam:hgnc:HGNC%3A12032.

This biological entity has an unknown relationship with urn:miriam:uniprot:Q12933.

This biological entity has property urn:miriam:omim:601895.

This biological entity is urn:miriam:ensembl:ENSG00000127191.

This biological entity is described by urn:miriam:obo.eco:ECO%3A0000313.

Initial amount 1 mol

This species takes part in one reaction (as a reactant in unknown45_1).

$$\frac{\mathrm{d}}{\mathrm{d}t}\texttt{TRAF2} = -v_{43} \tag{147}$$

7.48 Species response_to_hydrogen_peroxide

Name response to hydrogen peroxide

SBO:0000552 reference annotation

Notes

MIRIAM Annotation This biological entity is described by urn:miriam:obo.eco:ECO%3A0000313.

Initial amount 1 mol

This species does not take part in any reactions. Its quantity does hence not change over time:

$$\frac{\mathrm{d}}{\mathrm{d}t}\texttt{response\_to\_hydrogen\_peroxide} = 0 \tag{148}$$

7.49 Species Acid_Sphingomyelinase

Name Acid_Sphingomyelinase

SBO:0000354 informational molecule segment

Notes

MIRIAM Annotation This biological entity has an unknown relationship with urn:miriam:uniprot:P17405.

This biological entity is described by urn:miriam:obo.eco:ECO%3A0000313.

Initial amount 1 mol

This species takes part in three reactions (as a reactant in unknown20 and as a product in unknown20 and as a modifier in unknown23).

$$\frac{\mathrm{d}}{\mathrm{d}t}\texttt{Acid\_Sphingomyelinase} = v_{20} - v_{20} \tag{149}$$

7.50 Species MKK4_1

Name MKK4

SBO:0000354 informational molecule segment

Notes

MIRIAM Annotation This biological entity has an unknown relationship with urn:miriam:uniprot:P45985.

This biological entity is described by urn:miriam:obo.eco:ECO%3A0000313.

Initial amount 1 mol

This species takes part in one reaction (as a reactant in unknown21).

$$\frac{\mathrm{d}}{\mathrm{d}t}\texttt{MKK4\_1} = -v_{21} \tag{150}$$

7.51 Species EGF

Name EGF

SBO:0000354 informational molecule segment

Notes **Description for ?EGF, HOMG4, URG?:** (RefSeq) epidermal growth factor

MIRIAM Annotation This biological entity is urn:miriam:entrez.gene:1950.

This biological entity is urn:miriam:kegg.genes:hsa%3A1950.

This biological entity is urn:miriam:hgnc:HGNC%3A3229.

This biological entity has an unknown relationship with urn:miriam:uniprot:P01133.

This biological entity has property urn:miriam:omim:131530.

This biological entity is urn:miriam:ensembl:ENSG00000138798.

This biological entity is described by urn:miriam:obo.eco:ECO%3A0000313.

Initial amount 1 mol

This species takes part in two reactions (as a modifier in unknown6, unknown23).

$$\frac{\mathrm{d}}{\mathrm{d}t}\texttt{EGF} = 0 \tag{151}$$

7.52 Species negative_regulation_of_cell_cycle

Name negative_regulation_of_cell_cycle

SBO:0000552 reference annotation

Notes

MIRIAM Annotation This biological entity is described by urn:miriam:obo.eco:ECO%3A0000313.

Initial amount 1 mol

This species does not take part in any reactions. Its quantity does hence not change over time:

$$\frac{\mathrm{d}}{\mathrm{d}t}\texttt{negative\_regulation\_of\_cell\_cycle} = 0 \tag{152}$$

7.53 Species IGF1

Name IGF1

SBO:0000354 informational molecule segment

Notes **Description for ?IGF1, IGF-I, IGF1A, IGFI?:** (RefSeq) insulin-like growth factor 1 (somatomedin C)

MIRIAM Annotation This biological entity is urn:miriam:entrez.gene:3479.

This biological entity is urn:miriam:kegg.genes:hsa%3A3479.

This biological entity is urn:miriam:hgnc:HGNC%3A5464.

This biological entity has an unknown relationship with:

- urn:miriam:uniprot:P05019.
- urn:miriam:uniprot:Q5U743.
- urn:miriam:uniprot:Q59GC5.
- urn:miriam:uniprot:P01343.

This biological entity has property urn:miriam:omim:147440.

This biological entity is urn:miriam:ensembl:ENSG00000017427.

This biological entity is described by urn:miriam:obo.eco:ECO%3A0000313.

Initial amount 1 mol

This species takes part in one reaction (as a modifier in unknown2).

$$\frac{\mathrm{d}}{\mathrm{d}t}\texttt{IGF1} = 0 \tag{153}$$

7.54 Species tumor_necrosis_factor_receptor_activity

Name tumor necrosis factor receptor activity

SBO:0000552 reference annotation

Notes

MIRIAM Annotation This biological entity is described by urn:miriam:obo.eco:ECO%3A0000313.

Initial amount 1 mol

This species does not take part in any reactions. Its quantity does hence not change over time:

$$\frac{d}{dt}\text{tumor\_necrosis\_factor\_receptor\_activity} = 0 \tag{154}$$

7.55 Species AKT1

Name AKT1

SBO:0000354 informational molecule segment

Notes **Description for ?AKT1, AKT, CWS6, PKB, PKB-ALPHA, PRKBA, RAC, RAC-ALPHA?:** (RefSeq) v-akt murine thymoma viral oncogene homolog 1 (EC:2.7.11.1)

MIRIAM Annotation This biological entity has property urn:miriam:ec-code:2.7.11.1.

This biological entity is urn:miriam:entrez.gene:207.

This biological entity is urn:miriam:kegg.genes:hsa%3A207.

This biological entity is urn:miriam:hgnc:HGNC%3A391.

This biological entity has an unknown relationship with:

- urn:miriam:uniprot:B0LPE5.
- urn:miriam:uniprot:P31749.

This biological entity has property urn:miriam:omim:164730.

This biological entity is urn:miriam:ensembl:ENSG00000142208.

This biological entity is described by urn:miriam:obo.eco:ECO%3A0000313.

Initial amount 1 mol

This species takes part in four reactions (as a reactant in unknown25_1, unknown31 and as a product in unknown25_1, unknown31).

$$\frac{d}{dt}\text{AKT1} = v_{25} + v_{29} - v_{25} - v_{29} \tag{155}$$

7.56 Species response_to_UV

Name response to UV

SBO:0000552 reference annotation

Notes

MIRIAM Annotation This biological entity is described by urn:miriam:obo.eco:ECO%3A0000313.

Initial amount 1 mol

This species does not take part in any reactions. Its quantity does hence not change over time:

$$\frac{\mathrm{d}}{\mathrm{d}t}\texttt{response\_to\_UV} = 0 \tag{156}$$

7.57 Species PDGFA

Name PDGFA

SBO:0000354 informational molecule segment

Notes **Description for ?PDGFA, PDGF-A, PDGF1?:** (RefSeq) platelet-derived growth factor alpha polypeptide

MIRIAM Annotation This biological entity is urn:miriam:entrez.gene:5154.

This biological entity is urn:miriam:kegg.genes:hsa%3A5154.

This biological entity is urn:miriam:hgnc:HGNC%3A8799.

This biological entity has an unknown relationship with urn:miriam:uniprot:P04085.

This biological entity has property urn:miriam:omim:173430.

This biological entity is urn:miriam:ensembl:ENSG00000197461.

This biological entity is described by urn:miriam:obo.eco:ECO%3A0000313.

Initial amount 1 mol

This species takes part in two reactions (as a modifier in unknown26, unknown39).

$$\frac{\mathrm{d}}{\mathrm{d}t}\texttt{PDGFA} = 0 \tag{157}$$

7.58 Species MYC

Name MYC

SBO:0000354 informational molecule segment

Notes **Description for ?MYC, MRTL, MYCC, bHLHe39, c-Myc?:** (RefSeq) v-myc avian myelocytomatosis viral oncogene homolog

MIRIAM Annotation This biological entity is urn:miriam:entrez.gene:4609.

This biological entity is urn:miriam:kegg.genes:hsa%3A4609.

This biological entity is urn:miriam:hgnc:HGNC%3A7553.

This biological entity has an unknown relationship with urn:miriam:uniprot:P01106.

This biological entity has property urn:miriam:omim:190080.

This biological entity is urn:miriam:ensembl:ENSG00000136997.

This biological entity is described by urn:miriam:obo.eco:ECO%3A0000313.

Initial amount 1 mol

This species does not take part in any reactions. Its quantity does hence not change over time:

$$\frac{\mathrm{d}}{\mathrm{d}t}\texttt{MYC} = 0 \tag{158}$$

7.59 Species glutathione

Name glutathione

SBO:0000247 simple chemical

Notes

MIRIAM Annotation This biological entity is urn:miriam:cas:17-18-8.

This biological entity is described by urn:miriam:obo.eco:ECO%3A0000313.

Initial amount 1 mol

This species takes part in one reaction (as a modifier in unknown9).

$$\frac{\mathrm{d}}{\mathrm{d}t}\texttt{glutathione} = 0 \tag{159}$$

7.60 Species FAN

Name FAN

SBO:0000354 informational molecule segment

Notes

MIRIAM Annotation This biological entity has an unknown relationship with urn:miriam:uniprot:Q8IW26.

This biological entity is described by urn:miriam:obo.eco:ECO%3A0000313.

Initial amount 1 mol

This species takes part in one reaction (as a reactant in unknown32).

$$\frac{\mathrm{d}}{\mathrm{d}t}\mathtt{FAN} = -v_{30} \tag{160}$$

7.61 Species TNFR1A_1

Name TNFR1A

SBO:0000354 informational molecule segment

Notes

MIRIAM Annotation This biological entity has an unknown relationship with urn:miriam:uniprot:P19438.

This biological entity is described by urn:miriam:obo.eco:ECO%3A0000313.

Initial amount 1 mol

This species takes part in one reaction (as a reactant in unknown32).

$$\frac{\mathrm{d}}{\mathrm{d}t}\mathtt{TNFR1A\_1} = -v_{30} \tag{161}$$

7.62 Species GW4869

Name GW4869

SBO:0000247 simple chemical

Notes

MIRIAM Annotation This biological entity is described by urn:miriam:obo.eco:ECO%3A0000313.

Initial amount 1 mol

This species takes part in one reaction (as a modifier in unknown6).

$$\frac{\mathrm{d}}{\mathrm{d}t}\mathtt{GW4869} = 0 \tag{162}$$

7.63 Species C11AG

Name C11AG

SBO:0000247 simple chemical

Notes

MIRIAM Annotation This biological entity is described by urn:miriam:obo.eco:ECO%3A0000313.

Initial amount 1 mol

This species takes part in one reaction (as a modifier in unknown6).

$$\frac{\mathrm{d}}{\mathrm{d}t}\texttt{C11AG} = 0 \tag{163}$$

7.64 Species TNFalpha_1

Name TNF-alpha

SBO:0000354 informational molecule segment

Notes

MIRIAM Annotation This biological entity has an unknown relationship with urn:miriam:uniprot:P01375.

This biological entity is described by urn:miriam:obo.eco:ECO%3A0000313.

Initial amount 1 mol

This species takes part in two reactions (as a reactant in unknown32, unknown44_1).

$$\frac{\mathrm{d}}{\mathrm{d}t}\texttt{TNFalpha\_1} = -v_{30} - v_{42} \tag{164}$$

7.65 Species MEK2

Name MEK2

SBO:0000354 informational molecule segment

Notes

MIRIAM Annotation This biological entity has an unknown relationship with urn:miriam:uniprot:P36507.

This biological entity is described by urn:miriam:obo.eco:ECO%3A0000313.

Initial amount 1 mol

This species takes part in three reactions (as a product in unknown41, unknown51 and as a modifier in unknown33_1).

$$\frac{\mathrm{d}}{\mathrm{d}t}\mathtt{MEK2} = v_{39} + v_{49} \tag{165}$$

7.66 Species ERK2

Name ERK2

SBO:0000354 informational molecule segment

Notes

MIRIAM Annotation This biological entity has an unknown relationship with urn:miriam:uniprot:P28482.

This biological entity is described by urn:miriam:obo.eco:ECO%3A0000313.

Initial amount 1 mol

This species takes part in six reactions (as a reactant in unknown33_1, unknown34, unknown40 and as a product in unknown33_1, unknown34, unknown40).

$$\frac{\mathrm{d}}{\mathrm{d}t}\mathtt{ERK2} = v_{31} + v_{32} + v_{38} - v_{31} - v_{32} - v_{38} \tag{166}$$

7.67 Species MEK1

Name MEK1

SBO:0000354 informational molecule segment

Notes

MIRIAM Annotation This biological entity has an unknown relationship with urn:miriam:uniprot:Q02750.

This biological entity is described by urn:miriam:obo.eco:ECO%3A0000313.

Initial amount 1 mol

This species takes part in three reactions (as a product in unknown41, unknown51 and as a modifier in unknown34).

$$\frac{\mathrm{d}}{\mathrm{d}t}\mathtt{MEK1} = v_{39} + v_{49} \tag{167}$$

7.68 Species BAD

Name BAD

SBO:0000354 informational molecule segment

Notes **Description for ?BAD, BBC2, BCL2L8?:** (RefSeq) BCL2-associated agonist of cell death

MIRIAM Annotation This biological entity is urn:miriam:entrez.gene:572.

This biological entity is urn:miriam:kegg.genes:hsa%3A572.

This biological entity is urn:miriam:hgnc:HGNC%3A936.

This biological entity has an unknown relationship with urn:miriam:uniprot:Q92934.

This biological entity has property urn:miriam:omim:603167.

This biological entity is urn:miriam:ensembl:ENSG00000002330.

This biological entity is described by urn:miriam:obo.eco:ECO%3A0000313.

Initial amount 1 mol

This species takes part in six reactions (as a reactant in unknown36, unknown37 and as a product in unknown36, unknown37 and as a modifier in unknown5_1, unknown11).

$$\frac{d}{dt}\text{BAD} = v_{34} + v_{35} - v_{34} - v_{35} \tag{168}$$

7.69 Species Acid_Ceramidase

Name Acid_Ceramidase

SBO:0000354 informational molecule segment

Notes

MIRIAM Annotation This biological entity has an unknown relationship with urn:miriam:uniprot:Q13510.

This biological entity is described by urn:miriam:obo.eco:ECO%3A0000313.

Initial amount 1 mol

This species takes part in one reaction (as a modifier in unknown38).

$$\frac{d}{dt}\text{Acid\_Ceramidase} = 0 \tag{169}$$

7.70 Species Free_Fatty_acid

Name Free_Fatty_acid

SBO:0000247 simple chemical

Notes

MIRIAM Annotation This biological entity is described by urn:miriam:obo.eco:ECO%3A0000313.

Initial amount 1 mol

This species takes part in three reactions (as a product in unknown38, unknown39, unknown53).

$$\frac{\mathrm{d}}{\mathrm{d}t}\texttt{Free\_Fatty\_acid} = v_{36} + v_{37} + v_{50} \tag{170}$$

7.71 Species response_to_radiation

Name response to radiation

SBO:0000552 reference annotation

Notes

MIRIAM Annotation This biological entity is described by urn:miriam:obo.eco:ECO%3A0000313.

Initial amount 1 mol

This species does not take part in any reactions. Its quantity does hence not change over time:

$$\frac{\mathrm{d}}{\mathrm{d}t}\texttt{response\_to\_radiation} = 0 \tag{171}$$

7.72 Species MEK1_1

Name MEK1

SBO:0000354 informational molecule segment

Notes

MIRIAM Annotation This biological entity has an unknown relationship with urn:miriam:uniprot:Q02750.
This biological entity is described by urn:miriam:obo.eco:ECO%3A0000313.

Initial amount 1 mol

This species takes part in two reactions (as a reactant in unknown41, unknown51).

$$\frac{\mathrm{d}}{\mathrm{d}t}\texttt{MEK1\_1} = -v_{39} - v_{49} \tag{172}$$

7.73 Species MEK2_1

Name MEK2

SBO:0000354 informational molecule segment

Notes

MIRIAM Annotation This biological entity has an unknown relationship with urn:miriam:uniprot:P36507.

This biological entity is described by urn:miriam:obo.eco:ECO%3A0000313.

Initial amount 1 mol

This species takes part in two reactions (as a reactant in unknown41, unknown51).

$$\frac{\mathrm{d}}{\mathrm{d}t}\texttt{MEK2\_1} = -v_{39} - v_{49} \tag{173}$$

7.74 Species FADD

Name FADD

SBO:0000354 informational molecule segment

Notes **Description for ?FADD, MORT1?:** (RefSeq) Fas (TNFRSF6)-associated via death domain

MIRIAM Annotation This biological entity is urn:miriam:entrez.gene:8772.

This biological entity is urn:miriam:kegg.genes:hsa%3A8772.

This biological entity is urn:miriam:hgnc:HGNC%3A3573.

This biological entity has an unknown relationship with urn:miriam:uniprot:Q13158.

This biological entity has property urn:miriam:omim:602457.

This biological entity is urn:miriam:ensembl:ENSG00000168040.

This biological entity is described by urn:miriam:obo.eco:ECO%3A0000313.

Initial amount 1 mol

This species takes part in two reactions (as a product in unknown42_3, unknown46).

$$\frac{\mathrm{d}}{\mathrm{d}t}\texttt{FADD} = v_{40} + v_{44} \tag{174}$$

7.75 Species RB1

Name RB1

SBO:0000354 informational molecule segment

Notes **Description for ?RB1, OSRC, PPP1R130, RB, p105-Rb, pRb, pp110?:** (RefSeq) retinoblastoma 1

MIRIAM Annotation This biological entity is urn:miriam:entrez.gene:5925.

This biological entity is urn:miriam:kegg.genes:hsa%3A5925.

This biological entity is urn:miriam:hgnc:HGNC%3A9884.

This biological entity has an unknown relationship with:

- urn:miriam:uniprot:P06400.
- urn:miriam:uniprot:Q5VW46.

This biological entity has property urn:miriam:omim:614041.

This biological entity is urn:miriam:ensembl:ENSG00000139687.

This biological entity is described by urn:miriam:obo.eco:ECO%3A0000313.

Initial amount 1 mol

This species takes part in four reactions (as a reactant in unknown43, unknown48 and as a product in unknown43, unknown48).

$$\frac{\mathrm{d}}{\mathrm{d}t}\mathtt{RB1} = v_{41} + v_{46} - v_{41} - v_{46} \quad (175)$$

7.76 Species response_to_heat

Name response to heat

SBO:0000552 reference annotation

Notes

MIRIAM Annotation This biological entity is described by urn:miriam:obo.eco:ECO%3A0000313.

Initial amount 1 mol

This species does not take part in any reactions. Its quantity does hence not change over time:

$$\frac{\mathrm{d}}{\mathrm{d}t}\mathtt{response\_to\_heat} = 0 \quad (176)$$

7.77 Species BAG4

Name BAG4

SBO:0000354 informational molecule segment

Notes **Description for ?BAG4, BAG-4, SODD?:** (RefSeq) BCL2-associated athanogene 4

MIRIAM Annotation This biological entity is urn:miriam:entrez.gene:9530.

This biological entity is urn:miriam:kegg.genes:hsa%3A9530.

This biological entity is urn:miriam:hgnc:HGNC%3A940.

This biological entity has an unknown relationship with urn:miriam:uniprot:O95429.

This biological entity has property urn:miriam:omim:603884.

This biological entity is urn:miriam:ensembl:ENSG00000156735.

This biological entity is described by urn:miriam:obo.eco:ECO%3A0000313.

Initial amount 1 mol

This species takes part in one reaction (as a product in unknown45_1).

$$\frac{\mathrm{d}}{\mathrm{d}t}\mathtt{BAG4} = v_{43} \tag{177}$$

7.78 Species I_kappa_B_alpha_degradation_00

Name I_kappa_B_alpha_(degradation)_0-0

SBO:0000354 informational molecule segment

Notes

MIRIAM Annotation This biological entity has an unknown relationship with urn:miriam:uniprot:P25963.

This biological entity is described by urn:miriam:obo.eco:ECO%3A0000313.

Initial amount 1 mol

This species takes part in one reaction (as a product in unknown47).

$$\frac{\mathrm{d}}{\mathrm{d}t}\mathtt{I\_kappa\_B\_alpha\_degradation\_00} = v_{45} \tag{178}$$

7.79 Species OKADAIC_ACID

Name OKADAIC_ACID

SBO:0000247 simple chemical

Notes

MIRIAM Annotation This biological entity is urn:miriam:cas:78111-17-8.

This biological entity is described by urn:miriam:obo.eco:ECO%3A0000313.

Initial amount 1 mol

This species takes part in one reaction (as a modifier in unknown49).

$$\frac{\mathrm{d}}{\mathrm{d}t}\texttt{OKADAIC\_ACID} = 0 \tag{179}$$

7.80 Species negative_transcription_elongation_factor_activity

Name negative transcription elongation factor activity

SBO:0000552 reference annotation

Notes

MIRIAM Annotation This biological entity is described by urn:miriam:obo.eco:ECO%3A0000313.

Initial amount 1 mol

This species does not take part in any reactions. Its quantity does hence not change over time:

$$\frac{\mathrm{d}}{\mathrm{d}t}\texttt{negative\_transcription\_elongation\_factor\_activity} = 0 \tag{180}$$

7.81 Species ERK1PKC_delta

Name ERK1/PKC_delta

SBO:0000253 non-covalent complex

Notes This species is a group, consisting of 2 components:

- ERK1
- PKC_delta

MIRIAM Annotation This biological entity is described by urn:miriam:obo.eco:ECO%3A0000313.

Initial amount 1 mol

This species takes part in one reaction (as a product in unknown2).

$$\frac{\mathrm{d}}{\mathrm{d}t}\texttt{ERK1PKC\_delta} = v_2 \tag{181}$$

7.82 Species PKC_zetaPAR4

Name PKC_zeta/PAR4

SBO:0000253 non-covalent complex

Notes This species is a group, consisting of 2 components:

- PAR4
- PKC_zeta

MIRIAM Annotation This biological entity is described by urn:miriam:obo.eco:ECO%3A0000313.

Initial amount 1 mol

This species takes part in two reactions (as a product in unknown8_1 and as a modifier in unknown18-_1).

$$\frac{\mathrm{d}}{\mathrm{d}t}\texttt{PKC\_zetaPAR4} = v_8 \tag{182}$$

7.83 Species PKC_zetaceramide

Name PKC_zeta/ceramide

SBO:0000253 non-covalent complex

Notes This species is a group, consisting of 2 components:

- ceramide
- PKC_zeta

MIRIAM Annotation This biological entity is described by urn:miriam:obo.eco:ECO%3A0000313.

Initial amount 1 mol

This species takes part in three reactions (as a product in unknown8_1 and as a modifier in unknown19, unknown25_1).

$$\frac{\mathrm{d}}{\mathrm{d}t}\texttt{PKC\_zetaceramide} = v_8 \tag{183}$$

7.84 Species RelANF_kappa_B1

Name RelA/NF_kappa_B1

SBO:0000253 non-covalent complex

Notes This species is a group, consisting of 2 components:

- NF_kappa_B1
- RELA

MIRIAM Annotation This biological entity is encoded by urn:miriam:kegg.genes:hsa%3A5970.

This biological entity is described by urn:miriam:obo.eco:ECO%3A0000313.

Initial amount 1 mol

This species takes part in two reactions (as a product in unknown47 and as a modifier in unknown10-_2).

$$\frac{\mathrm{d}}{\mathrm{d}t}\texttt{RelANF\_kappa\_B1} = v_{45} \tag{184}$$

7.85 Species Cathepsin_Dceramide

Name Cathepsin_D/ceramide

SBO:0000253 non-covalent complex

Notes This species is a group, consisting of 2 components:

- Cathepsin_D
- ceramide

MIRIAM Annotation This biological entity is described by urn:miriam:obo.eco:ECO%3A0000313.

Initial amount 1 mol

This species takes part in two reactions (as a product in unknown22 and as a modifier in unknown14-_1).

$$\frac{\mathrm{d}}{\mathrm{d}t}\texttt{Cathepsin\_Dceramide} = v_{22} \tag{185}$$

7.86 Species NF_kappa_B1RelAI_kappa_B_alpha

Name NF_kappa_B1/RelA/I_kappa_B_alpha

SBO:0000253 non-covalent complex

Notes This species is a group, consisting of 3 components:

- NF_kappa_B1
- I_kappa_B_alpha
- RELA

MIRIAM Annotation This biological entity is encoded by urn:miriam:kegg.genes:hsa%3A5970.

This biological entity is described by urn:miriam:obo.eco:ECO%3A0000313.

Initial amount 1 mol

This species takes part in one reaction (as a reactant in unknown18_1).

$$\frac{\mathrm{d}}{\mathrm{d}t}\texttt{NF\_kappa\_B1RelAI\_kappa\_B\_alpha} = -v_{18} \tag{186}$$

7.87 Species NF_kappa_B1RelAI_kappa_B_alpha_1

Name NF_kappa_B1/RelA/I_kappa_B_alpha

SBO:0000253 non-covalent complex

Notes This species is a group, consisting of 3 components:

- I_kappa_B_alpha
- RELA
- NF_kappa_B1

MIRIAM Annotation This biological entity is encoded by urn:miriam:kegg.genes:hsa%3A5970.

This biological entity is described by urn:miriam:obo.eco:ECO%3A0000313.

Initial amount 1 mol

This species takes part in two reactions (as a reactant in unknown47 and as a product in unknown18_1).

$$\frac{\mathrm{d}}{\mathrm{d}t}\texttt{NF\_kappa\_B1RelAI\_kappa\_B\_alpha\_1} = v_{18} - v_{45} \tag{187}$$

7.88 Species TNFalphaTNFR1ATRADDMADDcIAP2RIPTRAF2RAIDD

Name TNF-alpha/TNFR1A/TRADD/MADD/cIAP2/RIP/TRAF2/RAIDD

SBO:0000253 non-covalent complex

Notes This species is a group, consisting of 8 components:

- cIAP2
- RAIDD
- TNF-alpha
- TNFR1A
- RIP
- TRADD
- MADD
- TRAF2

MIRIAM Annotation This biological entity is encoded by:

- urn:miriam:kegg.genes:hsa%3A8717.
- urn:miriam:kegg.genes:hsa%3A8567.
- urn:miriam:kegg.genes:hsa%3A7186.

This biological entity is described by urn:miriam:obo.eco:ECO%3A0000313.

Initial amount 1 mol

This species takes part in six reactions (as a product in unknown45_1 and as a modifier in unknown9, unknown14_1, unknown15_2, unknown20, unknown42_3).

$$\frac{\mathrm{d}}{\mathrm{d}t}\texttt{TNFalphaTNFR1ATRADDMADDcIAP2RIPTRAF2RAIDD} = v_{43} \tag{188}$$

7.89 Species PP2A_Heterotrimer

Name PP2A_Heterotrimer

SBO:0000253 non-covalent complex

Notes This species is a group, consisting of 0 components:

MIRIAM Annotation This biological entity is described by urn:miriam:obo.eco:ECO%3A0000313.

Initial amount 1 mol

This species takes part in nine reactions (as a reactant in unknown24, unknown28, unknown49, unknown50 and as a product in unknown24, unknown28, unknown49, unknown50 and as a modifier in unknown35).

$$\frac{d}{dt}\texttt{PP2A\_Heterotrimer} = v_{24} + v_{27} + v_{47} + v_{48} - v_{24} - v_{27} - v_{47} - v_{48} \quad (189)$$

7.90 Species TNFalphaTNFR1AFAN

Name TNF-alpha/TNFR1A/FAN

SBO:0000253 non-covalent complex

Notes This species is a group, consisting of 3 components:

- FAN
- TNF-alpha
- TNFR1A

MIRIAM Annotation This biological entity is described by urn:miriam:obo.eco:ECO%3A0000313.

Initial amount 1 mol

This species takes part in three reactions (as a product in unknown32 and as a modifier in unknown9, unknown20).

$$\frac{d}{dt}\texttt{TNFalphaTNFR1AFAN} = v_{30} \quad (190)$$

7.91 Species FADDCaspase_8

Name FADD/Caspase_8

SBO:0000253 non-covalent complex

Notes This species is a group, consisting of 2 components:

- Caspase_8
- FADD

MIRIAM Annotation This biological entity is encoded by urn:miriam:kegg.genes:hsa%3A8772.

This biological entity is described by urn:miriam:obo.eco:ECO%3A0000313.

Initial amount 1 mol

This species takes part in two reactions (as a reactant in unknown42_3, unknown46).

$$\frac{d}{dt}\texttt{FADDCaspase\_8} = -v_{40} - v_{44} \tag{191}$$

7.92 Species TNFR1ABAG4

Name TNFR1A/BAG4

SBO:0000253 non-covalent complex

Notes This species is a group, consisting of 2 components:

- BAG4
- TNFR1A

MIRIAM Annotation This biological entity is encoded by urn:miriam:kegg.genes:hsa%3A9530.

This biological entity is described by urn:miriam:obo.eco:ECO%3A0000313.

Initial amount 1 mol

This species takes part in one reaction (as a reactant in unknown44_1).

$$\frac{d}{dt}\texttt{TNFR1ABAG4} = -v_{42} \tag{192}$$

7.93 Species TNFR1ABAG4TNFalpha

Name TNFR1A/BAG4/TNF-alpha

SBO:0000253 non-covalent complex

Notes This species is a group, consisting of 3 components:

- TNFR1A
- TNF-alpha
- BAG4

MIRIAM Annotation This biological entity is encoded by urn:miriam:kegg.genes:hsa%3A9530.

This biological entity is described by urn:miriam:obo.eco:ECO%3A0000313.

Initial amount 1 mol

This species takes part in two reactions (as a reactant in unknown45_1 and as a product in unknown44_1).

$$\frac{\mathrm{d}}{\mathrm{d}t}\texttt{TNFR1ABAG4TNFalpha} = v_{42} - v_{43} \tag{193}$$

A Glossary of Systems Biology Ontology Terms

SBO:0000010 reactant: Substance consumed by a chemical reaction. Reactants react with each other to form the products of a chemical reaction. In a chemical equation the Reactants are the elements or compounds on the left hand side of the reaction equation. A reactant can be consumed and produced by the same reaction, its global quantity remaining unchanged.

SBO:0000013 catalyst: Substance that accelerates the velocity of a chemical reaction without itself being consumed or transformed. This effect is achieved by lowering the free energy of the transition state.

SBO:0000027 Michaelis constant: Substrate concentration at which the velocity of reaction is half its maximum. Michaelis constant is an experimental parameter. According to the underlying molecular mechanism it can be interpreted differently in terms of microscopic constants.

SBO:0000176 biochemical reaction: An event involving one or more chemical entities that modifies the electrochemical structure of at least one of the participants.

SBO:0000247 simple chemical: Simple, non-repetitive chemical entity.

SBO:0000253 non-covalent complex: Entity composed of several independant components that are not linked by covalent bonds.

SBO:0000320 product catalytic rate constant: Numerical parameter that quantifies the velocity of product creation by a reversible enzymatic reaction.

SBO:0000321 substrate catalytic rate constant: Numerical parameter that quantifies the velocity of substrate creation by a reversible enzymatic reaction.

SBO:0000324 forward maximal velocity: Limiting maximal velocity of the forward reaction of a reversible enzyme, reached when the substrate is in large excess and all the enzyme is complexed.

SBO:0000325 reverse maximal velocity: Limiting maximal velocity of the reverse reaction of a reversible enzyme, reached when the product is in large excess and all the enzyme is complexed.

SBO:0000354 **informational molecule segment:** Fragment of a macromolecule that carries genetic information.

SBO:0000382 **biochemical exponential coefficient:** Number used as an exponential factor for quantities, expressions or functions

SBO:0000410 **implicit compartment:** A compartment whose existence is inferred due to the presence of known material entities which must be bounded, allowing the creation of material entity pools.

SBO:0000460 **enzymatic catalyst:** A substance that accelerates the velocity of a chemical reaction without itself being consumed or transformed, by lowering the free energy of the transition state. The substance acting as a catalyst is an enzyme.

SBO:0000528 **common modular rate law:** The common modular rate law is a generalised form of reversible Michaelis Menten kinetics, using a denominator where each binding state of the enzyme is represented. It is assumed that substrates and products bind independently and randomly, and that substrates and products cannot be bound at the same time.

SBO:0000552 **reference annotation:** Additional information that supplements existing data, usually in a document, by providing a link to more detailed information, which is held externally, or elsewhere.
